# Oxygenated Theonellastrols: Interpretation of Unusual Chemical Behaviors Using Quantum Mechanical Calculations and Stereochemical Reassignment of 7*α*-Hydroxytheonellasterol

**DOI:** 10.3390/md18120607

**Published:** 2020-11-30

**Authors:** A-Young Shin, Hyi-Seung Lee, Yeon-Ju Lee, Jong Seok Lee, Arang Son, Changhoon Choi, Jihoon Lee

**Affiliations:** 1 Korea Institute of Ocean Science & Technology (KIOST), Busan 49111, Korea; dkdud1624@kiost.ac.kr (A-Y.S.); hslee@kiost.ac.kr (H.-S.L.); yjlee@kiost.ac.kr (Y.-J.L.); jslee@kiost.ac.kr (J.S.L.); 2 Department of Marine Biotechnology, University of Science & Technology, Daejeon 34113, Korea; 3 Department of Radiation Oncology, Samsung Medical Center, Seoul 06351, Korea; arang.son@sbri.co.kr (A.S.); changhoon1.choi@samsung.com (C.C.)

**Keywords:** *Theonella swinhoei*, marine natural product, oxygenated theonellasterol, GIAO NMR chemical shift calculation, anti-inflammatory activity

## Abstract

A total of eight new oxygenated 4-*exo*-methylene sterols, **1**–**8**, together with one artifact **9** and six known sterols **11**–**16**, were isolated from the marine sponge *Theonella swinhoei* collected from the Bohol province in Philippines. Structures of sterols **1**–**8** were determined from 1D and 2D NMR data. Among the sterols, 8*α*-hydroxytheonellasterol (**4**) spontaneously underwent an allylic 1,3-hydroxyl shift to produce 15*α*-hydroxytheonellasterol (**9**) as an artifact; this was rationalized by quantum mechanical calculations of the transition state. In addition, the 1,2-epoxy alcohol subunit of 8*α*-hydroxy-14,15-*β*-epoxytheonellasterol (**5**) was assigned using the Gauge-Independent Atomic Orbital (GIAO) NMR chemical shift calculations and subsequent DP4+ analysis. Finally, comparison of the ^13^C chemical shifts of isolated 7*α*-hydroxytheonellasterol (**6**) with the reported values revealed significant discrepancies at C-6, C-7, C-8, and C-14, leading to reassignment of the C-7 stereochemistry in the known structure.

## 1. Introduction

Marine invertebrates have a broad array of pharmacologically and structurally attractive natural products, some of which exhibit extraordinary potencies and selectivities against human diseases, thus rendering them potential drug candidates [1]. The marine sponge *Theonella swinhoei* has been one of the most diverse sources of natural products. Since the isolation of theonellasterol A from *T. swinhoei* in 1981 [2], various classes of molecules, including polyketide [3,4,5,6,7,8,9,10,11,12], peptides [13,14,15,16,17,18], and sterols [2,19,20,21,22], have been identified and evaluated for their biological properties. The characteristic 4-*exo*-methylene-sterols represent the largest family of secondary metabolites isolated from the genus *Theonella* sponges. To date, at least 37 sterols, including swinhoeisterols [21,22], swinhosterols [19,23], conicasterols [2,24,25,26,27], and theonellasterols [2,25,26,28,29], have been identified through extensive research on this marine invertebrate. Recently, these marine sterols and their synthetic derivatives have been investigated as potent farnesoid-x-receptor (FXR) antagonists to protect the liver from injuries caused due to bile acid overload [25,30].

Generally, structures of the natural sterols can be analyzed from the key NMR (HMBC, NOESY) correlations arising from the methyl groups at C-18 and C-19. However, precise analyses utilizing conventional NMR techniques are problematic in some cases because of the complex overlapping of non-functionalized sp^3^ methylene peaks in the ^1^H NMR spectrum and the absence of ^1^H signals from oxygenated tertiary carbon atoms. Current advances in the prediction of NMR shielding constants employing quantum mechanical calculations have provided alternative tools to clarify the ambiguities in the course of structure determination [31,32]. For instance, the structure of conicasterol F, bearing a tetra-substituted epoxide at C-8 and C-14, was deduced from GIAO calculations of ^13^C NMR chemical shifts and DFT-calculated ROE-distances [26].

**Figure 1:**
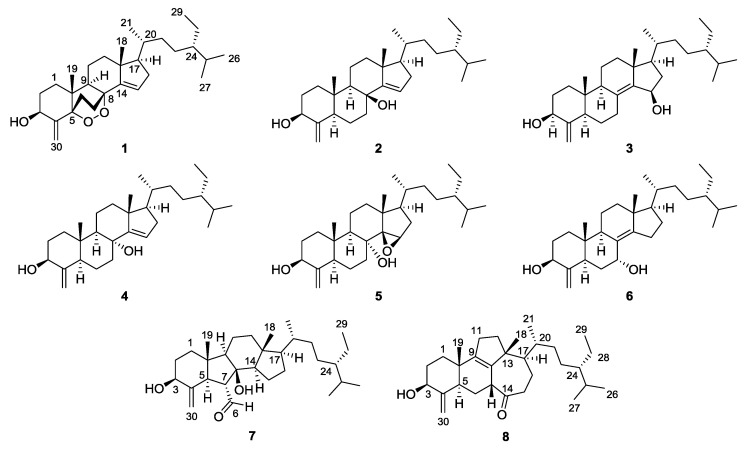
Structures of **1**–**8**.

As a part of our ongoing research to isolate bioactive and structurally interesting natural products, we investigated the metabolites of *T. swinhoei*, collected from the Bohol province in Philippines and identified eight novel theonellasterol analogs **1**–**8** (Figure 1), one artifact **9**, and six known sterols **11**–**16** (Figure S2, Supporting Information). Herein, we report the structural assignments highlighted with DFT calculations to provide a rationale for the unusual chemical behaviors of oxygenated 4-*exo*-methylene sterols. The structure of 8*α*-hydroxy-14,15-*β*-epoxytheonellasterol (**5**) was deduced using GIAO chemical shift calculations. In addition, the structure of 7*α*-hydroxytheonellasterol (**6**), determined by Faulkner and Qureshi in 2000 [28], was reevaluated due to significant discrepancies between the reported ^13^C NMR chemical shifts and the spectroscopic data obtained in this study.

## 2. Results and Discussion

A total of eight new oxygenated theonellasterols **1**–**8** were obtained from the hexane extract of *T. Swinhoei*. Theonellasterol-5,8-oxide (**1**) was isolated as a colorless oil. Its molecular formula was determined to be C_30_H_48_O_3_ by HRESIMS (*m/z* [M + Na]^+^ 479.3485, calcd 479.3501), indicating seven degrees of unsaturation. Inspection of the ^1^H and ^13^C NMR data, including correlations from the HSQC spectrum, provided sufficient information to propose a theonellasterol-type skeleton: two quaternary sp^2^ carbon atoms (*δ*_C_ 152.6, 150.6), an sp^2^ methylene (*δ*_C_ 111.2, *δ*_H_ 5.13, 5.59), an oxymethine (*δ*_C_ 69.8, *δ*_H_ 4.64), two singlet methyl (*δ*_C_ 20.1/*δ*_H_ 0.68, *δ*_C_ 19.0/*δ*_H_ 0.92), three doublet methyl (*δ*_C_ 20.0/*δ*_H_ 0.90, *δ*_C_ 19.6/*δ*_H_ 0.88, *δ*_C_ 19.6/*δ*_H_ 0.98), and one triplet methyl (*δ*_C_ 12.9, *δ*_H_ 0.93) groups. Additionally, an sp^2^ methine (*δ*_C_ 116.8, *δ*_H_ 5.80) and two oxygenated tertiary carbons (*δ*_C_ 91.9, 86.8) were detected as characteristics of compound **1**, suggesting identification of a new analog. While the exocyclic 4,4-di-substituted △^4,^^30^-olefin and endocyclic tetra-substituted olefin at △^8,14^ or △^8,9^ were known to be structural features of theonellasterols, the sp^2^ methine in **1** indicated the presence of an endocyclic tri-substituted olefin that may be generated by an isomerization or rearrangement of the tetra-substituted olefin. Moreover, the additional oxygenated tertiary carbons and a higher degree of hydrogen deficiency (DBE = 7) compared to theonellasterol A (DBE = 6) indicated the existence of an oxygenated theonellasterol framework bearing an additional ring [33,34].

**Figure 2:**
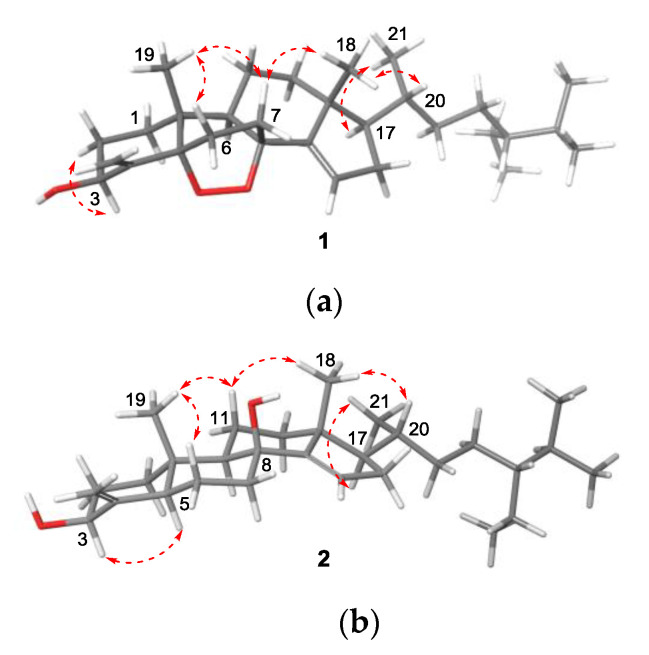
Optimized structures of **1** (**a**) and **2** (**b**) at the mPW1PW91/6-31G* level of theory and key NOESY correlations (arrows).

HMBC correlations from CH_3_-19 (*δ*_H_ 0.68)/H_2_-30 (*δ*_H_ 5.59, 5.13)/H_2_-6 (*δ*_H_ 1.77, 1.33) to *δ*_C_ 91.9 and from H-9 (*δ*_H_ 1.59)/H_2_-11 (*δ*_H_ 1.33, 2H) to *δ*_C_ 86.8 corresponded to the oxygenated tertiary carbon at C-5 and C-8, respectively. The carbon-carbon connectivity for the B-ring was determined by HMBC correlations from H_2_-7 (*δ*_H_ 1.86, 1.43) to C-9 (*δ*_C_ 58.3)/C-6 (*δ*_C_ 30.6), from H_2_-6 (*δ*_H_ 1.77, 1.33) to C-4 (*δ*_C_ 150.6)/C-5 (*δ*_C_ 91.9)/C-10 (*δ*_C_ 44.2), and from H-9 to C-1 (*δ*_C_ 37.8)/C-7 (*δ*_C_ 31.5)/C-10 (*δ*_C_ 44.2). The sp^2^ methine (*δ*_C_ 116.8, *δ*_H_ 5.80) was located at C-15 to form the △^14,15^-olefin, as evident from the ^1^H-^1^H COSY cross peak for H-15–H_2_-16–H-17 and the HMBC correlations from CH_3_-18 (*δ*_H_ 0.92)/H_2_-12 (*δ*_H_ 1.93, 1.23)/H_2_-16 (*δ*_H_ 2.36, 2.01) to C-14 (*δ*_C_ 152.6). Since the carbon framework of **1** turned out to be identical to those of theonellasterols, a covalent bond between the two oxygen atoms at C-5 and C-8 was speculated to fulfill the hydrogen deficiency. The relative configuration of the [2,2,2]-bicyclic B-ring, including those of the consecutive stereocenters at C-13, C-17, and C-20, was determined by NOESY cross peaks between CH_3_-19 and H-6*β* (*δ*_H_ 1.77)/H-7*β* (*δ*_H_ 1.86) and between CH_3_-18 and H-7*β*/H-20 (*δ*_H_ 1.59) (Figure 2). The absolute configuration of **1**, including the configuration of C-24, was deduced to be (3*S*,5*S*,8*R*,9*R*,10*R*,13*R*,17*R*,20*R*,24*S*), considering its biosynthetic correlation with other theonellasterol analogs. Assignment of the 24*S* configuration using the *δ*_H-26_-*δ*_H-27_ values is discussed later in this article.

8*β*-Hydroxytheonellasterol (**2**) was obtained as a colorless oil. Its molecular formula was determined to be C_30_H_50_O_2_ by HRFABMS (*m/z* [M − H_2_O + H]^+^ 425.3780, calcd 425.3783), indicating six degrees of unsaturation. The ^1^H and ^13^C NMR data (Tables S1 and S2, Supporting Information) suggested that compounds **1** and **2** had most of the characteristics in common; however, only one oxygenated tertiary carbon (*δ*_C_ 83.5) was detected. The oxygenated tertiary carbon exhibited correlations with H-7 (*δ*_H_ 2.67, 1.20)/H-15 (*δ*_H_ 5.52) in the HMBC spectrum to be assigned at C-8. The endocyclic olefin was positioned at △^14,15^, based on the interpretation of HMBC correlations from CH_3_-18 (*δ*_H_ 1.11) to C-14 (*δ*_C_ 151.2) and from H-15 (*δ*_H_ 5.52) to C-8/C-13 (*δ*_C_ 47.7)/C-14, as well as a spin system for H-15–H-16–H-17 in the ^1^H-^1^H COSY spectrum. Careful inspection of the ^1^H NMR spectrum and the NOESY spectrum revealed that the axial H-11*β* (*δ*_H_ 1.82) and H-6*β* (*δ*_H_ 1.89) were more deshielded than equatorial H-11*α* (*δ*_H_ 1.30) and H-6*α* (*δ*_H_ 1.44) (Figure 2). Additionally, axially oriented CH_3_-18 and CH_3_-19 (*δ*_H_ 0.97) were shifted downfield compared to those in the reported theonellasterols [25]. These features accounted for the *β*-orientation of the hydroxyl group at C-8, initiating additional 1,3-diaxial interactions [35,36]. 

15*β*-Hydroxytheonellasterol (**3**) was isolated as a colorless oil. Its molecular formula was determined to be C_30_H_50_O_2_ by HRESIMS (*m/z* [M − H_2_O + H]^+^ 425.3789, calcd 425.3783). The 1D and 2D NMR spectra of **3** were almost identical to those of theonellasterol A. However, an additional oxymethine (*δ*_C_ 70.3, *δ*_H_ 4.64) was detected, of which the location was assigned as C-15 by HMBC correlations from *δ*_H_ 4.64 to C-13 (*δ*_C_ 43.8)/C-17 (*δ*_C_ 54.2) and from H_2_-16 (*δ*_H_ 1.95, 1.60) to *δ*_C_ 70.3. The *β*-orientation of the hydroxyl group at C-15 was established from the NOESY correlation between H-15 and H-17 (*δ*_H_ 1.60).

**Figure 3:**
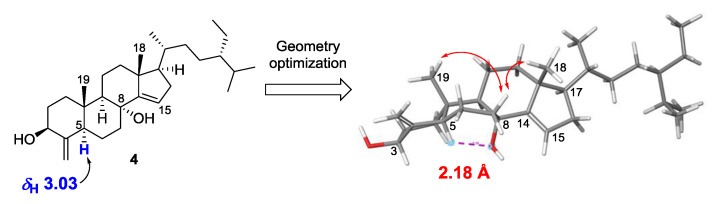
The optimized structure of **4** at the mPW1PW91/6-31G* level of theory and the calculated atomic distance between H-5 and OH-8.

8*α*-Hydroxytheonellasterol (**4**) was obtained as a chemically labile compound, and reliable HRMS data could not be obtained. However, the 1D and 2D NMR spectra of **4** suggested the presence of an oxygenated theonellasterol scaffold bearing an oxygenated tertiary carbon (*δ*_C_ 86.6) and an sp^2^ methine (*δ*_C_ 123.2, *δ*_H_ 5.61). HMBC correlations from H_2_-6 (*δ*_H_ 1.54)/H-9 (*δ*_H_ 2.27) to the carbon at *δ*_C_ 86.6 and from the proton at *δ*_H_ 5.61 to C-13 (*δ*_C_ 48.0)/C-16 (*δ*_C_ 35.2)/C-17 (*δ*_C_ 60.0) revealed that the oxygenated tertiary carbon and the sp^2^ methine group were located to C-8 and C-15, respectively. This further suggested that **4** was the C-8 epimer of 8*β*-hydroxytheonellasterol (**2**). For this case, the extra 1,3-diaxial interaction, which was observed in the case of **2**, was undetectable from H_2_-6 (*δ*_H_ 1.67, 1.54), H_2_-11 (*δ*_H_ 1.49, 2H), CH_3_-18 (*δ*_H_ 0.83), and CH_3_-19 (*δ*_H_ 0.68), supporting the *α*-orientation of OH-8. Instead, the ^1^H chemical shift of H-5 (*δ*_H_ 3.03) was further downfield compared to those of the reported sterols [25], and H-7*β* (*δ*_H_ 1.76) exhibited NOESY correlations with Me-18 and Me-19. This phenomenon could be rationalized using quantum mechanical calculations. Geometry optimization of **4** at the mPW1PW91/6-31G* level of theory revealed a boat conformation for the B-ring to initiate 1,4-flagpole interactions between H-5 and OH-8*α* (d = 2.18 Å), resulting in the downfield shift of H-5 (Figure 3). Additionally, atomic distances from H-7 to H-18 and H-19 were measured as 2.07 Å and 3.91 Å, respectively, which are close enough to exhibit NOESY correlations. 

**Figure 4:**
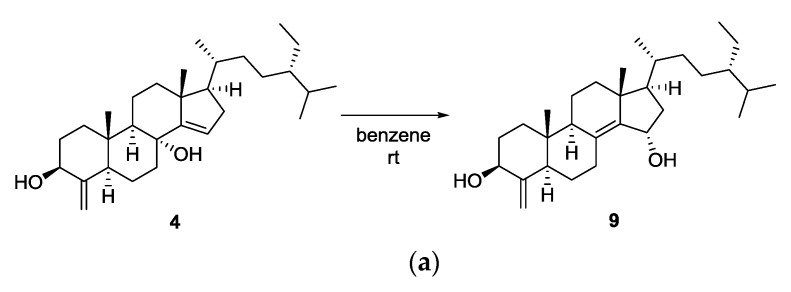

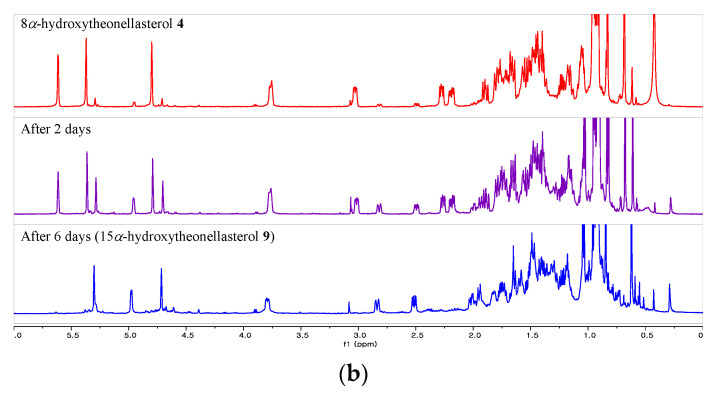
(**a**) The spontaneous transformation of 8*α*-hydroxytheonellasterol (**4**) to an artifact, 15*α*-hydroxytheonellasterol (**9**). (**b**) Time dependent ^1^H NMR spectrum in C_6_D_6_ indicating the transformation.

Due to its labile nature, sterol **4** was entirely decomposed into a complex mixture of unidentifiable compounds within several days. However, extended storage in benzene afforded an artifact as a single compound (Figure 4). Its molecular formula was determined to be C_30_H_50_O_2_ by HRFABMS (*m/z* [M − H_2_O + H]^+^ 425.3781, calcd 425.3783). The 1D and 2D NMR data were almost identical to those of compound **3**, except for the deshielded oxymethine (*δ*_C_ 84.4, *δ*_H_ 4.97). The oxymethine exhibited a ^1^H-^1^H COSY cross peak with H_2_-16 (*δ*_H_ 1.49) and HMBC correlations with C-13 (*δ*_C_ 43.5)/C-14 (*δ*_C_ 141.1)/C-17 (*δ*_C_ 54.1) to be assigned at C-15. Based on the stereochemistry of **3**, the artifact was determined to be 15*α*-hydroxytheonellasterol (**9**), which was produced through an allylic 1,3-hydroxyl migration of **4**. 

**Figure 5:**
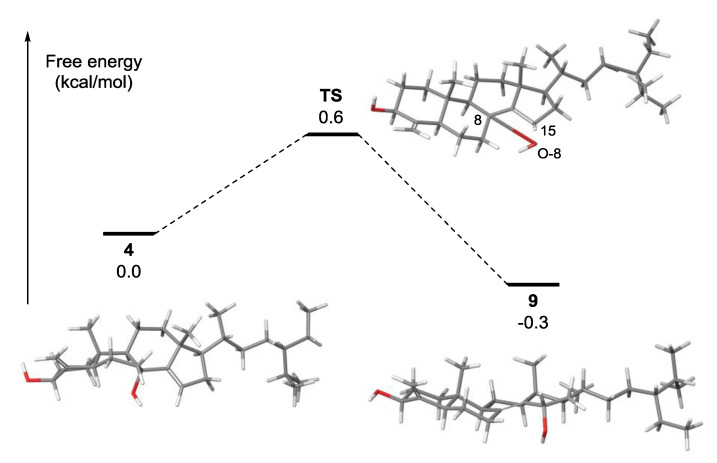
Free energy diagram for 1,3-hydroxyl migration in 8*α*-hydroxy-theonellasterol (**4**) to generate 15*α*-hydroxytheonellasterol (**9**). Geometry optimizations of compounds **4**, **9**, and the transition state (**TS**) were performed at the mPW1PW91/6-31G* level of theory.

Calculation of transition state energy using the Linear Synchronous Transit (LST) method revealed that the energy barrier for the transformation of **4** to **9** was only 0.6 kcal/mol, which can explain the instability of **4** (Figure 5). The structure of the transition state (TS) was turned out to be almost identical to that of **9**. In the transition state, the B-ring was flipped to a chair-like conformation, bringing O-8 and sp^2^ C-15 (1.43 Å) in close proximity. The atomic distance between C-8 and O-8 in TS was measured to be 3.09 Å, suggesting that the C-8–O-8 bond was actually broken before the TS to form a new C–O bond at C-15. In addition, the C-8–C-14 bond length was estimated to be 1.34 Å, indicating olefin migration from △^14,15^ to △^8,14^ (Figure S4, Supporting Information). Although **9** was slightly more stable than **4** at rt (ΔG° = −0.3 kcal/mol), the low activation energy and the formation of a more rigid tetra-substituted olefin perhaps shifted the chemical equilibrium toward **9**. 

8*α*-Hydroxy-14,15-*β*-epoxy-theonellasterol (**5**) was isolated as an amorphous powder. Its molecular formula was determined to be C_30_H_50_O_3_ (*m/z* [M + Na]^+^ 481.3659, calcd 481.3658) by HRESIMS, indicating six degrees of unsaturation. The 1D and 2D NMR data of **5** revealed an oxygenated theonellasterol framework bearing an additional oxymethine (*δ*_C_ 58.8, *δ*_H_ 3.33) and two oxygenated tertiary carbons (*δ*_C_ 75.9, 71.3). In this case, only one olefin (*δ*_C_ 154.2, 103.3), corresponding to 4-*exo*-methylene, was found, indicating the presence of an additional ring to satisfy the unsaturation index. HMBC correlations from H_2_-7 (*δ*_H_ 1.82, 1.60) to C-8 (*δ*_C_ 71.3), from H_2_-16 (*δ*_H_ 1.96, 0.93) to C-14 (*δ*_C_ 75.9)/C-15 (*δ*_C_ 58.8), and from Me-18 (*δ*_H_ 0.71) to C-14 (*δ*_C_ 75.9) indicated the presence of a 1,2-epoxy alcohol moiety within the C-8–C-14–C-15. The upfield shift of C-15 oxymethine (*δ*_C_ 58.8) and the unusual HMBC correlations from the hydroxyl peak at *δ*_H_ 3.47 to C-7 (*δ*_C_ 28.3)/C-8 (*δ*_C_ 71.3)/C-14 (*δ*_C_ 75.9) suggested the presence of an 8-hydroxy-14,15-epoxide subunit. Additionally, *β*-orientation of the epoxide was assigned by the NOESY cross peak between H-17 (*δ*_H_ 1.41) and H-15 (Figure S3, Supporting Information), and the downfield shift of H-5 (*δ*_H_ 3.35) was perhaps attributable to an *α*-orientation of the C-8 hydroxyl group, similar to that of 8*α*-hydroxytheonellasterol (**4**). 

Due to the limited spectroscopic data for the 1,2-epoxy alcohol subunit of **5**, GIAO NMR chemical shift calculations were employed to support the assignments (Table 1). Although our observations suggested the maximum possibility of the 14,15-epoxide isomers **5**–**I** and **5**–**II**, the formation of 8,14-epoxide isomers **5**–**III** and **5**–**IV** could not be ruled out. The ^13^C NMR chemical shift calculations of the four sets of 8,14,15-isomers using the mPW1PW91/6-31G** level of theory and subsequent DP4+ analysis indicated 100% probability of 8*α*-hydroxy-14,15-*β*-epoxy-isomer **5**–**I** [26]. The correlation coefficient (R^2^) in the regression analysis of the experimental versus calculated ^13^C chemical shifts of **5**–**I** was calculated to be 0.9908, indicating that the structure assignment was highly reliable. As anticipated from the downfield shift of H-5, the B-ring in the optimized structure adopted a boat conformation to rationalize the downfield shift of H-5 by 1,4-flagpole interactions (Table S4, Supporting Information).

Compound **6** was isolated as a colorless needle-shaped solid. The molecular formula was determined to be C_30_H_50_O_2_ by HRESIMS (*m/z* [M + Na]^+^ 465.3705, calcd 465.3709), indicating six degrees of unsaturation. Comparison of the NMR spectra of **6** with the previously reported data revealed an oxygenated theonellasterol-type framework bearing an additional oxymethine (*δ*_C_ 66.7, *δ*_H_ 4.64). The HMBC correlation from H_2_-6 (*δ*_C_ 1.77, 1.59) to *δ*_C_ 66.7, as well as the ^1^H-^1^H COSY cross peak between the protons at *δ*_H_ 4.64 and H_2_-6, suggested that the oxymethine was positioned at C-7.

**Figure 6:**
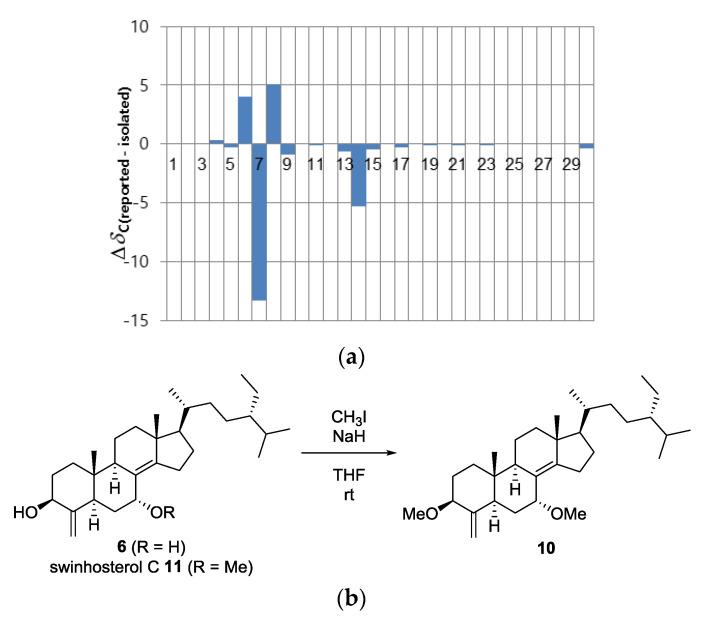

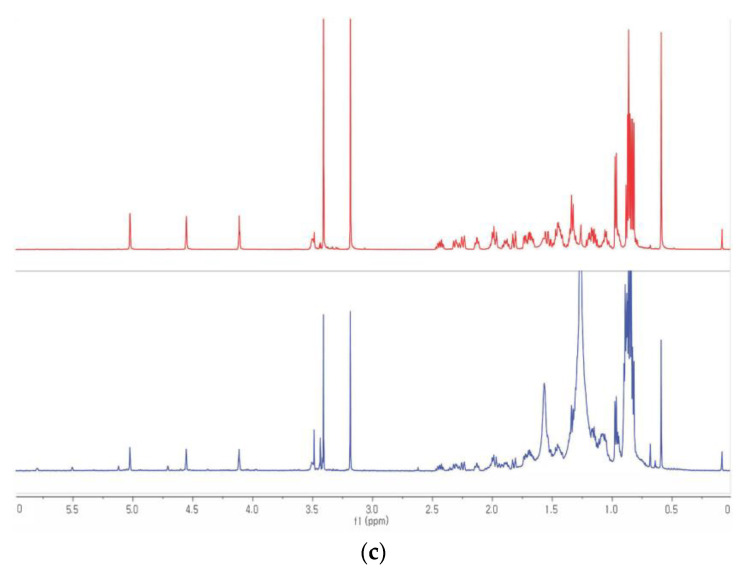
Structure confirmation of 7*α*-hydroxytheonellasterol (**6**): (**a**) Difference between the ^13^C chemical shifts of **6** and the reported values in CDCl_3_. (**b**) Methylation of **9** and swinhosterol C (**11**). (**c**) ^1^H NMR spectrum of **10** synthesized from **6** (red) and ^1^H NMR spectrum of crude **10** synthesized from swinhosterol C (**11**) (blue) in C_6_D_6_.

**Figure 7:**
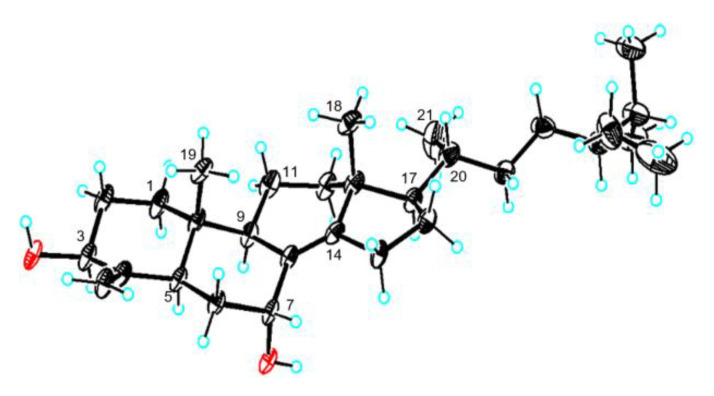
ORTEP drawing of compound **6** based on X-ray data.

Because the isolation of 7*α*-hydroxytheonellasterol was reported in 2000 by Faulkner and Qureshi [28], compound **6** was initially considered to be 7*β*-hydroxytheonellasterol, as deduced from the comparison of ^1^H and ^13^C chemical shifts (Figure 6a). However, a lack of NOESY signals corresponding to H-7 led us to synthesize 3,7-dimethyl ether **10** from **6**. Surprisingly, the NOESY data of **10** indicated a correlation between OMe-7 (*δ*_H_ 3.14) and H-9 (*δ*_H_ 2.29), supporting the *α*-orientation of the C-7 hydroxyl group. In addition, methylation of swinhosterol C (**11**), known as 7*α*-OMe-theonellasterol, afforded a compound that was spectroscopically identical to **10** (Figure 6b,c). Single crystal X-ray diffraction of **6** further confirmed a 7*α*-hydroxytheonellasterol structure (Figure 7). Considering the large differences in the ^13^C chemical shifts at C-6, C-7, C-8, and C-14, we speculate that the previously reported compound is the 7*β*-epimer of **6**.

8*β*-Hydroxy-7*α*-formyl-B-northeonellasterol (**7**) was isolated as a colorless oil. Its molecular formula was determined to be C_30_H_50_O_3_ by HRESIMS (*m/z* [M + Na]^+^ 481.3660, calcd 481.3658). Analysis of the 1D and 2D NMR data of **7** revealed an oxygenated theonellasterol-like skeleton bearing an aldehyde (*δ*_C_ 204.2, *δ*_H_ 9.75) and an additional oxygenated tertiary carbon (*δ*_C_ 87.2). Since the aldehyde moiety is known as a unique feature of 8*β*-hydroxy-B-norconicasta-6*α*-aldehyde among the sterols isolated from *T. swinhoei* [37], the 6/5/6/5-fused cyclic backbone of **7** was assigned by comparing the NMR data. Compound **7** could be differentiated from 8*β*-hydroxy-B-norconicasta-6*α*-aldehyde in the ethyl substituent at C-24, which was assigned based on the HMBC correlations from a triplet methyl group (*δ*_H_ 0.95) to C-24 (*δ*_C_ 46.9)/C-28 (*δ*_C_ 23.8). 

28-Homoswinhoeisterol (**8**) was isolated as a yellow oil. The molecular formula was determined to be C_30_H_48_O_2_ by HRESIMS (*m/z* [M + Na]^+^ 465.3549, calcd 463.3552). The IR spectrum of **8** clearly indicated the presence of a hydroxyl group (3343 cm^−1^) and a ketone group (1593 cm^−1^). The features of the IR and NMR spectra of this compound were almost identical to those of swinhoeisterol A. The only difference was found in the triplet methyl group (*δ*_H_ 0.84), which was involved in a spin system for H-24–H_2_-28–CH_3_-29, as evident from the ^1^H-^1^H COSY spectrum. This suggested that the ethyl group was located at C-24. In addition, the plausible biogenetic pathway reported by Zhang et al. [21] indicated that compound **8** could be originated from swinhosterol A through an intramolecular aldol-type reaction, which strongly suggested that the absolute configuration of **8** is (3*S*,5*R*,7*R*,10*S*,13*R*,17*R*,20*R*,24*S*). To date, 28-homoswinhoiesterol (**8**) is the only 6/6/5/7-fused cyclic sterol derived from C_30_ sterols such as theonellasterol A and swinhosterol A.

Generally, the 24*S* configurations of 24-ethyl-sterol analogs are deduced from the ^13^C chemical shift differences between CH_3_-26 and CH_3_-27 [38]. However, the differences for compounds **2** (0.6 ppm), **5** (0.7 ppm), and **7** (0.8 ppm) were not significant enough for determining the configuration of C-24, and hence, a complementary method was required for the assignment. To establish a universal database using ^1^H NMR data, the absolute values of *δ*_H-26_-*δ*_H-27_ were obtained from the sets of sterol-type compounds bearing a (21*R*,24*R*) or (21*R*,24*S*)-21,26-dimethyl-24-ethylhexane side chain (Table S11, Supporting Information). Values calculated for the (21*R*,24*R*)-set were higher than 0.04 ppm, whereas those for the (21*R*,24*S*)-set were smaller than 0.04 ppm. The validity of our database was evaluated using six known 24*S*-ethyl-sterols isolated in this study: theonellasterol A (**12**), E (**13**), G (**14**), and K (**15**); swinhosterol A (**16**) and C (**11**). In all the cases, the differences were smaller than 0.03 ppm to prove the reliability of the database. Further, this method was extended to the new compounds **1**–**8**. The differences for all of them were in the desirable range (<0.03 ppm), confirming their 24*S* configuration (Figure S8, Supporting Information).

**Figure 8:**
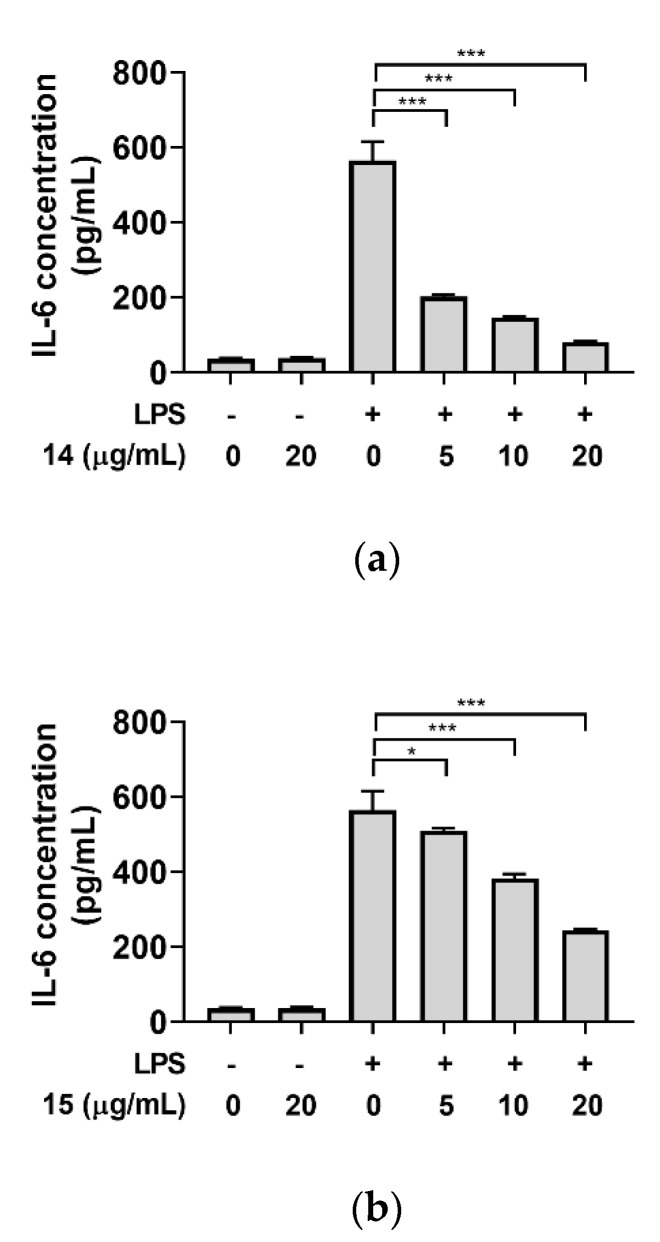
Anti-inflammatory effect of (**a**) theonellasterol G (**14**) and (**b**) theonellasterol K (**15**). RAW264.7 cells were pre-treated with indicated concentrations of theonellasterols for 3 h, followed by treatment with 5 ng/mL of lipopolysaccharide (LPS). After 24 h of incubation, secreted interleukin-6 (IL-6) levels were determined as described in Experimental section. Data are presented as mean ± standard deviation (*n* = 4). Statistical analysis was performed using one-way analysis of variance with Dunnett’s post-hoc analysis. * *p* < 0.05; *** *p* < 0.001 vs. LPS-stimulated group.

With the theonellasterol analogs in hand, their anti-inflammatory activities were investigated using the murine macrophage RAW264.7 cells. Treatment with lipopolysaccharide (LPS) in RAW264.7 macrophages stimulates secretion of pro-inflammatory cytokines, including interleukin-6 (IL-6). Levels of IL-6 secreted by the cells were quantified by the enzyme-linked immunosorbent assay (ELISA) method. Unfortunately, useful levels of biological properties for the new oxygenated theonellasterols (**1**–**9**) were unidentified. However, theonellasterol G (**14**) showed a moderate anti-inflammatory activity with an IC_50_ of 4.4 μg/mL (9.2 μM), and theonellasterol K (**15**) exhibited a weak anti-inflammatory activity with an IC_50_ of 16.7 μg/mL (35.2 μM) (Figure 8).

## 3. Materials and Methods 

### 3.1. General Experimental Procedures

Specific optical rotations were obtained on a Rudolph Research Analytical (Autopol III) polarimeter (Rudolph Research Analytical, Hackettstown, NJ, USA). IR spectra were recorded on a JASCO FT/IR-4100 spectrophotometer (JASCO Corporation, Tokyo, Japan). The 1D (^1^H and ^13^C) and 2D (COSY, HSQC, HMBC, and NOESY) NMR spectra were taken in C_6_D_6_ or CDCl_3_ using Bruker 600 MHz spectrometer (Bruker BioSpin GmbH, Rheinstetten, Germany) at 297.1 K. ^1^H NMR spectra were collected after 64 scans, and ^13^C NMR spectra were collected at a range of 10,000~15,000 scans depending on the sample concentrations. The mixing time for NOESY experiments was set as 0.3 s. Chemical shifts are reported in parts per million relative to C_6_D_6_ (*δ*_H_ 7.16, *δ*_C_ 128.4) and CDCl_3_ (*δ*_H_ 7.26, *δ*_C_ 77.1). High resolution mass-spectra were obtained on a Waters Q-TOF spectrometer (Waters Corporation, Milford, MA, USA) equipped with an ESI source and a JEOL JMS-700 spectrometer (JEOL Ltd., Tokyo, Japan) with an FAB Source at Korea Basic Science Institute (KBSI) (Seoul, Republic of Korea). MPLC was performed using the TELEDYNE ISCO CombiFlash Companion with the TELEDYNE ISCO RediSep Normal-phase Silica Flash Column (Teledyne ISCO, Lincoln, NE, USA). HPLC was performed on a PrimeLine Binary pump (Analytical Scientific Instruments, Inc., El Sobrante, CA, USA) utilizing Silica columns (YMC-Pack Silica, 250 × 10 mm I.D. or 250 × 4.6 mm I.D., 5 µm; YMC Co. Ltd., Kyoto, Japan), the Shodex RI-101 (Shoko Scientific Co. Ltd., Yokohama, Japan), or the UV-M201. 

### 3.2. Biological Material

The biological material was collected in March 2016 from the Bohol province in Philippines (9°43′31.86″ N, 124°32′35.57″ E) at a depth of 15 m using scuba diving. The sponge was kept frozen at −20 °C until identified as *Theonella swinhoei* and chemically analyzed. A voucher sample (163PIL-102) has been stored at the marine biotechnology center, Korea Institute of Ocean Science & Technology (KIOST). 

### 3.3. Extraction and Isolation

The specimen (wet wt. 1.8 kg) was lyophilized and extracted with MeOH (2.5 L × 3) and CH_2_Cl_2_ (2.5 L × 3) repeatedly at room temperature. The extracts were combined and then concentrated under reduced pressure. The residue was partitioned with *n*-butanol (7.0 L) and water (5.0 L) to yield 55.43 g of organic soluble material. The *n*-butanol layer was further partitioned between *n*-hexane (2.0 L) and 15% aqueous methanol (2.0 L). The hexane fraction was concentrated and subjected to flash column chromatography over SiO_2_ (0.040–0.063 mm, 230–400 mesh) with a stepwise gradient solvent system (100%, 93.7%, 90%, 83%, 80%, 75%, 50% hexane/EtOAc, 100% EtOAc). 

The 90% and 83% hexane fractions were combined (3.22 g) and separated using MPLC on SiO_2_ with a gradient solvent system from 100% hexane to 100% EtOAc over 40 min to afford seven subfractions (based on TLC analysis). The third subfraction gave theonellasterol A (**12**) (1.00 g) as a pure compound without further purification. The sixth subfraction was separated using HPLC (hexane/EtOAc = 5/1) to yield compounds **2** (8.9 mg, *t*_R_ = 26 min) and **1** (3.5 mg, *t*_R_ = 28 min). 

The 80% hexane fraction (273.4 mg) was directly separated using HPLC (hexane/EtOAc = 4/1) to yield compound **8** (31.4 mg, *t*_R_ = 21 min) and compound **5** (1.2 mg, *t*_R_ = 26 min). The 75% and 50% hexane fractions were combined (786.0 mg) and separated using MPLC on SiO_2_ with a gradient solvent system from 100% hexane to 100% EtOAc over 40 min to afford six subfractions (based on TLC analysis). The third subfraction (200.0 mg) was separated using HPLC (hexane/acetone = 6/1) to yield swinhosterol C (**11**) (1.7 mg, *t*_R_ = 17 min), **4** (1.2 mg, *t*_R_ = 20 min), and theonellasterol K (**15**) (30.3 mg, *t*_R_ = 24 min). The fourth subfraction (57.2 mg) was separated using HPLC (CH_2_Cl_2_/MeOH = 100/1) to yield swinhosterol A (**16**) (12.0 mg, *t*_R_ = 26 min), **7** (1.6 mg, *t*_R_ = 28 min), and **3** (3.5 mg, *t*_R_ = 52 min). The fifth subfraction (72.9 mg) was separated using HPLC (hexane/acetone = 6/1) to yield **6** (3.1 mg, *t*_R_ = 30 min), and theonellasterol G (**14**) (5.9 mg, *t*_R_ = 52 min). 

The 100% EtOAc fraction (372.3 mg) was separated using MPLC with a gradient solvent system from 70% hexane to 100% EtOAc to afford four subfractions (based on TLC analysis). The fourth subfraction (78.1 mg) was separated using HPLC (hexane/acetone = 4/1) to yield theonellasterol E (**13**) (5.0 mg, *t*_R_ = 40 min).

Theonellasterol-5,8-oxide (**1**): colorless oil; [*α*${]}_{D}^{25}$ + 20.0 (*c* 0.1, MeOH); IR (ATR) *ν*_max_ 3417, 2957, 2851, 1738, 1455, 1027 cm^−1^; ^1^H NMR and ^13^C NMR, see Tables S1 and S2, Supporting Information; HRESIMS *m/z* 479.3485 [M + Na]^+^ (calcd for C_30_H_48_O_3_Na, 479.3501).

8*β*-Hydroxytheonellasterol (**2**): colorless oil; [*α*${]}_{D}^{25}$ + 20.0 (*c* 0.1, MeOH); IR (ATR) *ν*_max_ 3353, 2929, 1751, 1489, 1410, 1101 cm^−1^; ^1^H NMR and ^13^C NMR, see Tables S1 and S2, Supporting Information; HRFABMS *m/z* 425.3780 [M − H_2_O + H]^+^ (calcd for C_30_H_49_O, 425.3783). 

15*β*-Hydroxytheonellasterol (**3**): colorless oil; [*α*${]}_{D}^{25}$ + 40.0 (*c* 0.1, MeOH); IR (ATR) *ν*_max_ 3345, 2963, 2938, 2871, 1711, 1379, 1039 cm^−1^; ^1^H NMR and ^13^C NMR, see Table S1 and S2, Supporting Information; HRESIMS *m/z* 425.3789 [M − H_2_O + H]^+^ (calcd for C_30_H_49_O, 425.3783).

8*α*-Hydroxytheonellasterol (**4**): colorless oil; ^1^H NMR and ^13^C NMR, see Tables S1 and S2, Supporting Information.

8*α*-Hydroxy-14,15-*β*-epoxy-theonellasterol (**5**): amorphous powder; [*α*${]}_{D}^{25}$ + 30.0 (*c* 0.1, MeOH); IR (ATR) *ν*_max_ 3567, 2954, 2929, 2861, 1727, 1377, 1254, 1035 cm^−1^; ^1^H NMR and ^13^C NMR, see Table S1 and S2, Supporting Information; HRESIMS *m/z* 481.3659 [M + Na]^+^ (calcd for C_30_H_50_O_3_Na, 481.3658).

7*α*-Hydroxytheonellasterol (**6**): colorless needle-shaped solid; [*α*${]}_{D}^{25}$ + 6.67 (*c* 0.1, MeOH); IR (ATR) *ν*_max_ 3359, 2929, 1416, 1333, 1100 cm^−1^; ^1^H NMR and ^13^C NMR, see Tables S1 and S2, Supporting Information; HRESIMS *m/z* 465.3705 [M + Na]^+^ (calcd for C_30_H_50_O_2_Na, 465.3709).

8*β*-Hydroxy-7*α*-formyl-B-northeonellasterol (**7**): colorless oil; [*α*${]}_{D}^{25}$ + 40.0 (*c* 0.1, MeOH); IR (ATR) *ν*_max_ 3434, 2933, 2865, 1710, 1458, 1374, 1031 cm^−1^; ^1^H NMR and ^13^C NMR, see Tables S1 and S2, Supporting Information; HRESIMS *m/z* 481.3660 [M + Na]^+^ (calcd for C_30_H_50_O_3_Na, 481.3658).

28-Homoswinhoeisterol (**8**): yellow oil; [*α*${]}_{D}^{25}$ + 100.0 (*c* 0.58, CHCl_3_); IR (ATR) *ν*_max_ 3343, 2936, 1593, 1458, 1413, 1120, 1042 cm^−1^; ^1^H NMR and ^13^C NMR, see Table S1 and S2, Supporting Information; HRESIMS *m/z* 465.3549 [M + Na]^+^ (calcd for C_30_H_48_O_2_Na, 463.3552).

15*α*-Hydroxytheonellasterol (**9**): colorless oil; [*α*${]}_{D}^{25}$ + 76.6 (*c* 0.1, MeOH); IR (ATR) *ν*_max_ 3367, 2961, 2925, 2872, 1702, 1458, 1381 cm^−1^; ^1^H NMR and ^13^C NMR, see Tables S1 and S2, Supporting Information; HRFABMS *m/z* 425.3781 [M − H_2_O + H]^+^ (calcd for C_30_H_49_O, 425.3783).

Theonellastrol-3,7-dimethyl ether (**10**): white powder; [*α*${]}_{D}^{25}$ + 23.33 (*c*, 0.1, MeOH); IR (ATR) *ν*_max_ 2957, 2953, 2872, 2353, 1593, 1102 cm^−1^; ^1^H NMR and ^13^C NMR, see Supporting Information; HRESIMS *m/z* 493.4009 [M + Na]^+^ (calcd for C_32_H_54_O_2_Na, 493.4016).

### 3.4. ^13^C Chemical Shift Calculations

The conformational searches were performed using the Macromodel software (Maestro Materials Science 3.7.013 based on Maestro Core 12.3.013, MMshare Version 4.9.013, Release 2020-1, Platform Windows-x64; New York, NY, USA). The conformers within an energy threshold of 5 kJ/mol were optimized employing DFT calculations at the mPW91PW1/6-3lG* level of theory to estimate gas phase energies and Gibbs free energies. All of the optimizations were performed at “fine” grid density and “ultrafine” accuracy level. The structure that has the lowest gas phase energy was selected, and NMR shielding constants were calculated with the mPW91PW1/6-3lG**/CPCM benzene basis set. The calculated ^13^C chemical shifts of compounds **5**–**I**–**IV** were referenced to the ^13^C chemical shift of tetramethylsilane (TMS), computed with the same level of theory (for the details, see Supporting Information).

### 3.5. IL-6 Assay

The murine macrophage RAW264.7 cells were obtained from Dong Hyun Sohn, Pusan National University, Yangsan, South Korea. The RAW264.7 cells were cultured in Dulbecco’s Modified Eagle Medium (DMEM) supplemented with 10% fetal bovine serum (FBS) and antibiotics. For determining interleukin-6 (IL-6) production, RAW264.7 cells were pre-treated with eight new sterols (**1**–**3**, **5**–**9**) and six known sterols (**11**–**16**) at various concentrations for 3 h, and then were incubated with or without 5 ng/mL of lipopolysaccharide (LPS; eBioscience, San Diego, CA, USA). After 24 h of incubation, the supernatant was collected and subjected to enzyme-linked immunosorbent assay (ELISA). The levels of IL-6 were measured by using mouse IL-6 Quantikine ELISA kit (R&D systems, Minneapolis, MN, USA) according to the manufacturer’s instructions. 

## 4. Conclusions

A total of eight new oxygenated 4-*exo*-methylene sterols **1**–**8** and six known sterols (**11**–**16**) were isolated from *T. swinhoei*. The C-7 stereochemistry of the reported 7*α*-hydroxytheonellasterol has been revised based on the outcome of a series of chemical modifications and the X-ray crystallography data of **6**. The stereo and regiochemistry of the 1,2-epoxyalcohol moiety in 8*α*-hydroxy-14,15-*β*-epoxy-theonellasterol (**5**) was determined by GIAO chemical shift calculations. The reaction pathway for the 1,3-hydroxyl migration of **4** was calculated using quantum mechanical calculations to explain the observed reaction spontaneity. In addition, the unusual downfield shifts observed for H-5 in compounds **4** and **5** were rationalized through geometry optimizations, which indicated the presence of an 8*α*-hydroxyl group in 6/6/6/5-fused cyclic sterols.

## Figures and Tables

**Table 1:**
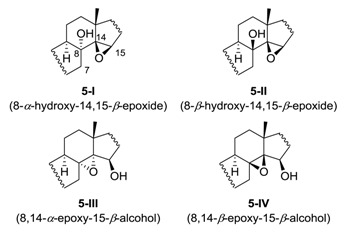
Comparison of experimental ^13^C chemical shifts in C_6_D_6_ with calculated ^13^C shifts for isomers **I**–**IV**.

Position	*δ*_exp_ (ppm)	*δ*_calcd_ (ppm)			
		5-I	5-II	5-III	5-IV
C-7	28.3	31.7	35.5	31.8	30.9
C-8	71.3	72.4	74.2	63.4	65.0
C-9	55.3	51.6	55.1	47.2	50.0
C-13	42.5	45.7	47.3	43.3	42.5
C-14	75.9	76.4	75.1	80.0	72.0
C-15	58.8	60.3	62.5	73.9	65.8
C-16	32.3	25.7	27.6	38.7	38.8
DP4+		100%	0%	0%	0%
*R* ^2^		0.9908	0.9828	0.9772	0.9845
MAD ^a^		1.38	1.91	2.30	1.64

^a^ Mean absolute deviation.

## Supplementary Materials

The following are available online at https://www.mdpi.com/1660-3397/18/12/607/s1, Supporting Information I; I. Experimental procedure, II. Computational methods, III. Determination of the C-24 configuration, IV. X-ray crystallography data; Supporting Information II; Figures SII-1–SII-57: ^1^H NMR, ^13^C NMR, COSY, HSQC, HMBC, NOESY spectra of **1**–**10**; CIF file for **6**.
