# Supplementary material for: Oxygenated Theonellastrols: Interpretation of Unusual Chemical Behaviors Using Quantum Mechanical Calculations and Stereochemical Reassignment of 7α-Hydroxytheonellasterol
Source: Mar Drugs. 2020 Nov 30;18(12):607. doi: 10.3390/md18120607 (PMC7760259; doi:10.3390/md18120607)
Supplement: Supplementary file 1 [file marinedrugs-18-00607-s001.zip › SI_revision/SI_NMR_revision.pdf]

Supporting Information II

Spectral data

Interpretation of Unusual Chemical Behaviors of Oxygenated theonellastrols using Quantum Mechanical Calculations and Stereochemical reassignment of 7α-hydroxytheonellasterol

A-Young Shin,[1,2] *Hyi-Seung Lee,* [1,2] *Yeon-Ju Lee,* [1,2] *Jong Seok Lee,* [1,2] *Arang Son,*[3] *Changhoon Cho,*[3] *and Jihoon Lee**[1,2]

[1]Korea Institute of Ocean Science and Technology (KIOST), Busan 49111, Republic of Korea

[2]Department of Marine Biotechnology, University of Science and Technology (UST), Daejeon 34113, Republic of Korea

[3]Department of Radiation Oncology, Samsung Medical Center, Seoul 06351, Republic of Korea

Table of contents

Figure SII-1. ^{1}H NMR spectrum of compound **1** (C_6D_6, 600 MHz). 4
Figure SII-2. ^{13}C NMR spectrum of compound **1** (C_6D_6, 150 MHz). 4
Figure SII-3. HSQC spectrum of compound **1** (C_6D_6, 600 MHz). 5
Figure SII-4. ^{1}H-^{1}H COSY spectrum of compound **1** (C_6D_6, 600 MHz). 5
Figure SII-5. HMBC spectrum of compound **1** (C_6D_6, 600 MHz). 6
Figure SII-6. NOESY spectrum of compound **1** (C_6D_6, 600 MHz). 6
Figure SII-7. HRMS spectrum of compound **1**. 7
Figure SII-8. ^{1}H NMR spectrum of compound **2** (C_6D_6, 600 MHz). 8
Figure SII-9. ^{13}C NMR spectrum of compound **2** (C_6D_6, 150 MHz). 8
Figure SII-10. HSQC spectrum of compound **2** (C_6D_6, 600 MHz). 9
Figure SII-11. ^{1}H-^{1}H COSY spectrum of compound **2** (C_6D_6, 600 MHz). 9
Figure SII-12. HMBC spectrum of compound **2** (C_6D_6, 600 MHz). 10
Figure SII-13. NOESY spectrum of compound **2** (C_6D_6, 600 MHz). 10
Figure SII-14. HRMS spectrum of compound **2**. 11
Figure SII-15. ^{1}H NMR spectrum of compound **3** (C_6D_6, 600 MHz). 12
Figure SII-16. ^{13}C NMR spectrum of compound **3** (C_6D_6, 150 MHz). 12
Figure SII-17. HSQC spectrum of compound **3** (C_6D_6, 600 MHz). 13
Figure SII-18. ^{1}H-^{1}H COSY spectrum of compound **3** (C_6D_6, 600 MHz). 13
Figure SII-19. HMBC spectrum of compound **3** (C_6D_6, 600 MHz). 14
Figure SII-20. NOESY spectrum of compound **3** (C_6D_6, 600 MHz). 14
Figure SII-21. HRMS spectrum of compound **3**. 15
Figure SII-22. ^{1}H NMR spectrum of compound **4** (C_6D_6, 600 MHz). 16
Figure SII-23. ^{13}C NMR spectrum of compound **4** (C_6D_6, 150 MHz). 16
Figure SII-24. HSQC spectrum of compound **4** (C_6D_6, 600 MHz). 17
Figure SII-25. ^{1}H-^{1}H COSY spectrum of compound **4** (C_6D_6, 600 MHz). 17
Figure SII-26. HMBC spectrum of compound **4** (C_6D_6, 600 MHz). 18
Figure SII-27. NOESY spectrum of compound **4** (C_6D_6, 600 MHz). 18
Figure SII-28. ^{1}H NMR spectrum of compound **5** (C_6D_6, 600 MHz). 19
Figure SII-29. ^{13}C NMR spectrum of compound **5** (C_6D_6, 150 MHz). 19
Figure SII-30. HSQC spectrum of compound **5** (C_6D_6, 600 MHz). 20
Figure SII-31. ^{1}H-^{1}H COSY spectrum of compound **5** (C_6D_6, 600 MHz). 20
Figure SII-32. HMBC spectrum of compound **5** (C_6D_6, 600 MHz). 21
Figure SII-33. NOESY spectrum of compound **5** (C_6D_6, 600 MHz). 21
Figure SII-34. HRMS spectrum of compound **5**. 22
Figure SII-35. ^{1}H NMR spectrum of compound **6** ($CDCl_3$, 600 MHz). 23
Figure SII-36. ^{13}C NMR spectrum of compound **6** ($CDCl_3$, 150 MHz). 23

Figure SII-37. HSQC spectrum of compound **6** ($CDCl_3$, 600 MHz). 24
Figure SII-38. ^{1}H-^{1}H COSY spectrum of compound **6** ($CDCl_3$, 600 MHz). 24
Figure SII-39. HMBC spectrum of compound **6** ($CDCl_3$, 600 MHz). 25
Figure SII-40. NOESY spectrum of compound **6** ($CDCl_3$, 600 MHz). 25
Figure SII-41. HRMS spectrum of compound **6**. 26
Figure SII-42. ^{1}H NMR spectrum of compound **7** (C_6D_6, 600 MHz). 27
Figure SII-43. ^{13}C NMR spectrum of compound **7** (C_6D_6, 150 MHz). 27
Figure SII-44. HSQC spectrum of compound **7** (C_6D_6, 600 MHz). 28
Figure SII-45. ^{1}H-^{1}H COSY spectrum of compound **7** (C_6D_6, 600 MHz). 28
Figure SII-46. HMBC spectrum of compound **7** (C_6D_6, 600 MHz). 29
Figure SII-47. NOESY spectrum of compound **7** (C_6D_6, 600 MHz). 29
Figure SII-48. HRMS spectrum of compound **7**. 30
Figure SII-49. ^{1}H NMR spectrum of compound **8** ($CDCl_3$, 600 MHz). 31
Figure SII-50. ^{13}C NMR spectrum of compound **8** ($CDCl_3$, 150 MHz). 31
Figure SII-51. HSQC spectrum of compound **8** ($CDCl_3$, 600 MHz). 32
Figure SII-52. ^{1}H-^{1}H COSY spectrum of compound **8** ($CDCl_3$, 600 MHz). 32
Figure SII-53. HMBC spectrum of compound **8** ($CDCl_3$, 600 MHz). 33
Figure SII-54. NOESY spectrum of compound **8** ($CDCl_3$, 600 MHz). 33
Figure SII-55. HRMS spectrum of compound **8**. 34
Figure SII-56. ^{1}H NMR spectrum of compound **9** (C_6D_6, 600 MHz). 35
Figure SII-57. ^{13}C NMR spectrum of compound **9** (C_6D_6, 150 MHz). 35
Figure SII-58. HSQC spectrum of compound **9** (C_6D_6, 600 MHz). 36
Figure SII-59. ^{1}H-^{1}H COSY spectrum of compound **9** (C_6D_6, 600 MHz). 36
Figure SII-60. HMBC spectrum of compound **9** (C_6D_6, 600 MHz). 37
Figure SII-61. NOESY spectrum of compound **9** (C_6D_6, 600 MHz). 37
Figure SII-62. ^{1}H NMR spectrum of 3-OMe-swinhosterol C **10** (C_6D_6, 600 MHz). 38
Figure SII-63. ^{13}C NMR spectrum of 3-OMe-swinhosterol C **10** (C_6D_6, 150 MHz). 38
Figure SII-64. NOESY spectrum of 3-OMe-swinhosterol C **10** (C_6D_6, 600 MHz). 39
Figure SII-65. HRMS spectrum of compound **10.** 39

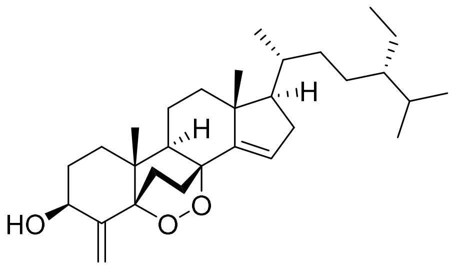

1

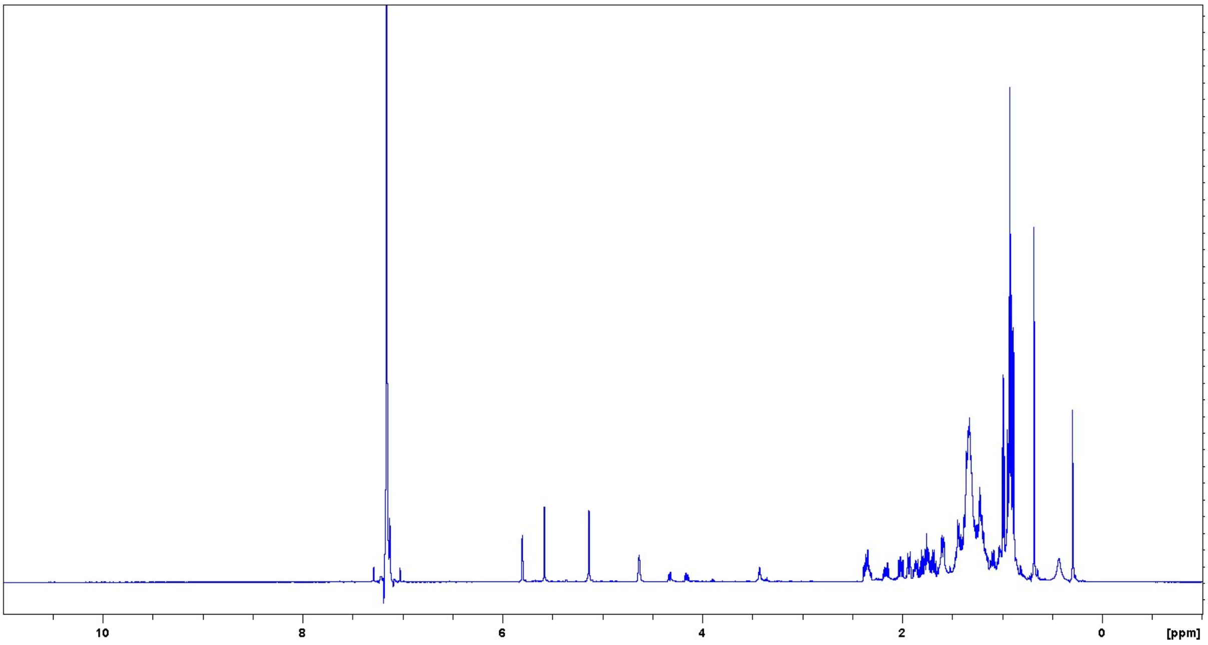

Figure SII-1. ^{1}H NMR spectrum of compound **1** (C_6D_6, 600 MHz).

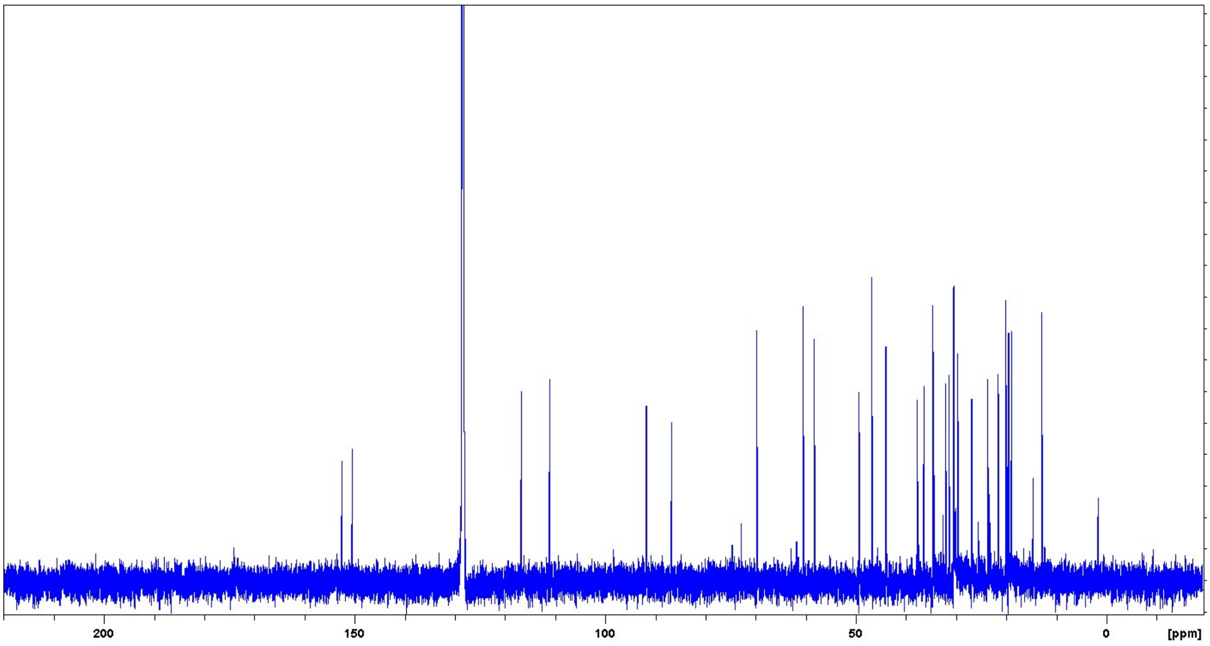

Figure SII-2. ^{13}C NMR spectrum of compound **1** (C_6D_6, 150 MHz).

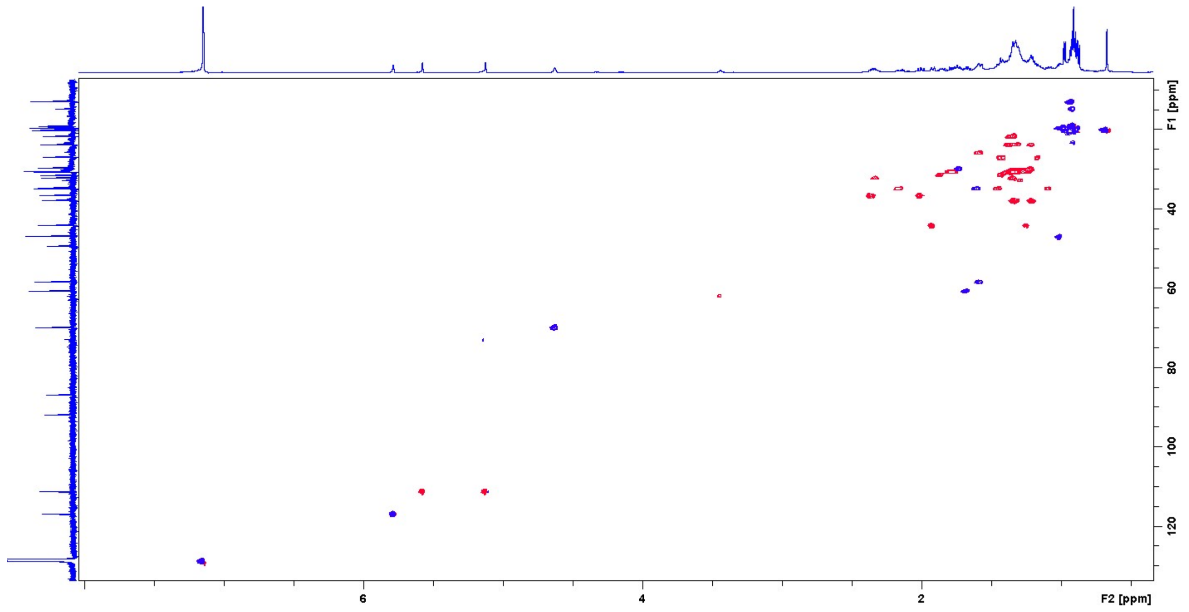

Figure SII-3. HSQC spectrum of compound **1** (C_6D_6, 600 MHz).

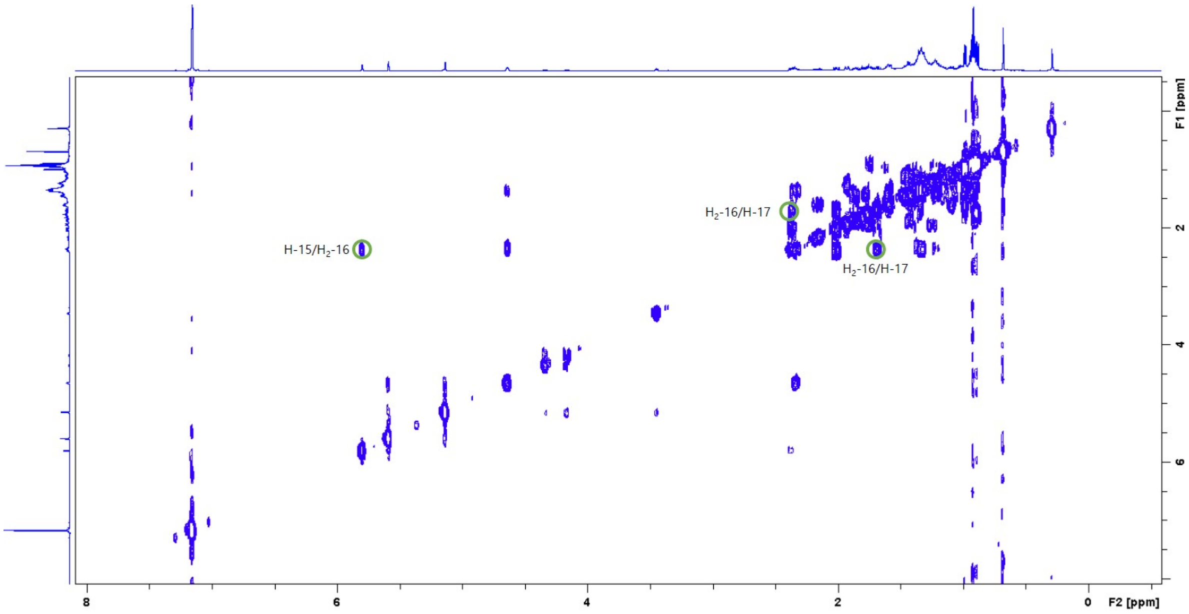

Figure SII-4. ^{1}H-^{1}H COSY spectrum of compound **1** (C_6D_6, 600 MHz).

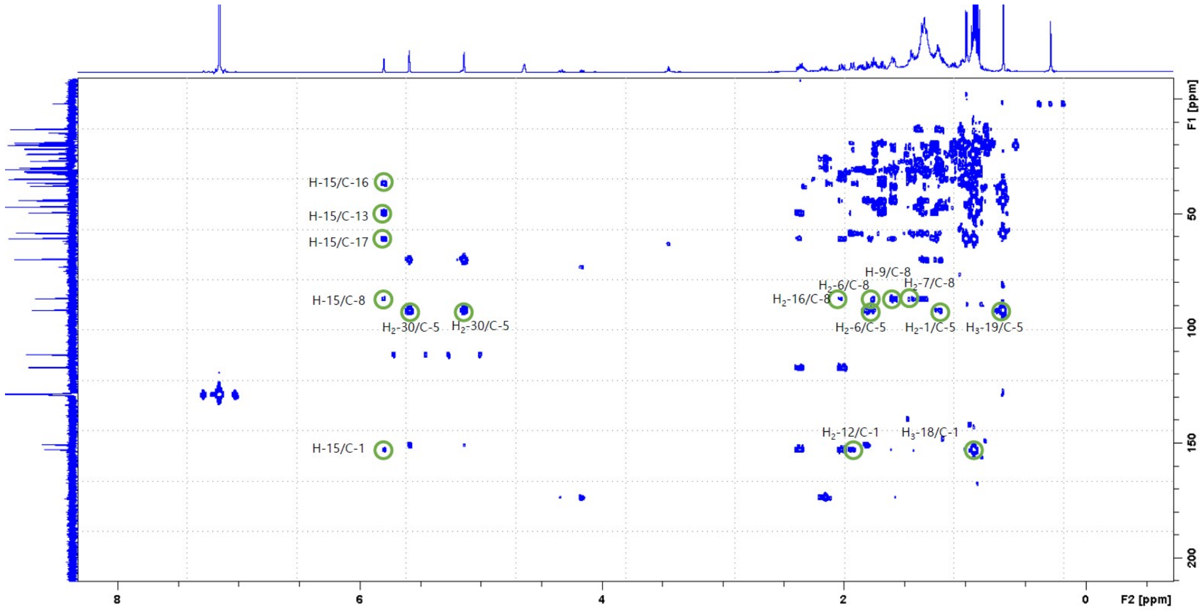

Figure SII-5. HMBC spectrum of compound **1** (C_6D_6, 600 MHz).

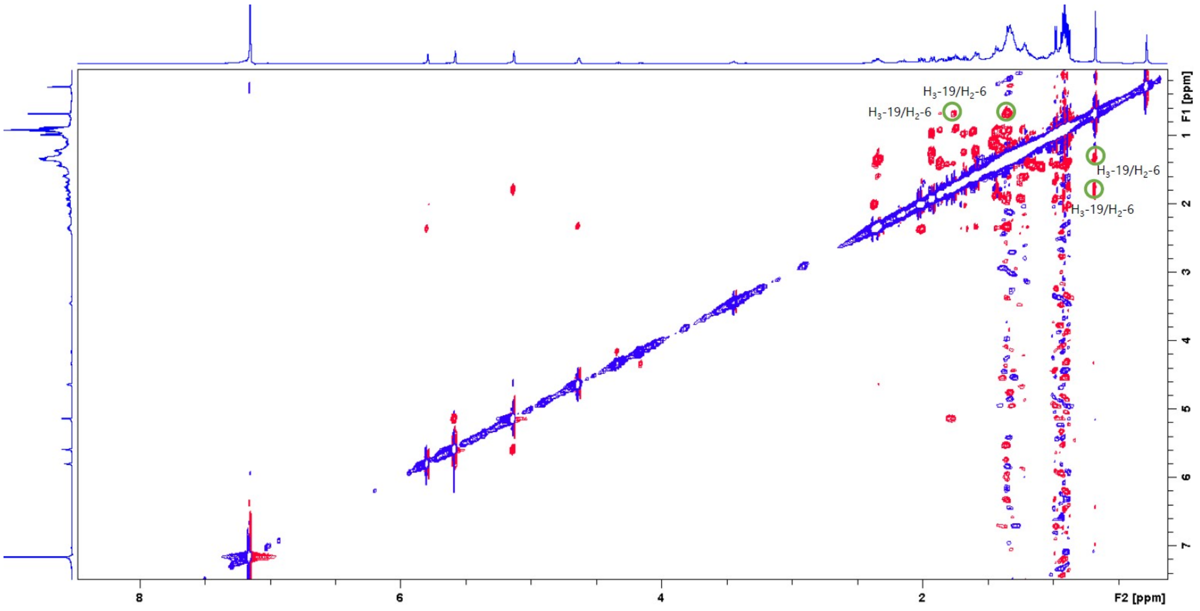

Figure SII-6. NOESY spectrum of compound **1** (C_6D_6, 600 MHz).

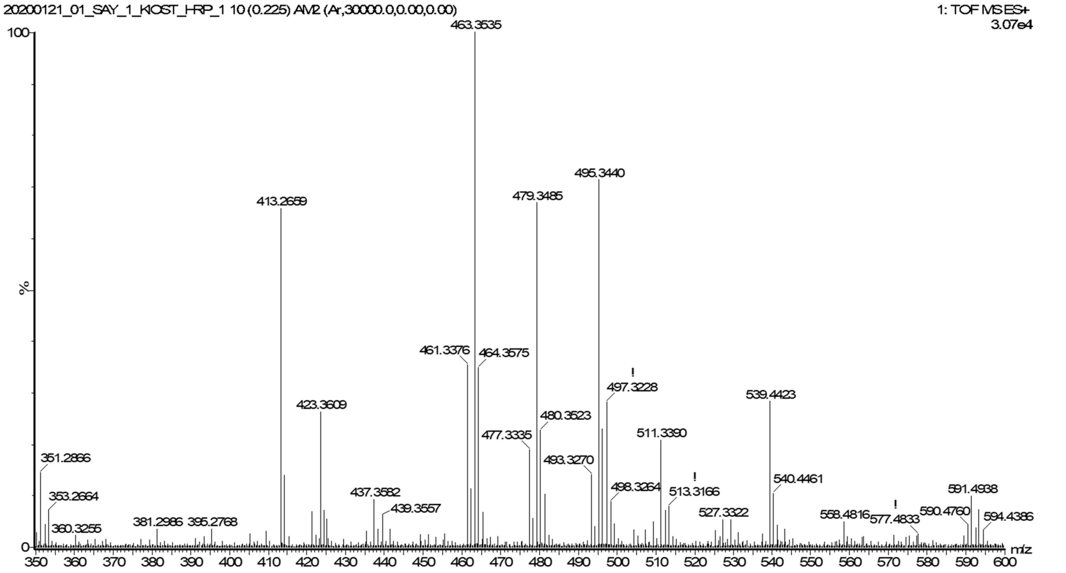

Figure SII-7. HRMS spectrum of compound **1**.

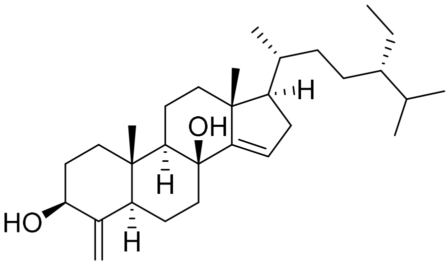

2

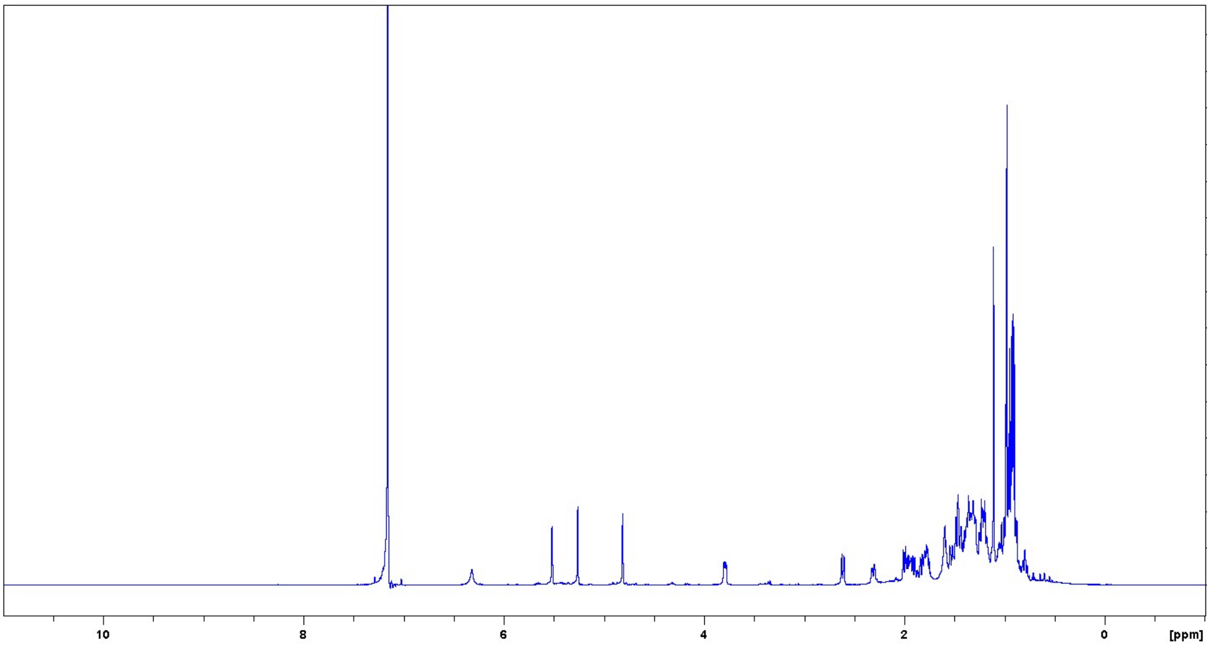

Figure SII-8. ^{1}H NMR spectrum of compound **2** (C_6D_6, 600 MHz).

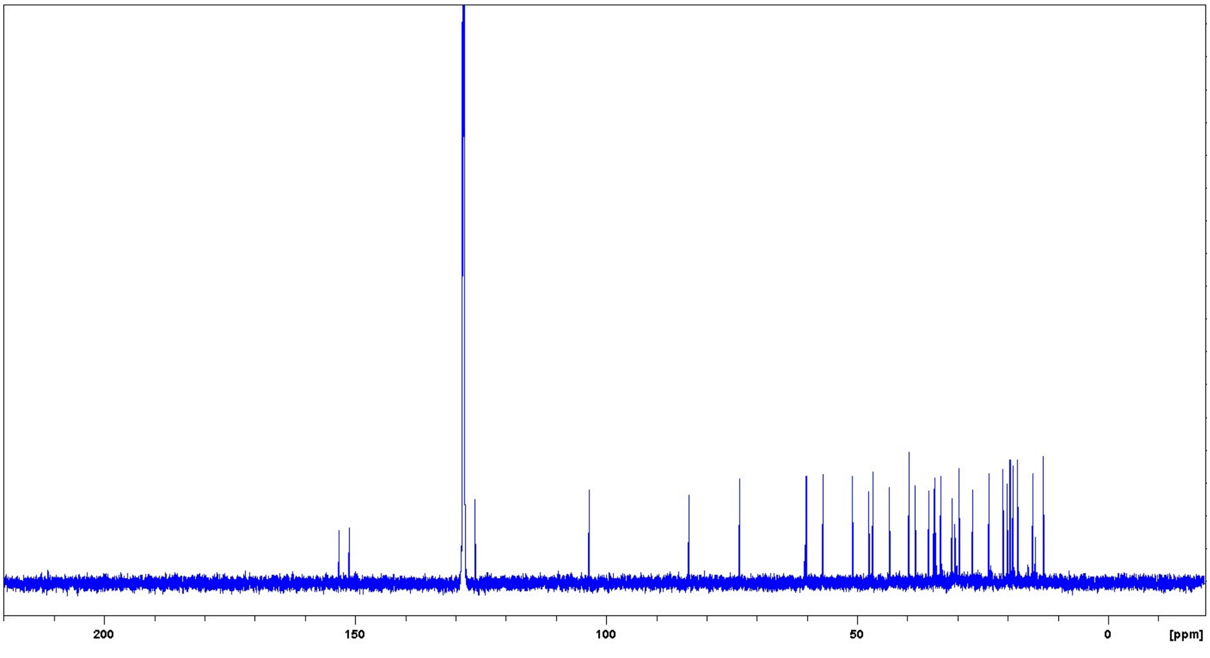

Figure SII-9. ^{13}C NMR spectrum of compound **2** (C_6D_6, 150 MHz).

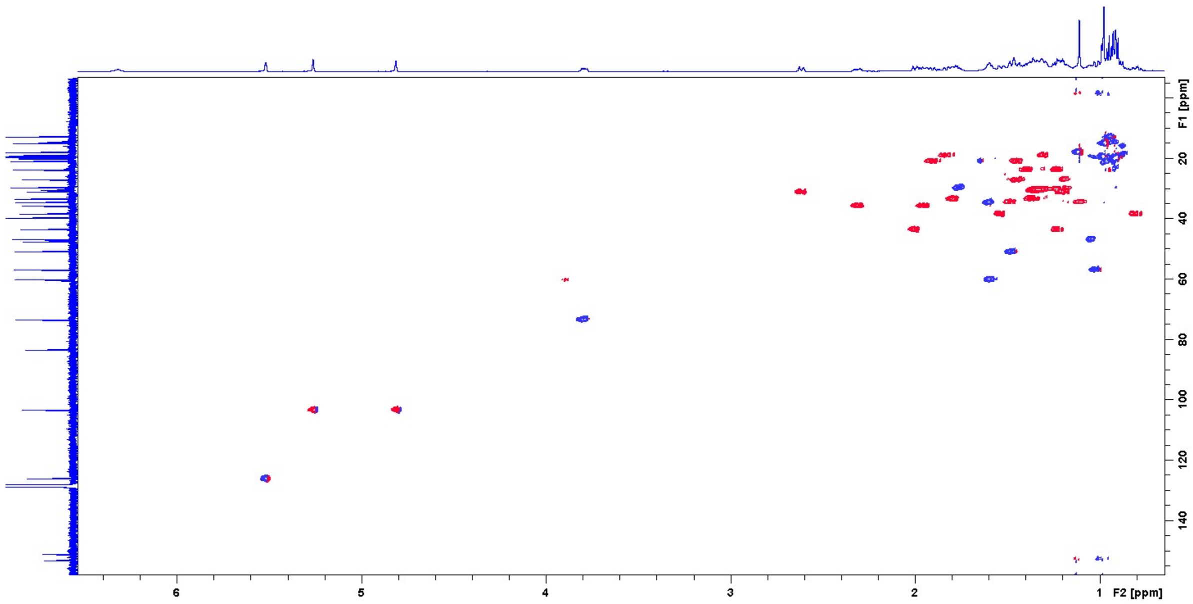

Figure SII-10. HSQC spectrum of compound **2** (C_6D_6, 600 MHz).

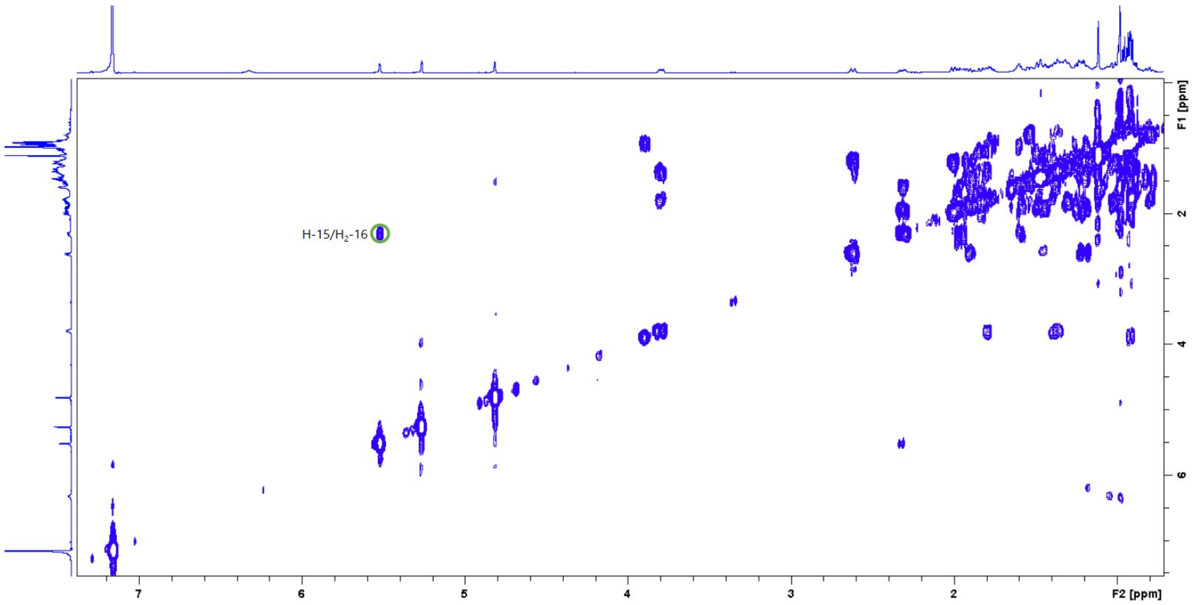

Figure SII-11. ^{1}H-^{1}H COSY spectrum of compound **2** (C_6D_6, 600 MHz).

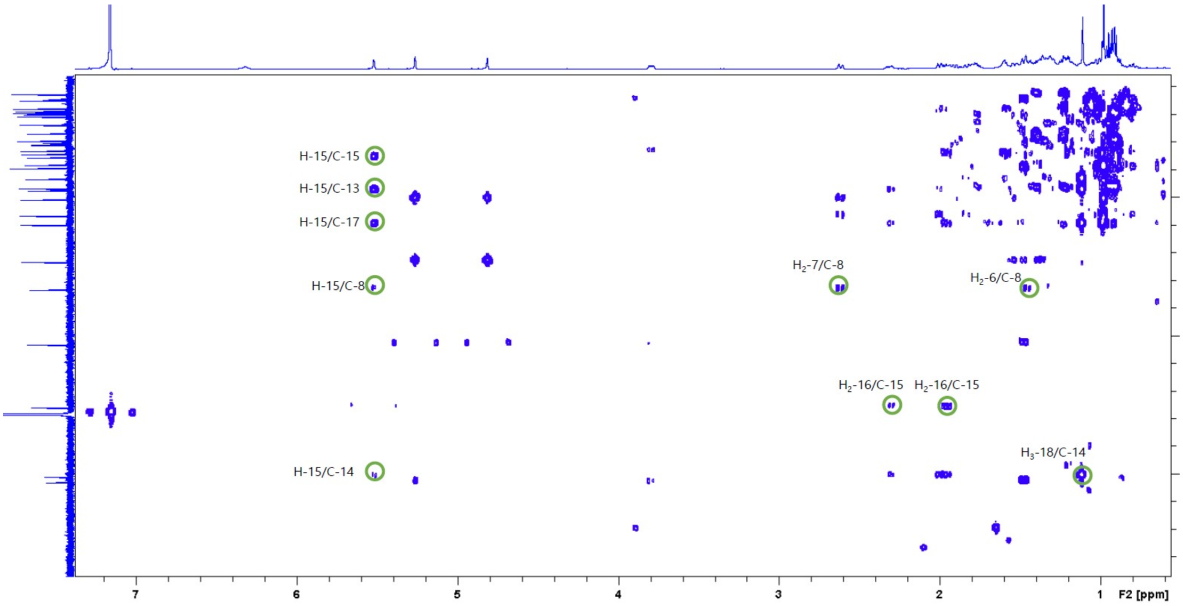

Figure SII-12. HMBC spectrum of compound **2** (C_6D_6, 600 MHz).

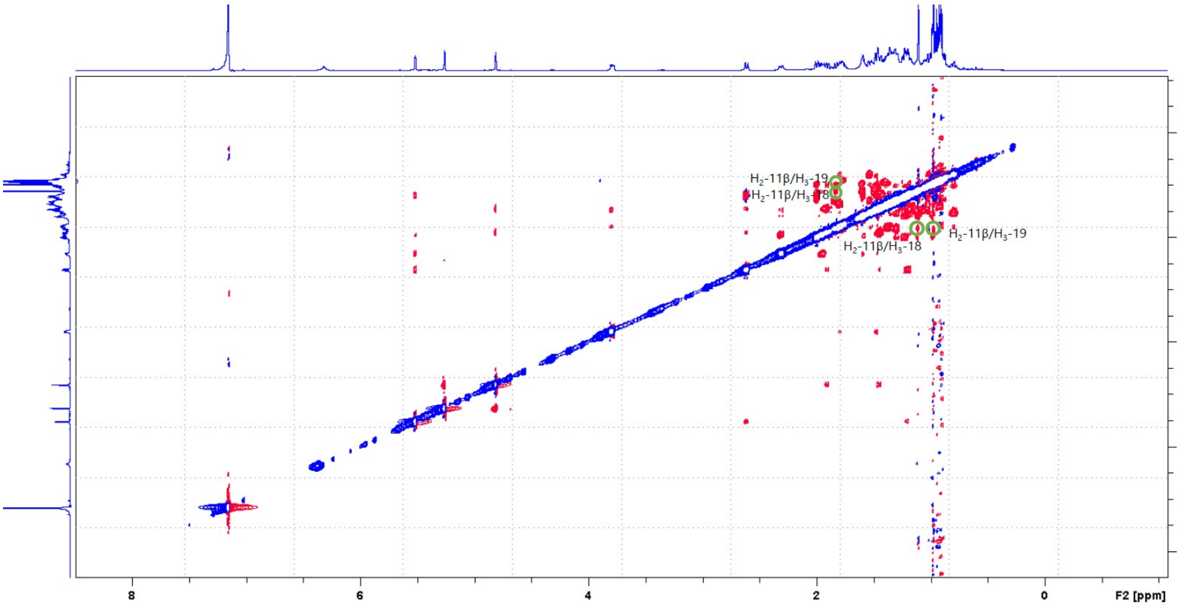

Figure SII-13. NOESY spectrum of compound **2** (C_6D_6, 600 MHz).

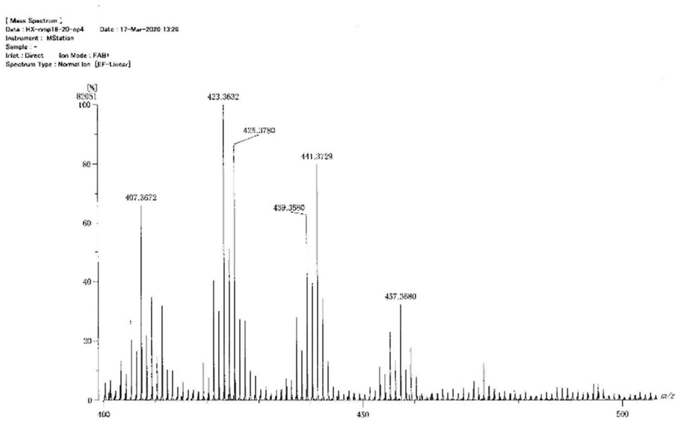

Figure SII-14. HRMS spectrum of compound **2**.

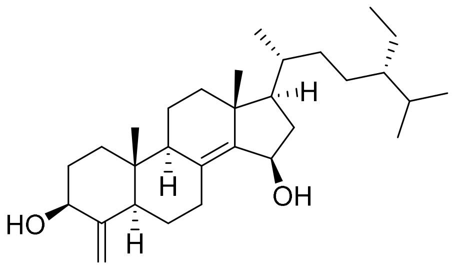

3

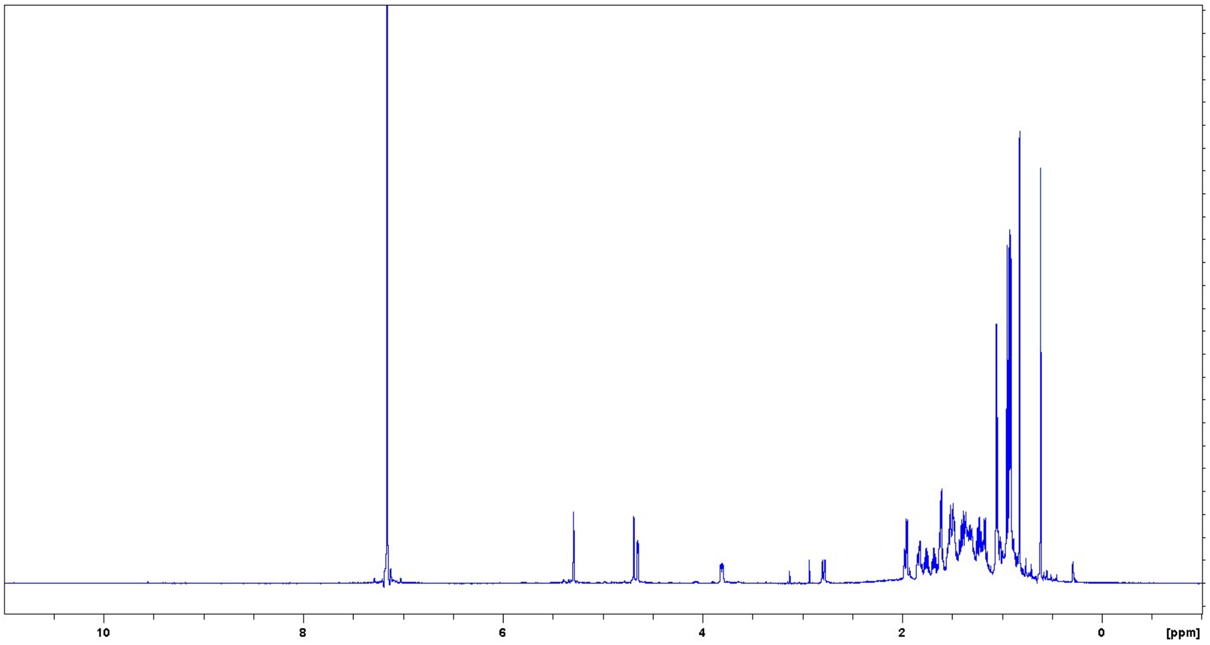

Figure SII-15. ^{1}H NMR spectrum of compound **3** (C_6D_6, 600 MHz).

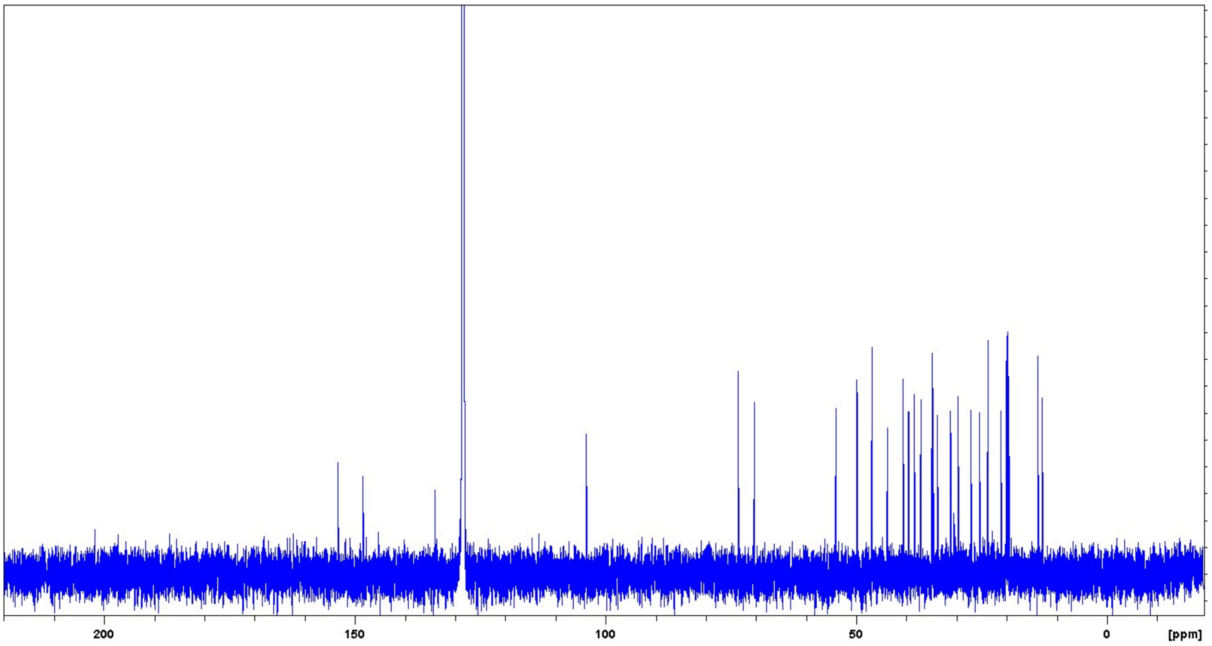

Figure SII-16. ^{13}C NMR spectrum of compound **3** (C_6D_6, 150 MHz).

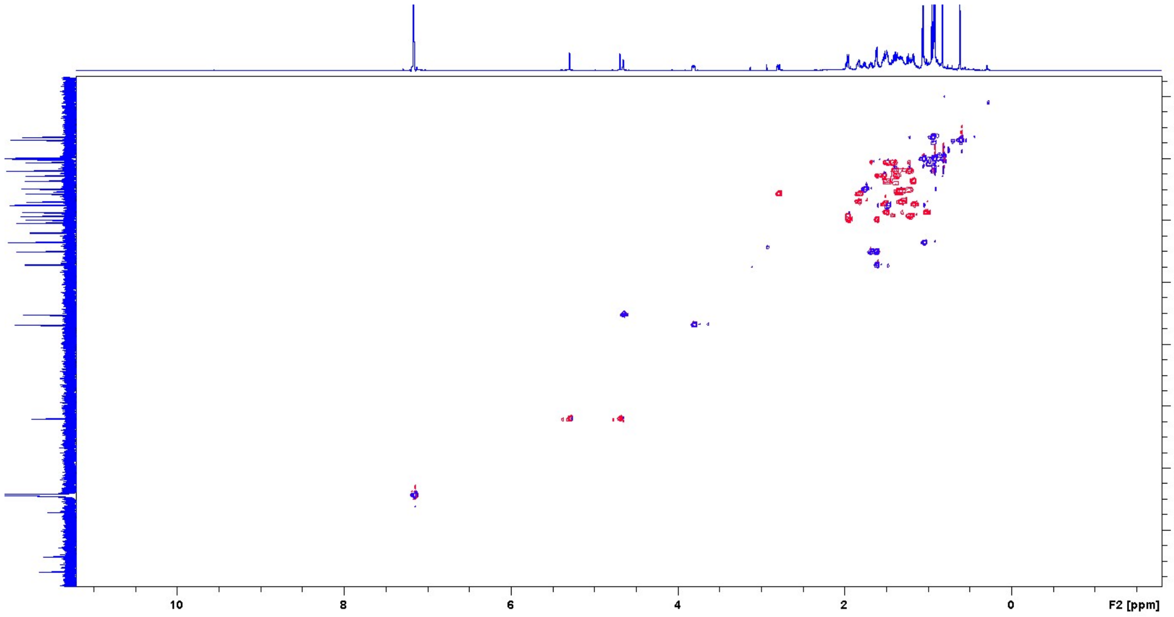

Figure SII-17. HSQC spectrum of compound **3** (C_6D_6, 600 MHz).

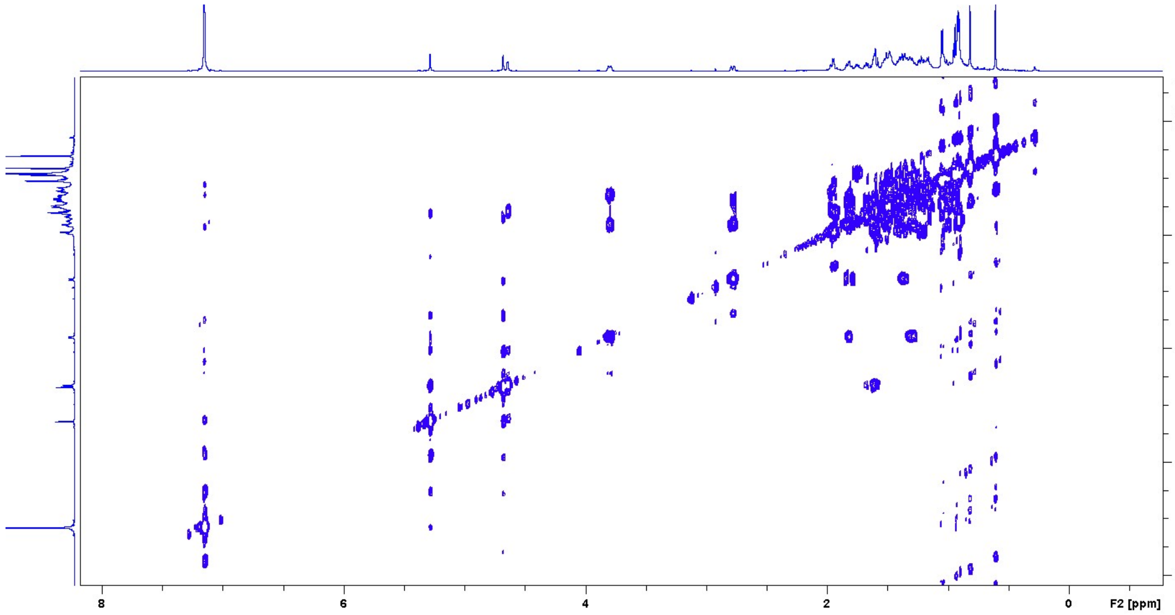

Figure SII-18. ^1H-^1H COSY spectrum of compound **3** (C_6D_6, 600 MHz).

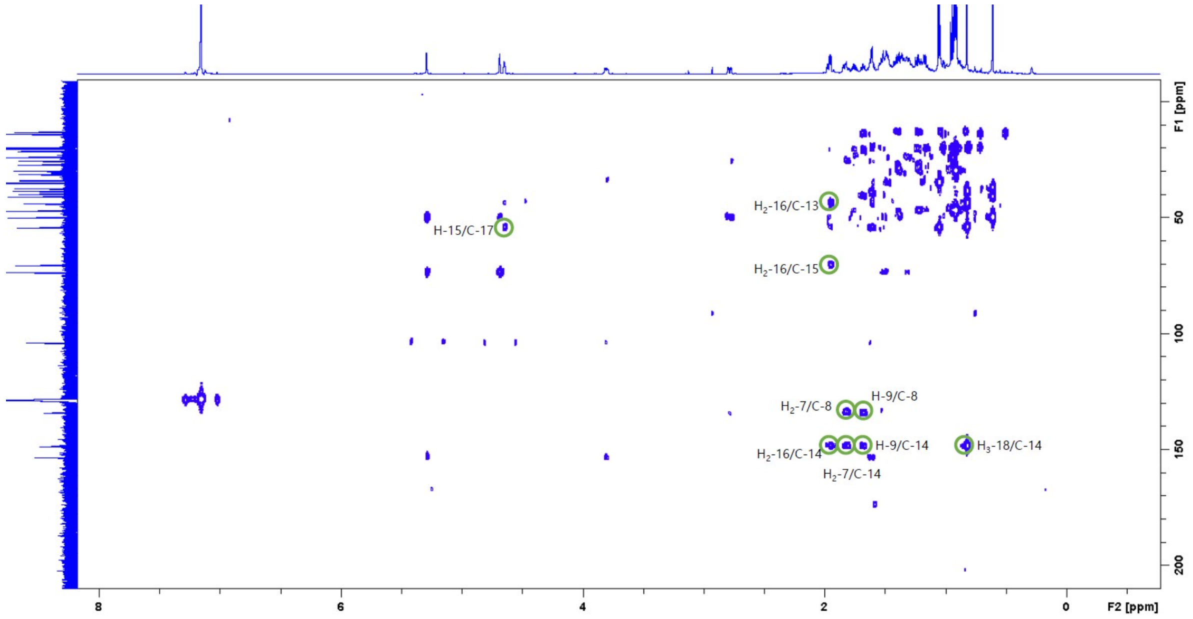

Figure SII-19. HMBC spectrum of compound **3** (C_6D_6, 600 MHz).

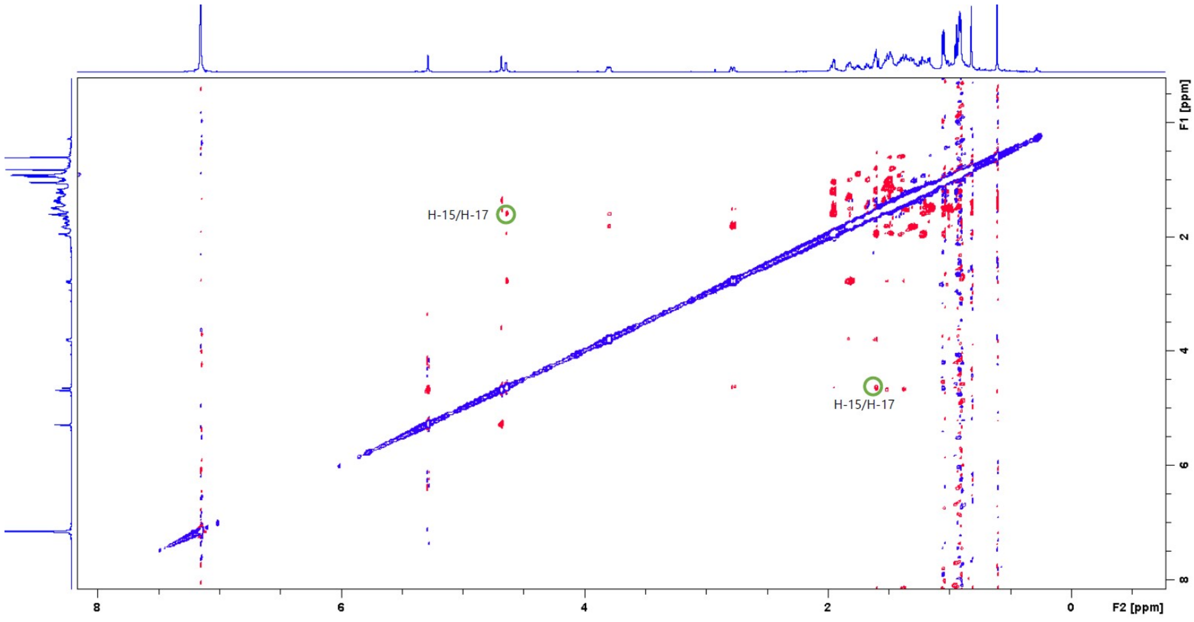

Figure SII-20. NOESY spectrum of compound **3** (C_6D_6, 600 MHz).

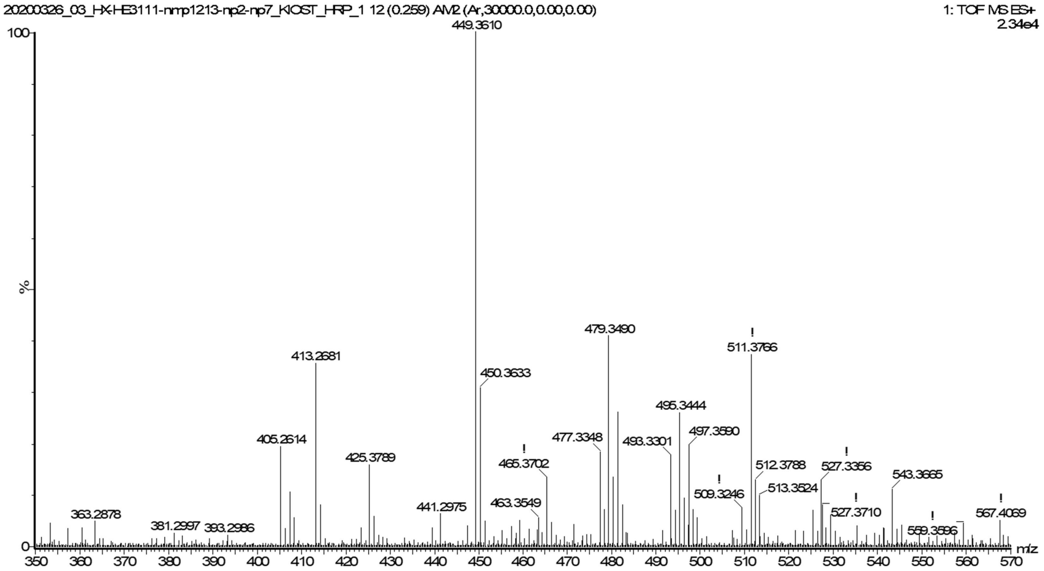

Figure SII-21. HRMS spectrum of compound **3**.

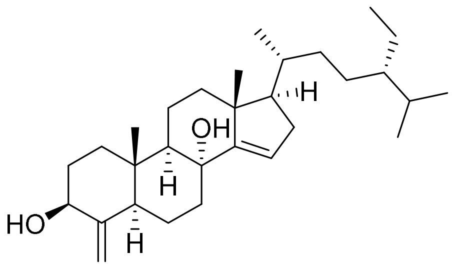

4

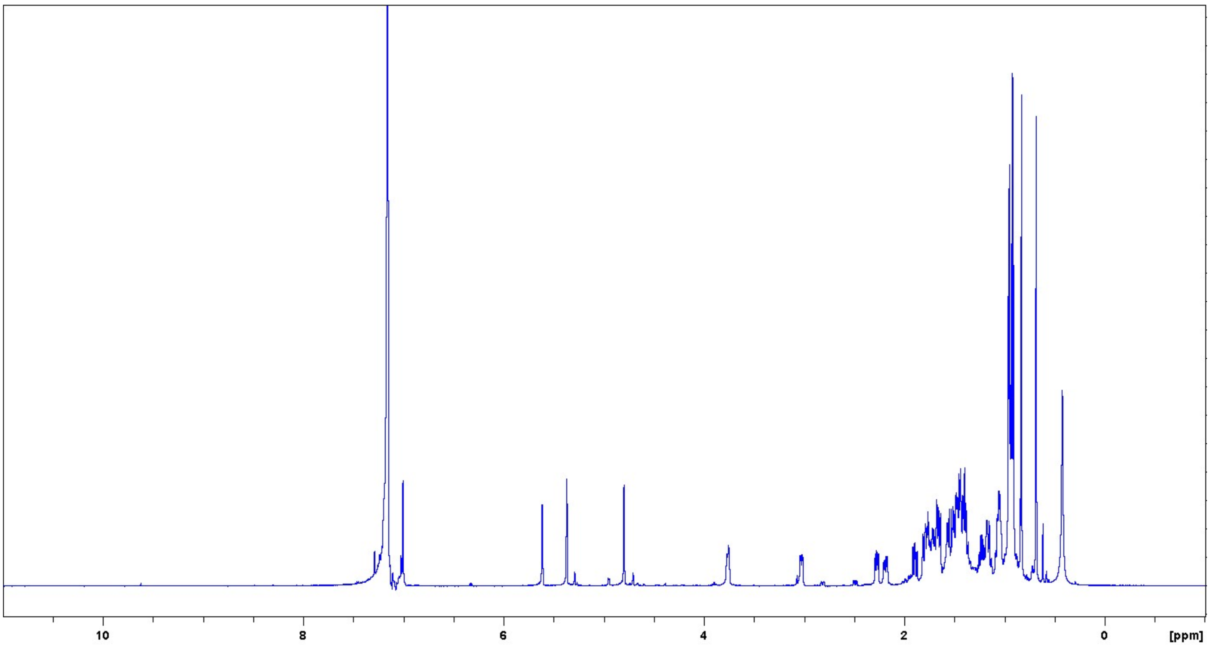

Figure SII-22. ^{1}H NMR spectrum of compound **4** (C_6D_6, 600 MHz).

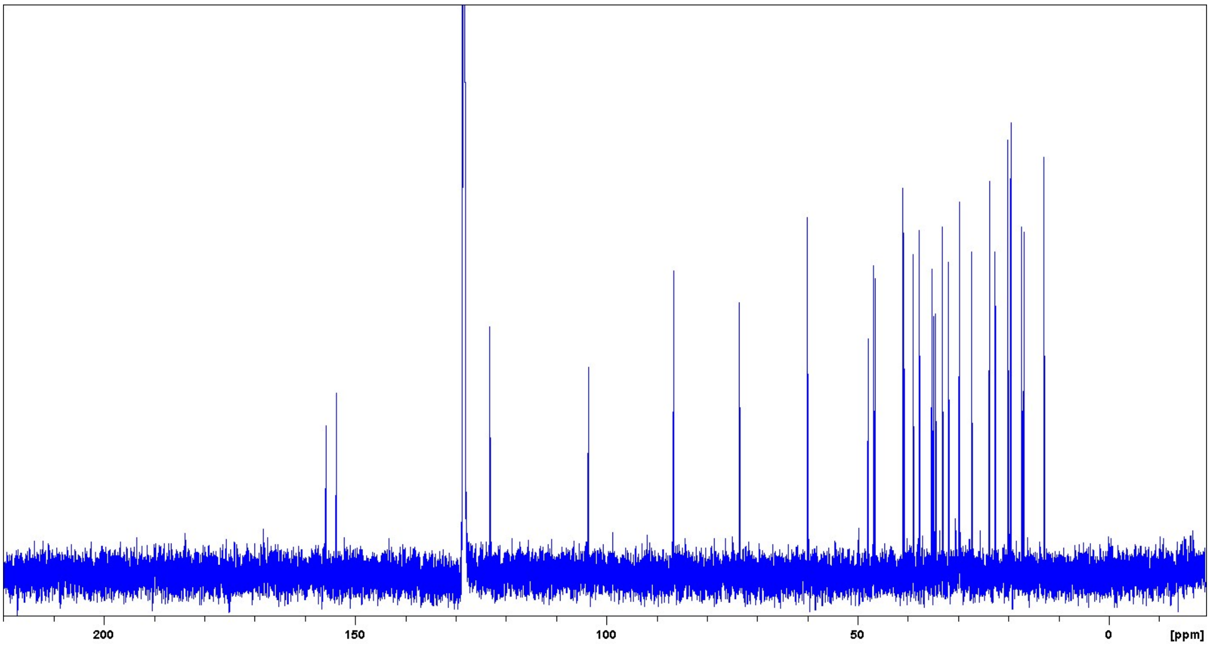

Figure SII-23. ^{13}C NMR spectrum of compound **4** (C_6D_6, 150 MHz).

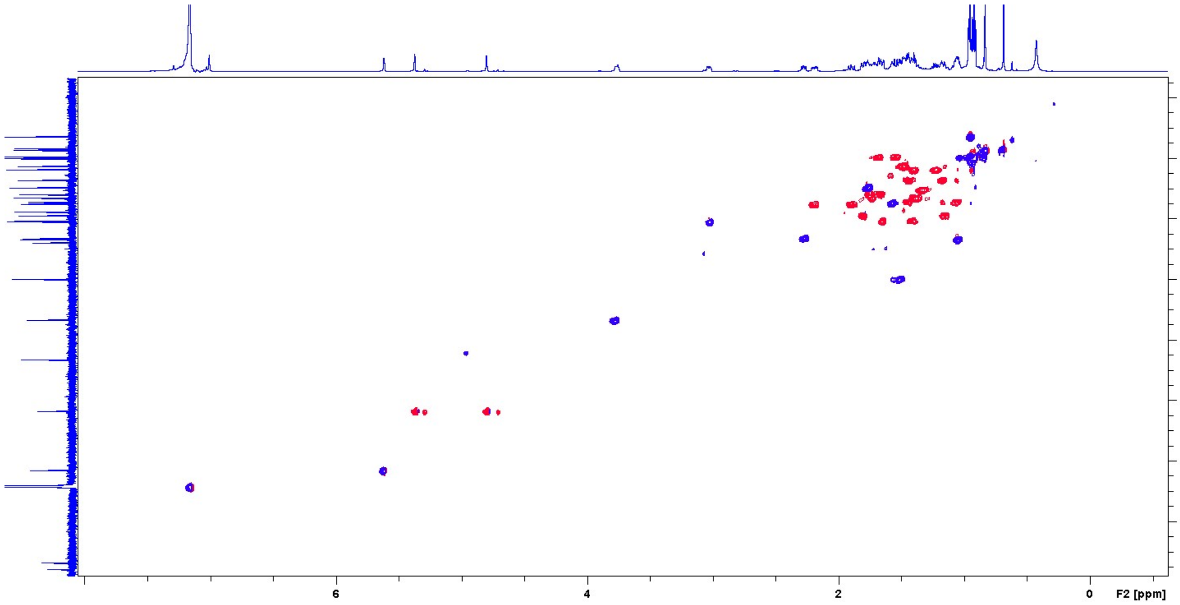

Figure SII-24. HSQC spectrum of compound **4** (C_6D_6, 600 MHz).

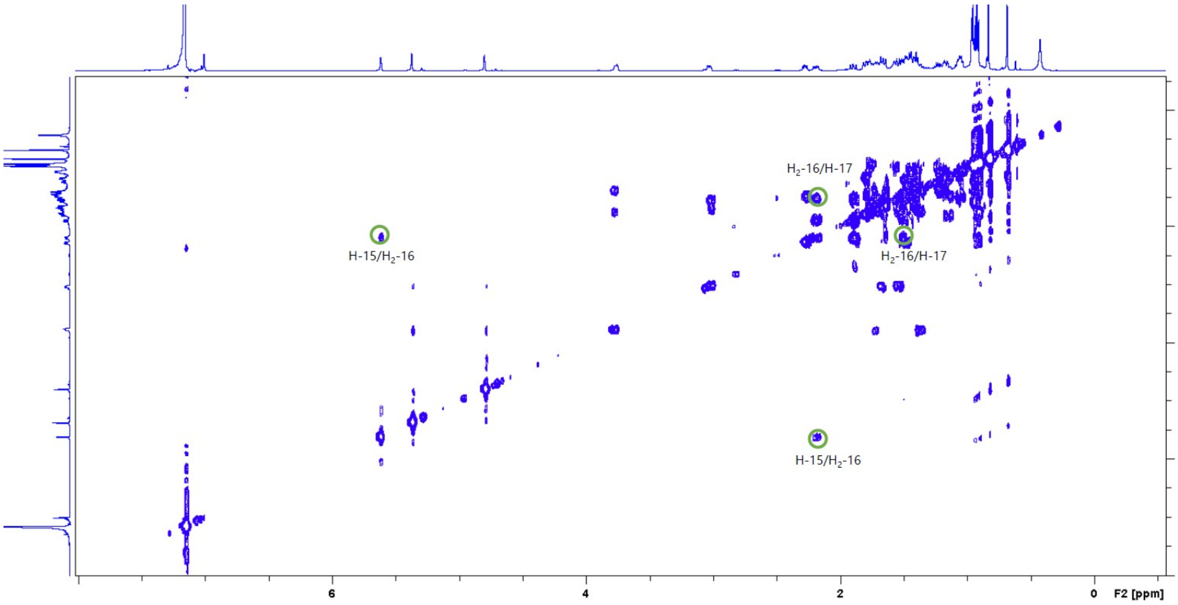

Figure SII-25. ^{1}H-^{1}H COSY spectrum of compound **4** (C_6D_6, 600 MHz).

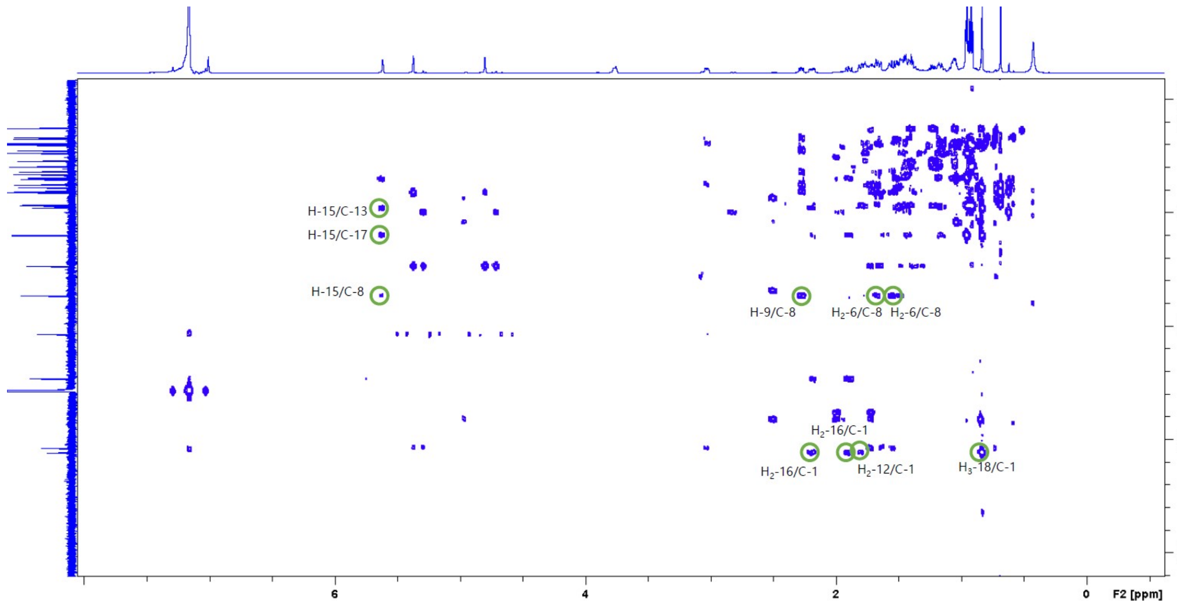

Figure SII-26. HMBC spectrum of compound **4** (C_6D_6, 600 MHz).

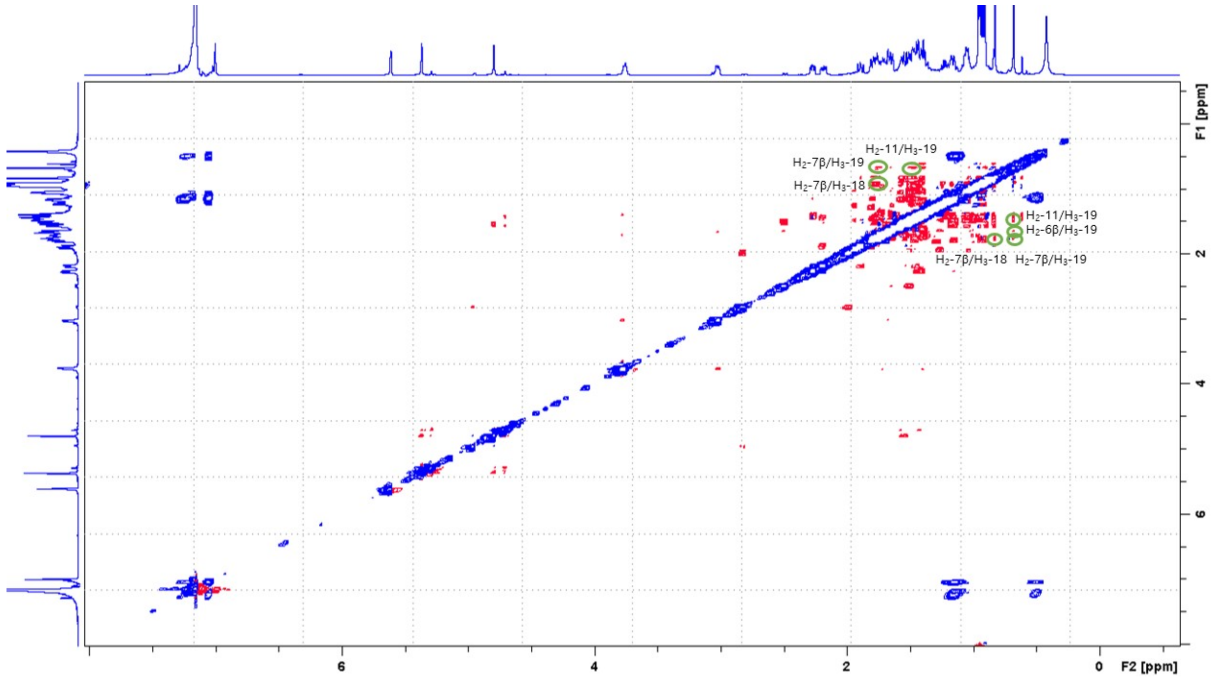

Figure SII-27. NOESY spectrum of compound **4** (C_6D_6, 600 MHz).

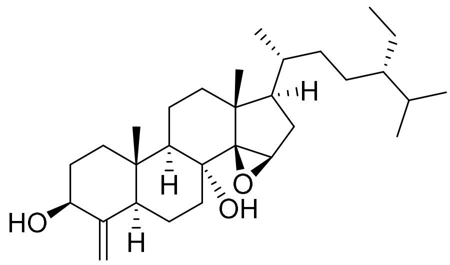

5

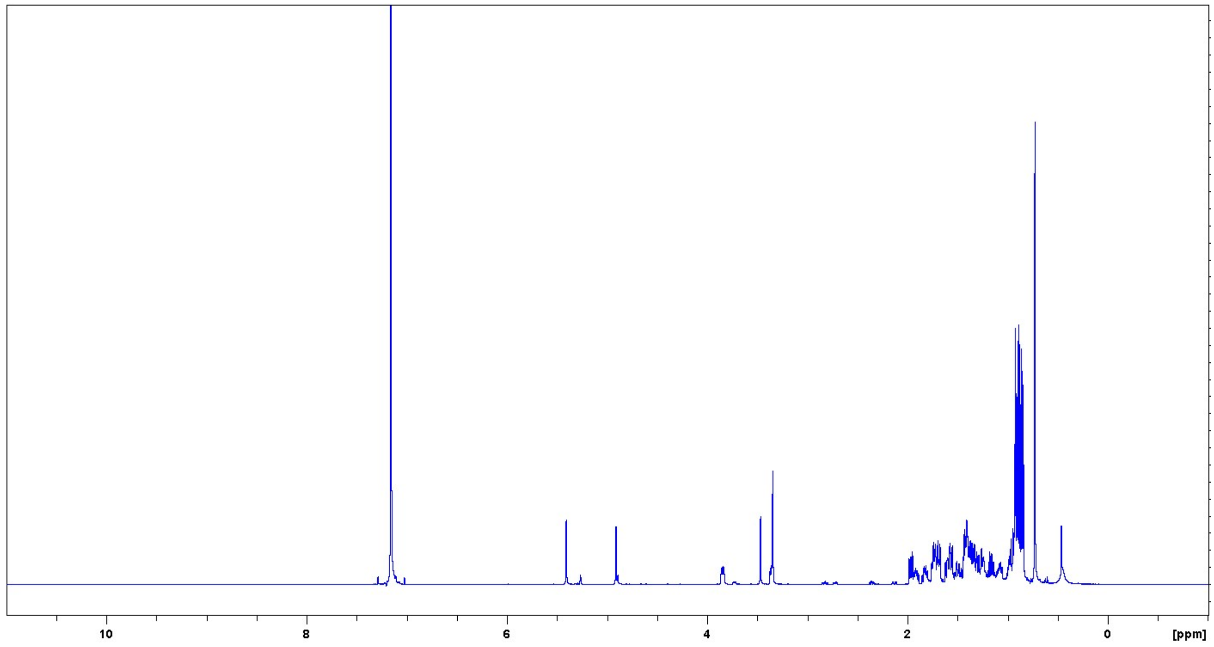

Figure SII-28. ^{1}H NMR spectrum of compound **5** (C_6D_6, 600 MHz).

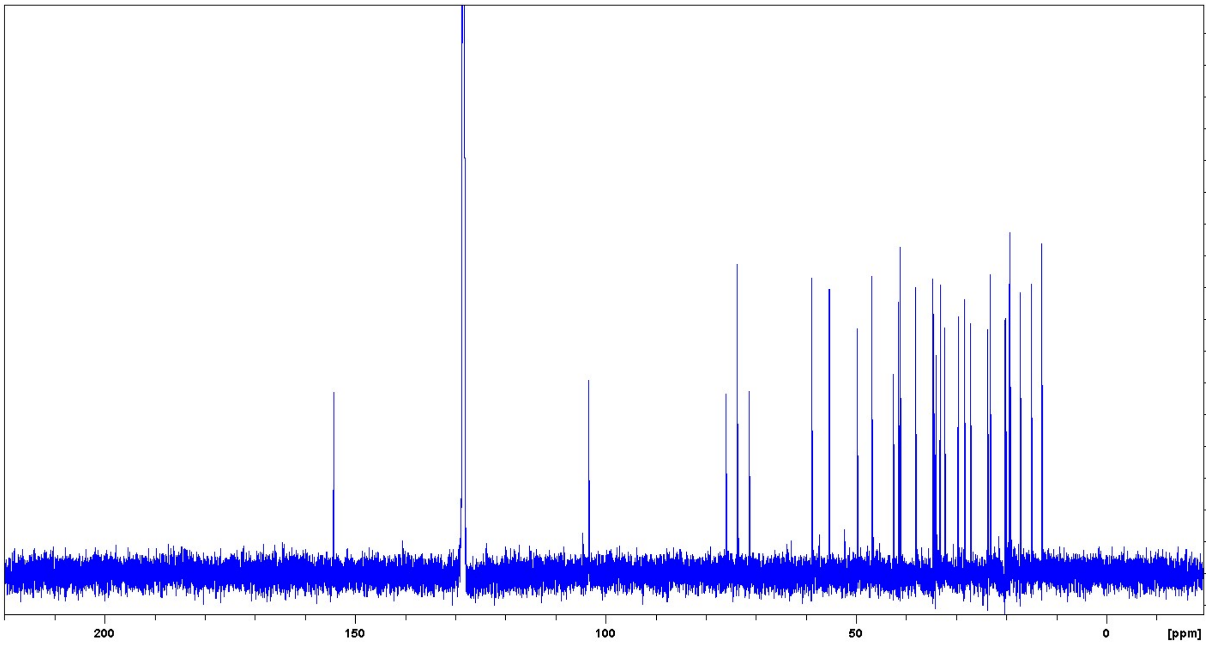

Figure SII-29. ^{13}C NMR spectrum of compound **5** (C_6D_6, 150 MHz).

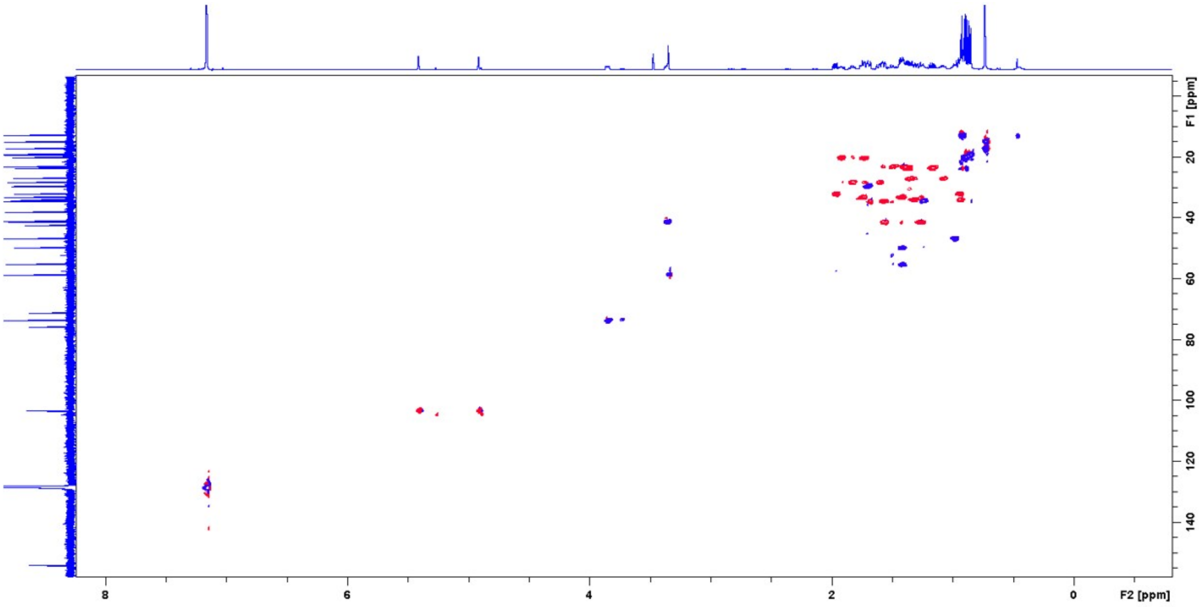

Figure SII-30. HSQC spectrum of compound **5** (C_6D_6, 600 MHz).

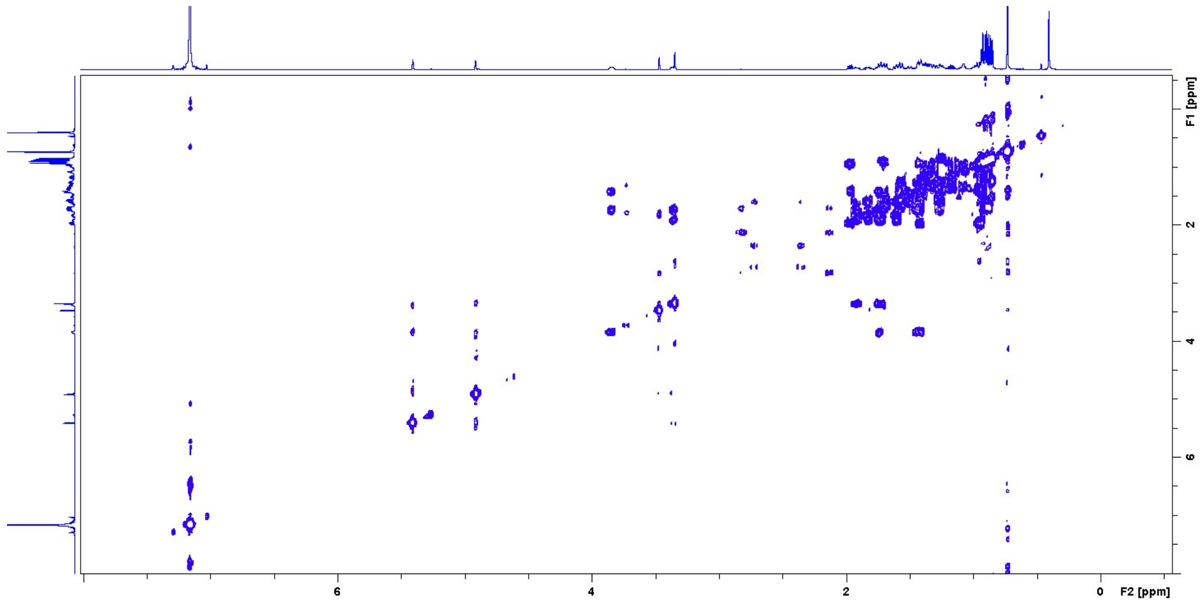

Figure SII-31. ^{1}H-^{1}H COSY spectrum of compound **5** (C_6D_6, 600 MHz).

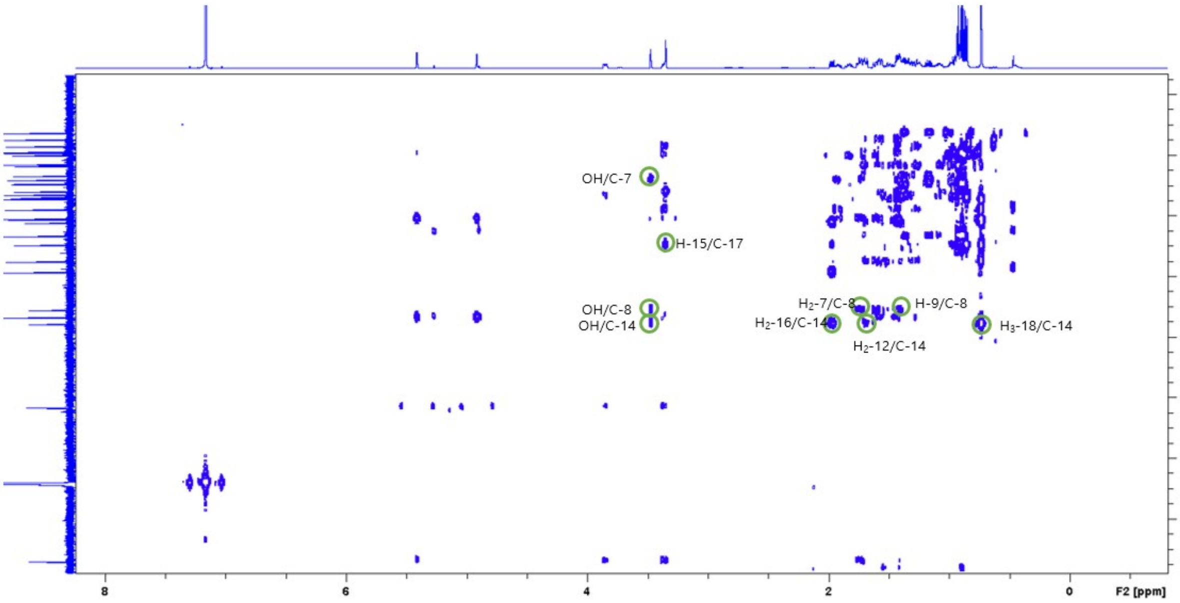

Figure SII-32. HMBC spectrum of compound **5** (C_6D_6, 600 MHz).

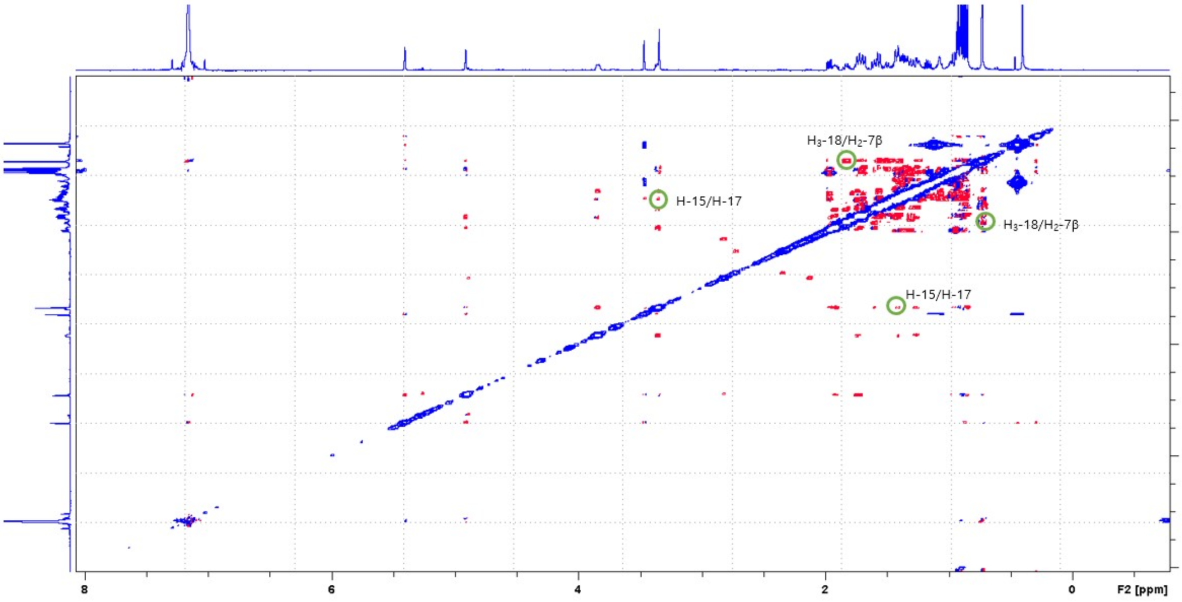

Figure SII-33. NOESY spectrum of compound **5** (C_6D_6, 600 MHz).

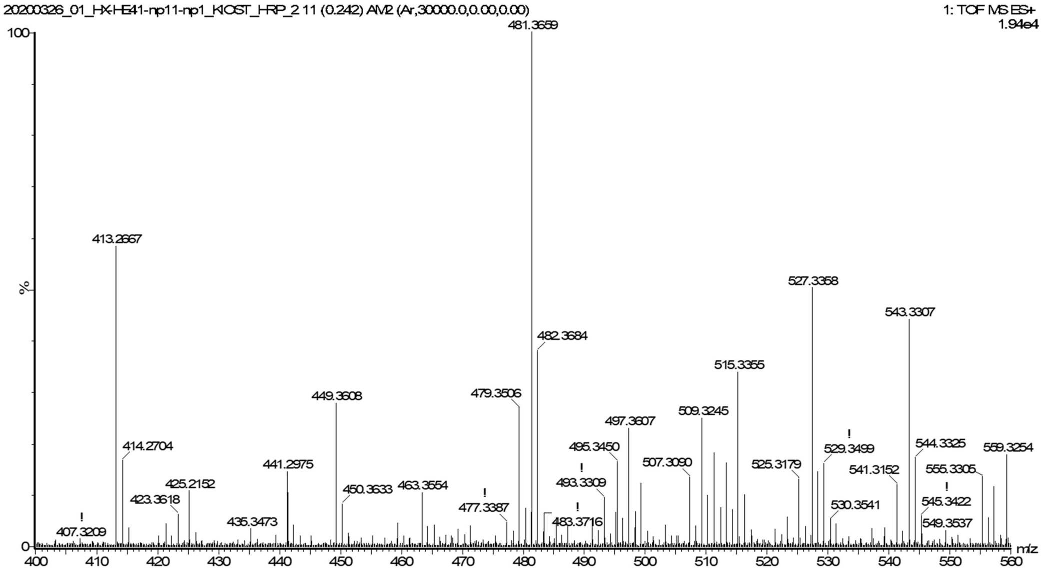

Figure SII-34. HRMS spectrum of compound **5**.

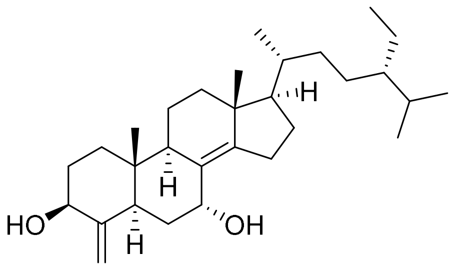

6

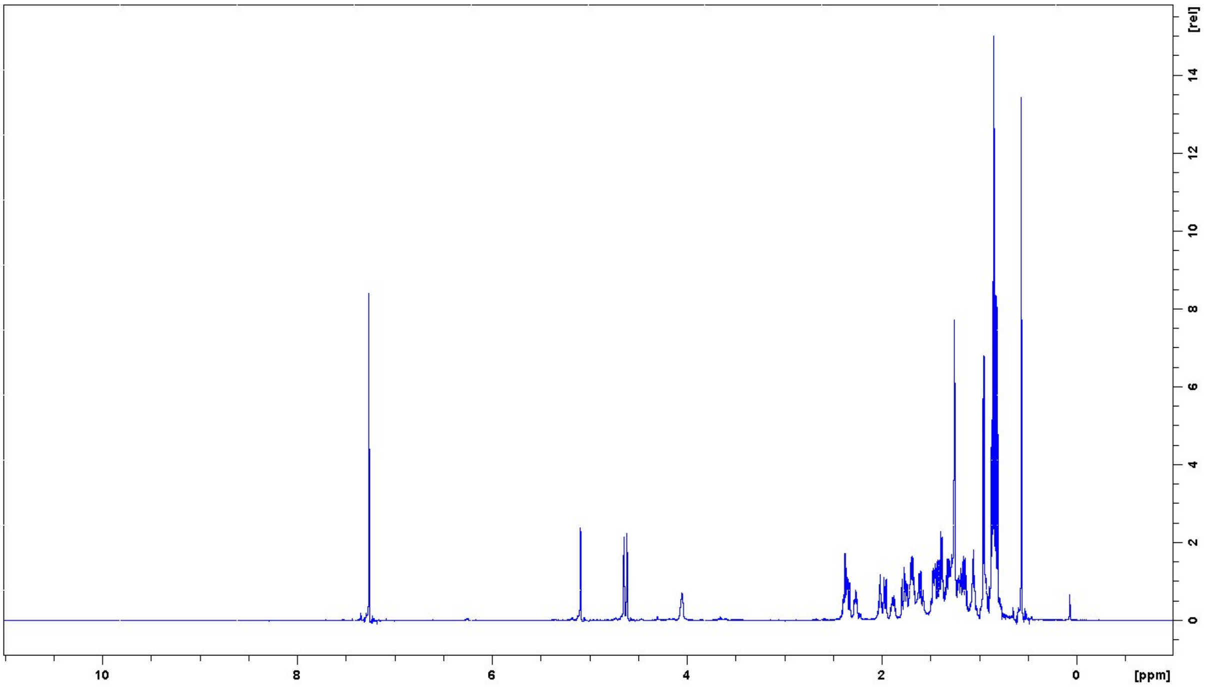

Figure SII-35. ^{1}H NMR spectrum of compound **6** ($CDCl_3$, 600 MHz).

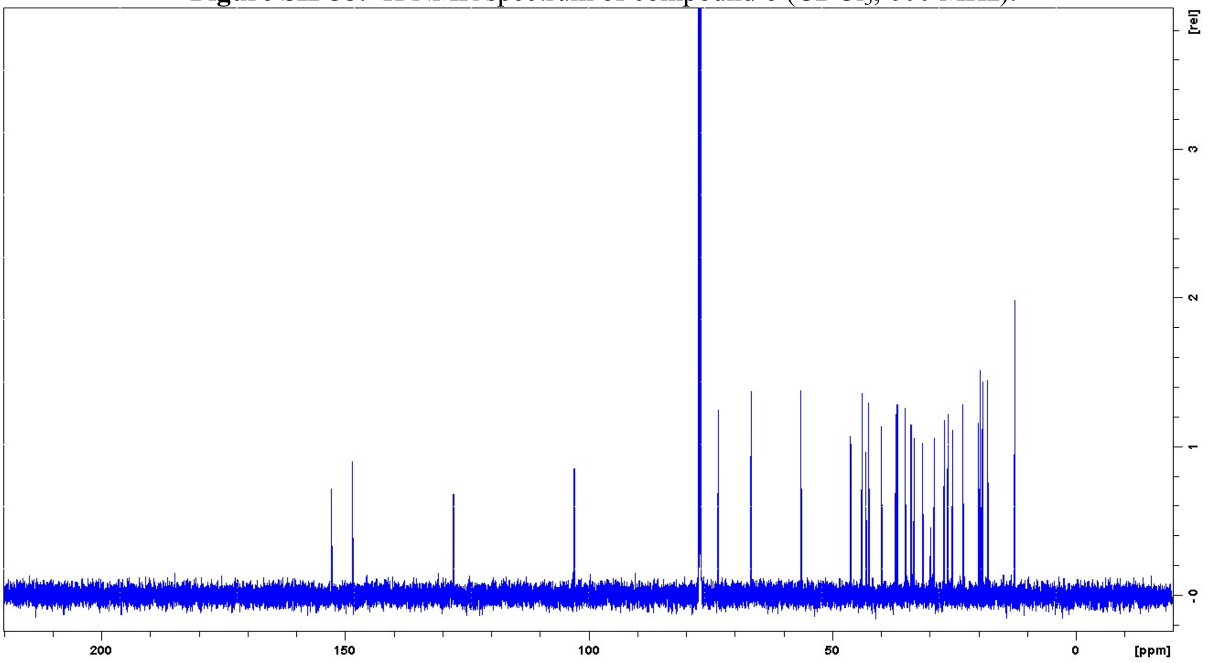

Figure SII-36. ^{13}C NMR spectrum of compound **6** ($CDCl_3$, 150 MHz).

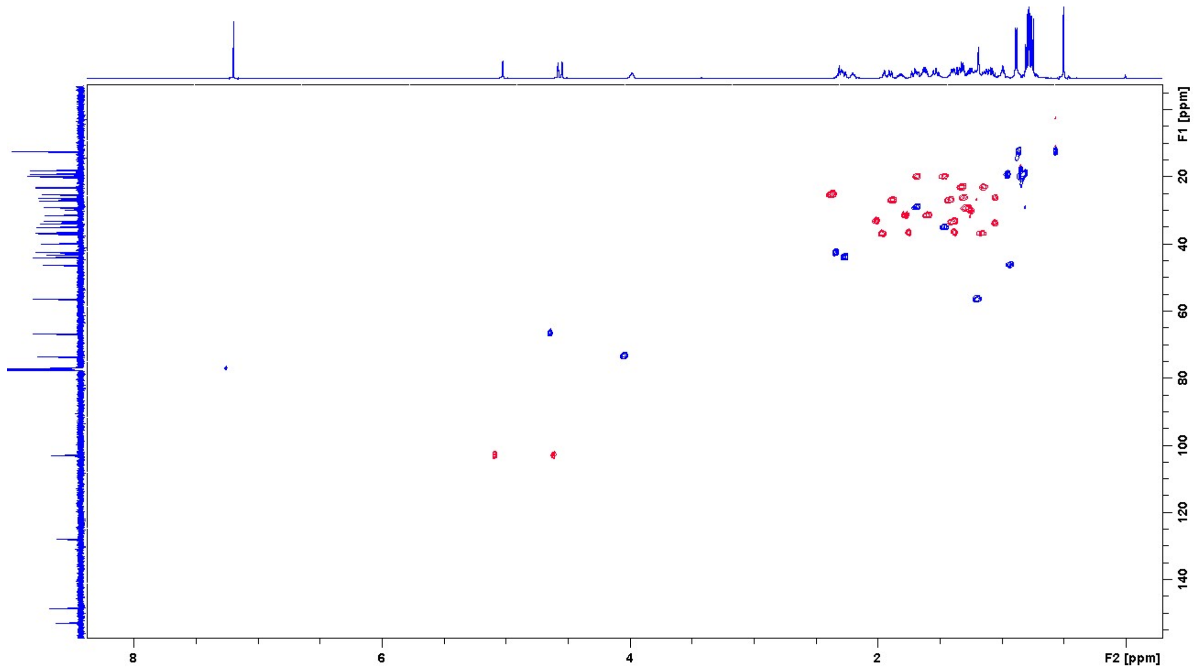

Figure SII-37. HSQC spectrum of compound **6** ($CDCl_3$, 600 MHz).

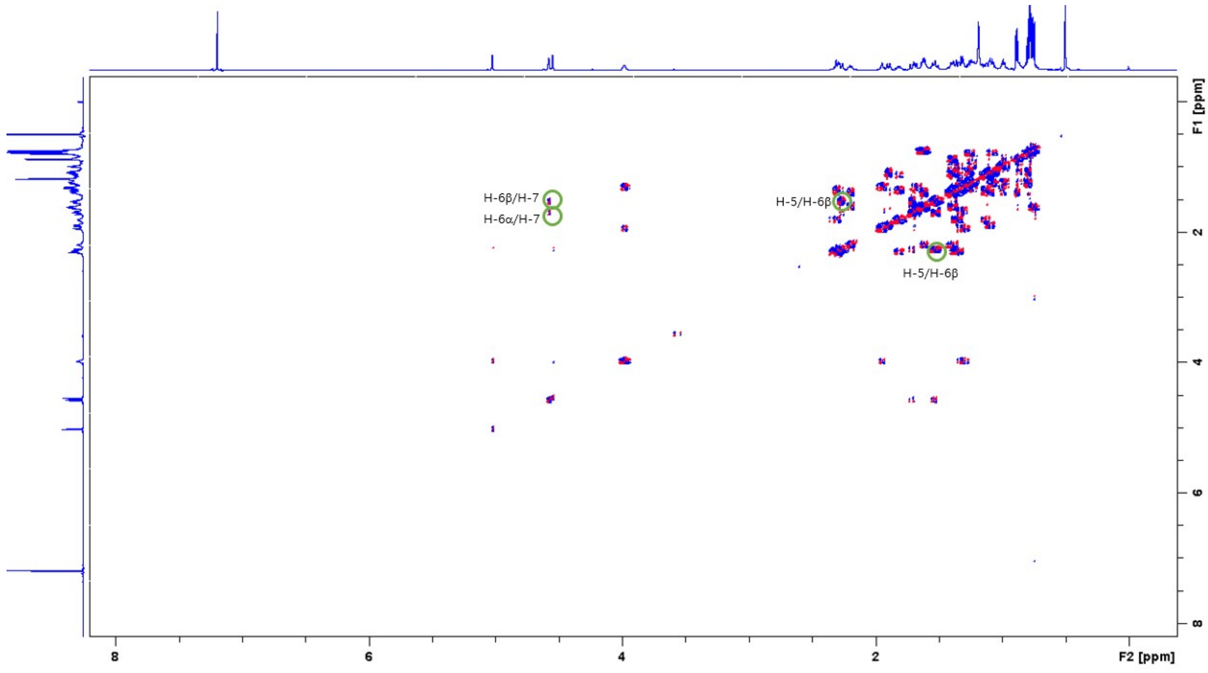

Figure SII-38. ^{1}H-^{1}H COSY spectrum of compound **6** ($CDCl_3$, 600 MHz).

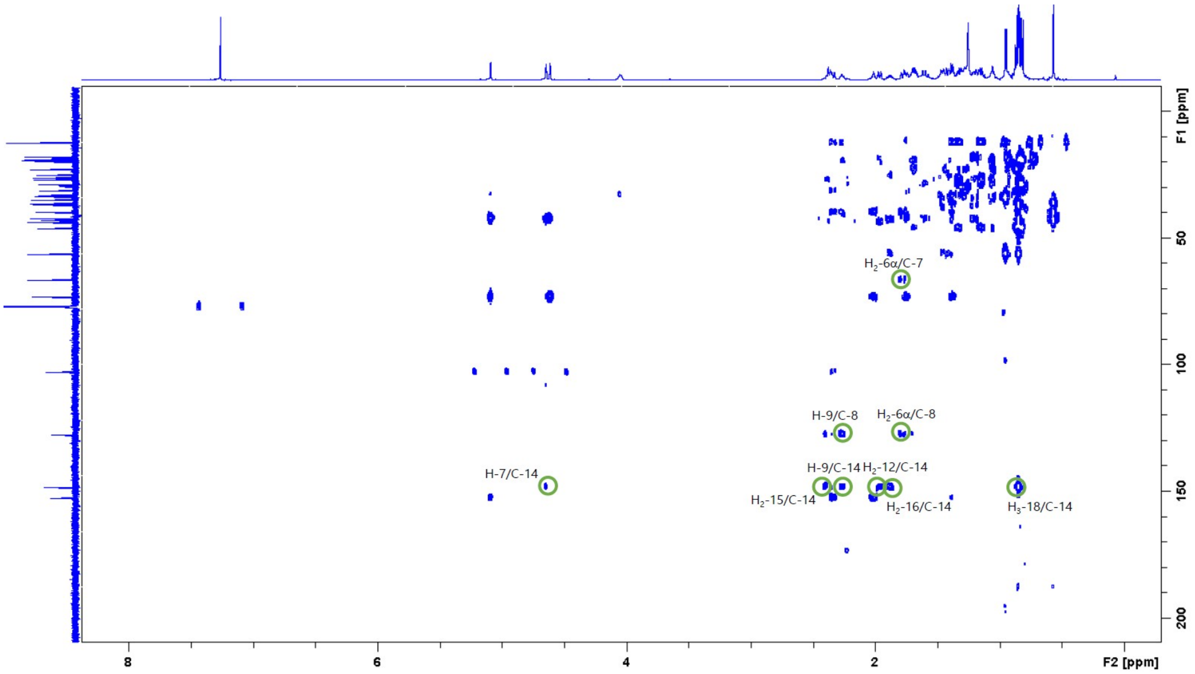

Figure SII-39. HMBC spectrum of compound **6** ($CDCl_3$, 600 MHz).

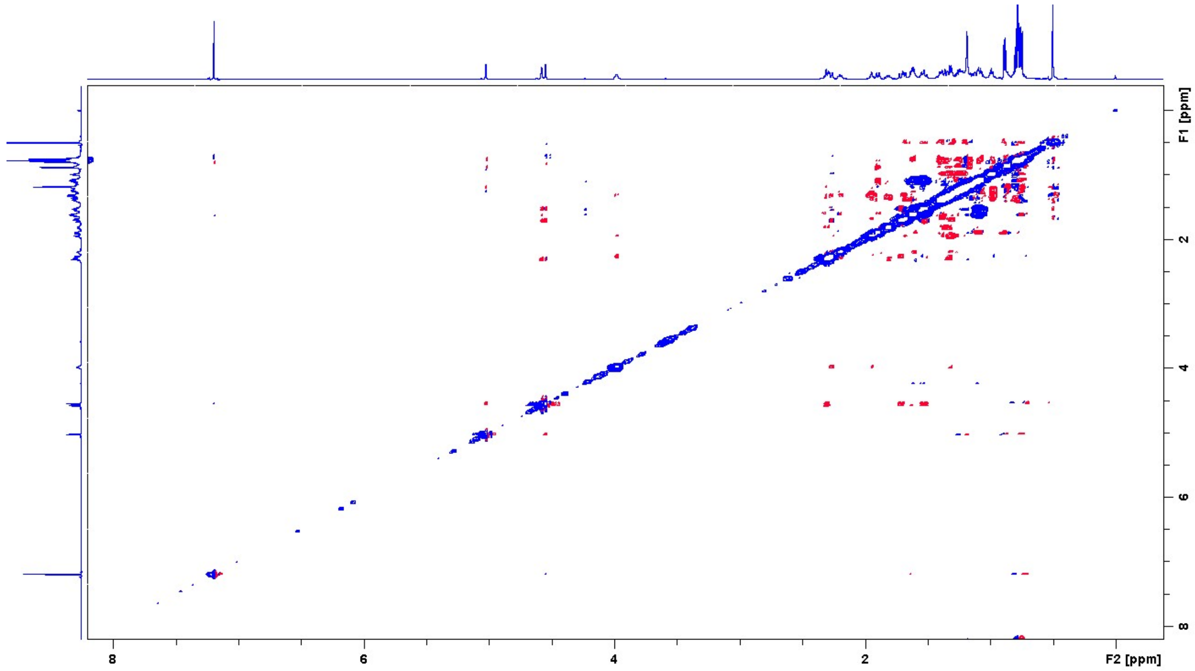

Figure SII-40. NOESY spectrum of compound **6** ($CDCl_3$, 600 MHz).

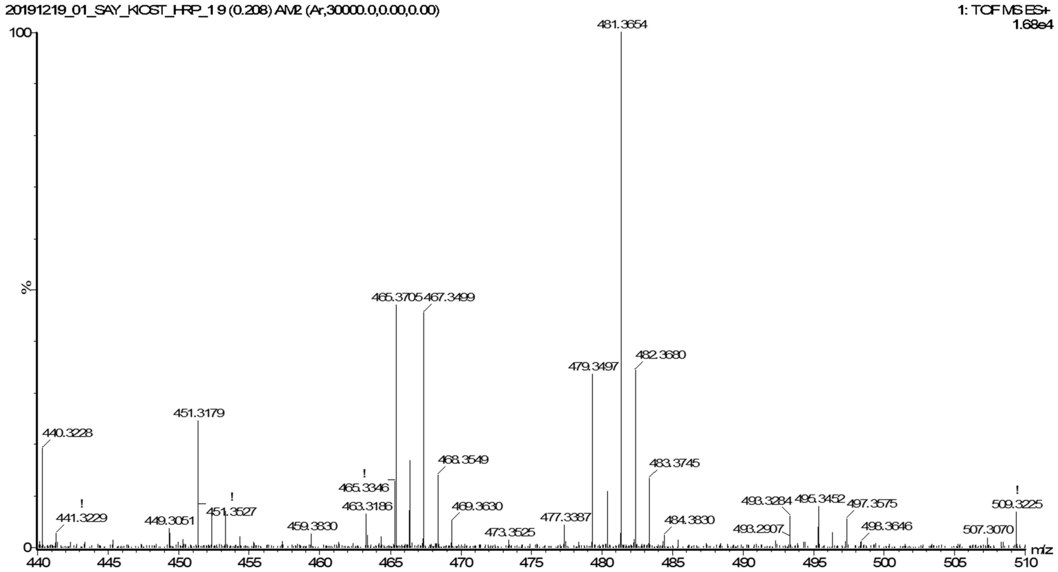

Figure SII-41. HRMS spectrum of compound **6**.

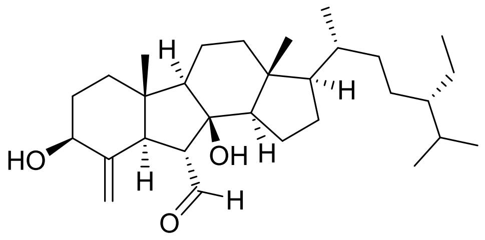

7

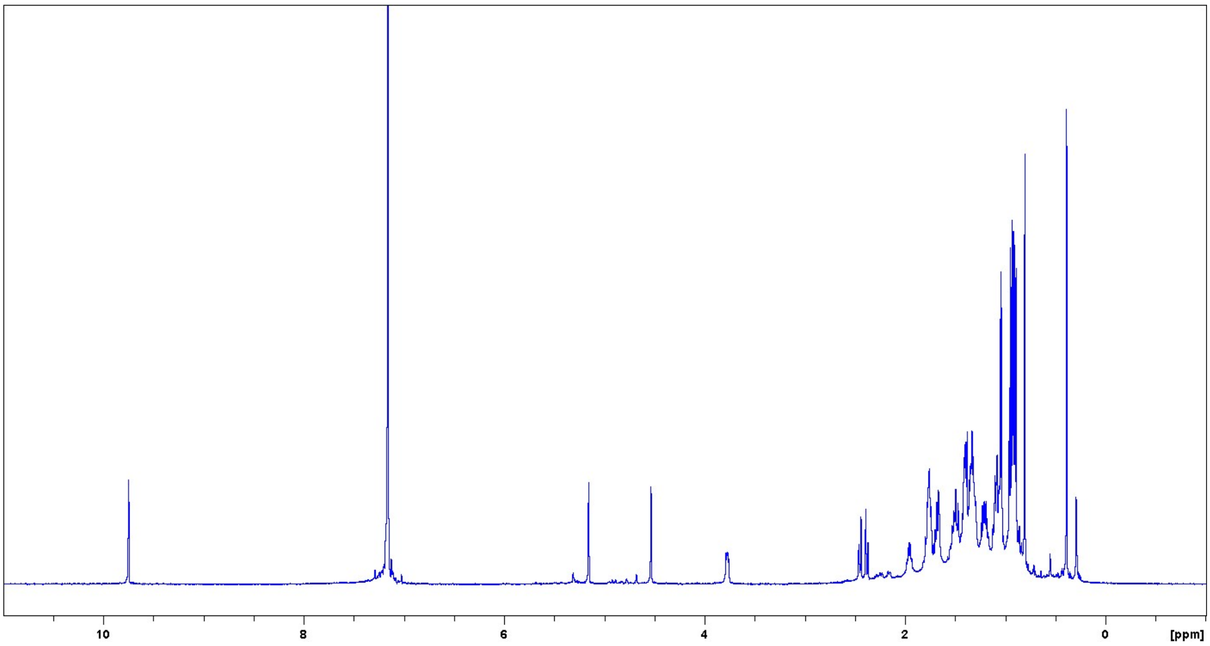

Figure SII-42. ^{1}H NMR spectrum of compound **7** (C_6D_6, 600 MHz).

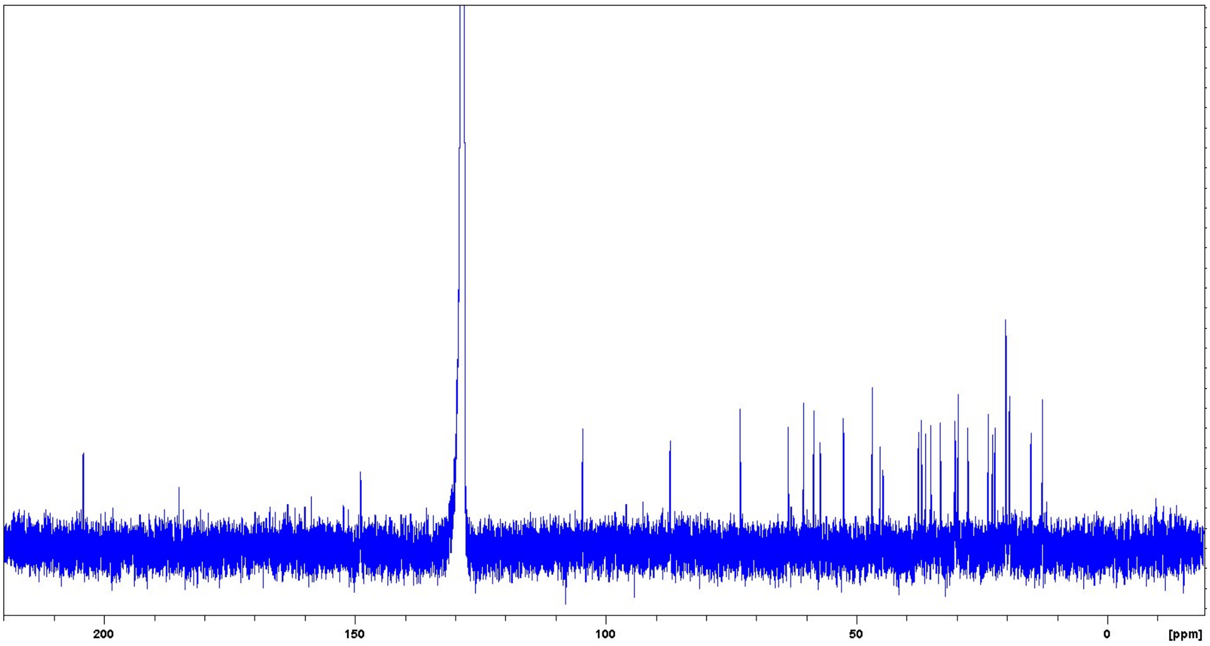

Figure SII-43. ^{13}C NMR spectrum of compound **7** (C_6D_6, 150 MHz).

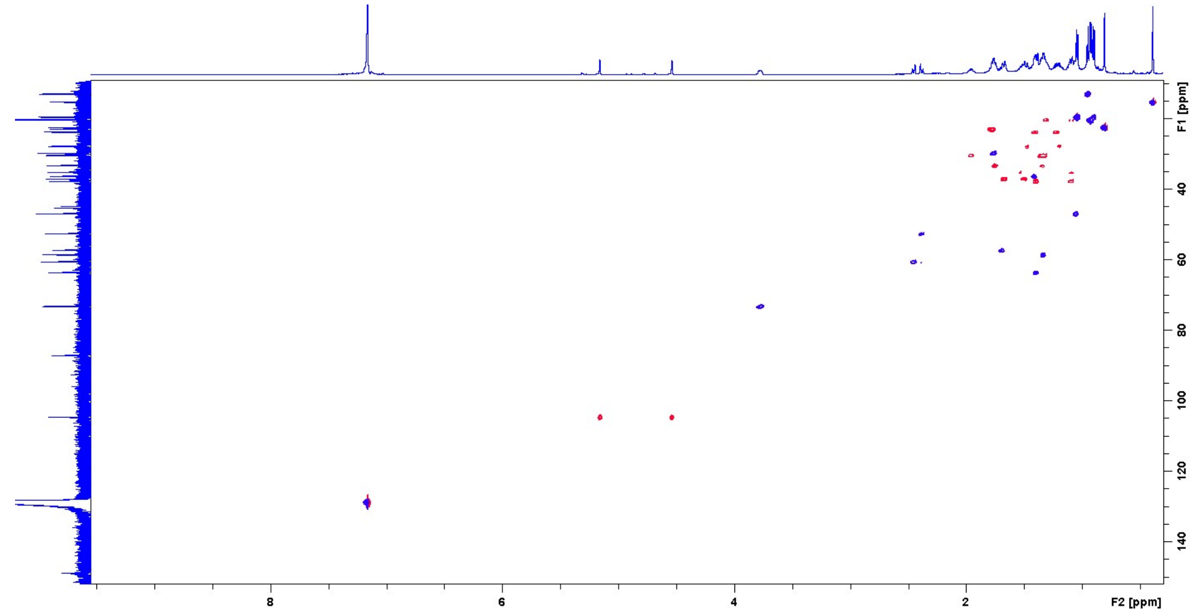

Figure SII-44. HSQC spectrum of compound **7** (C_6D_6, 600 MHz).

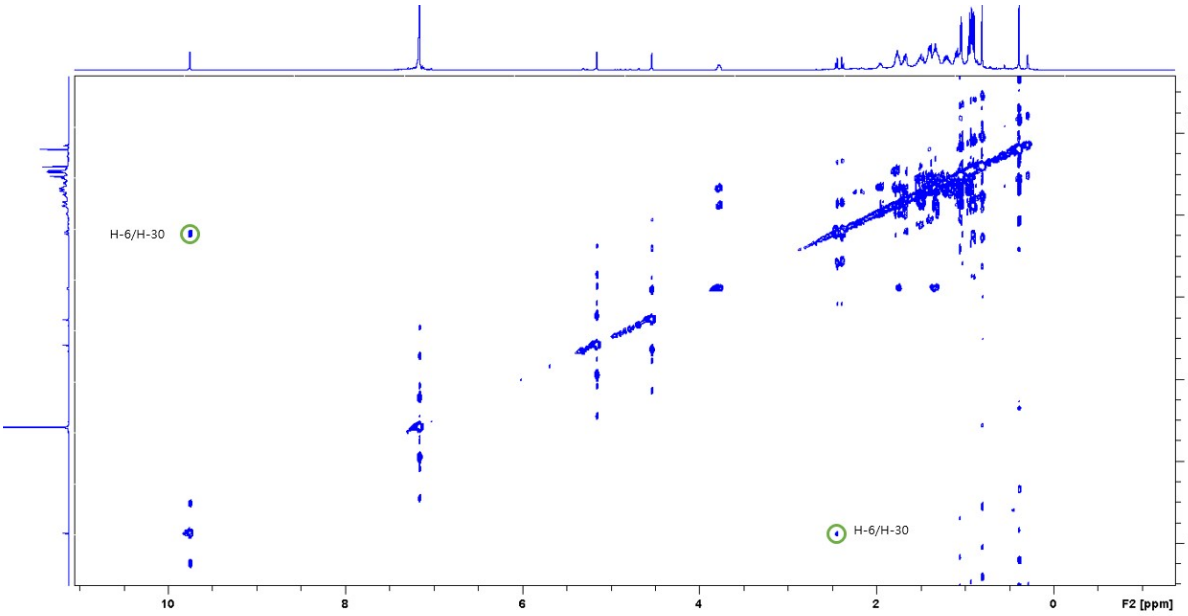

Figure SII-45. ^{1}H-^{1}H COSY spectrum of compound **7** (C_6D_6, 600 MHz).

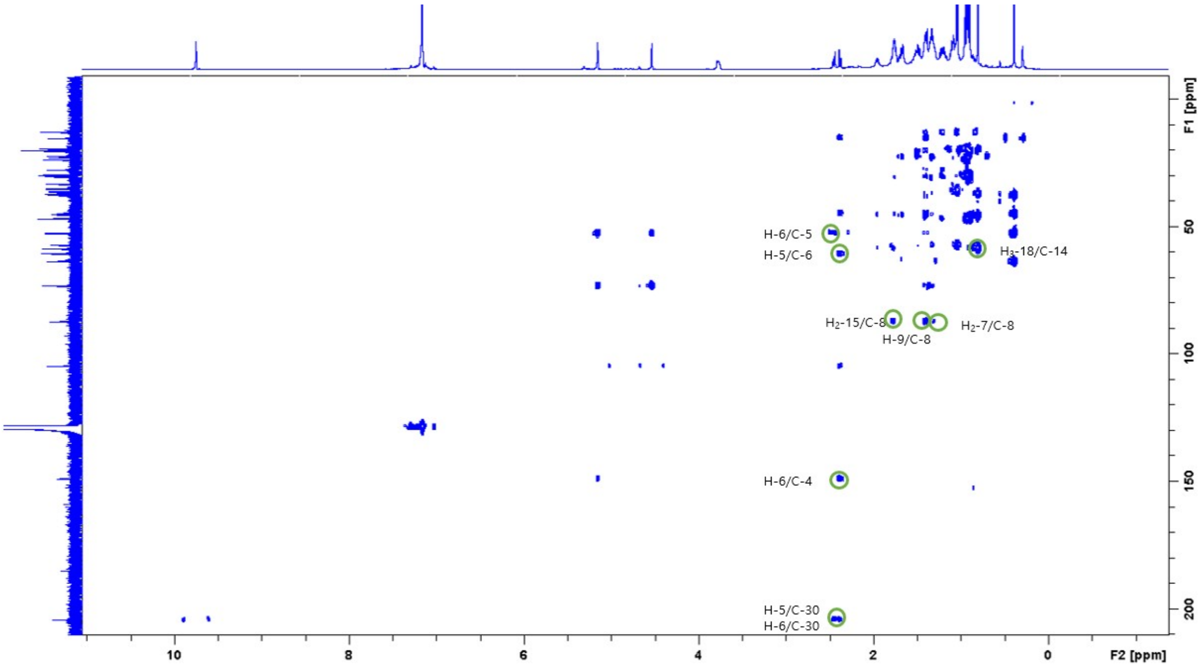

Figure SII-46. HMBC spectrum of compound **7** (C_6D_6, 600 MHz).

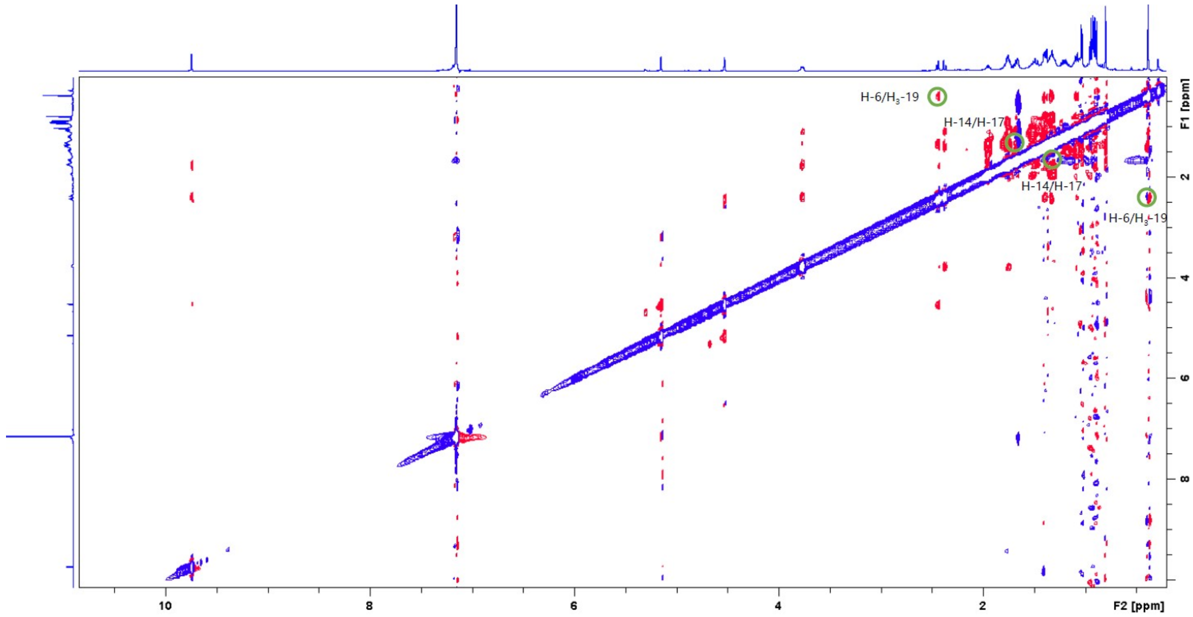

Figure SII-47. NOESY spectrum of compound **7** (C_6D_6, 600 MHz).

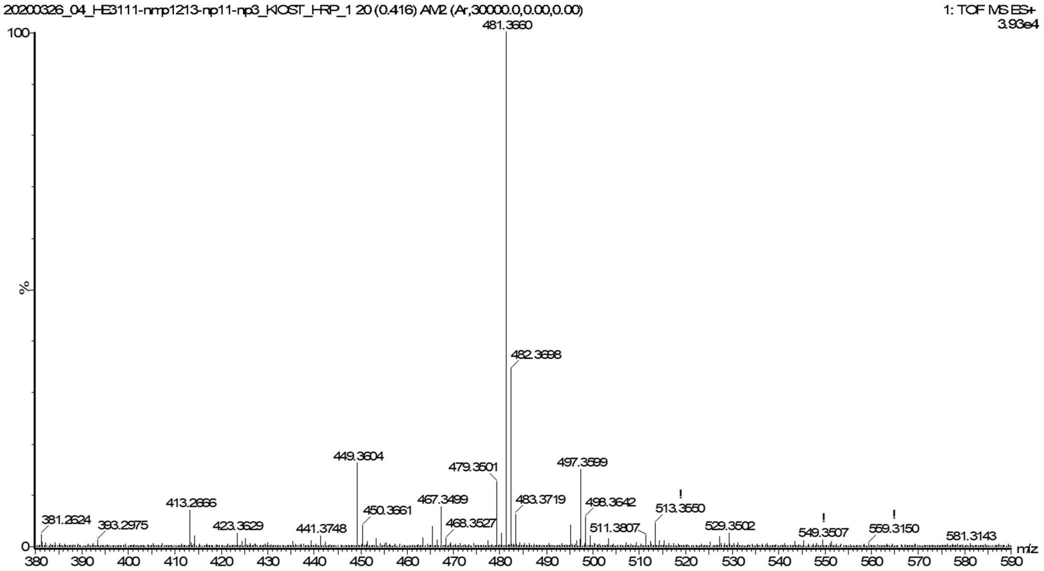

Figure SII-48. HRMS spectrum of compound **7**.

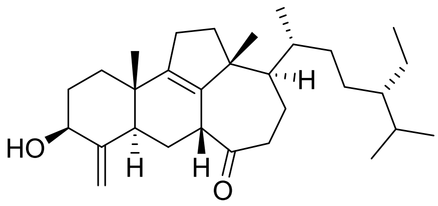

8

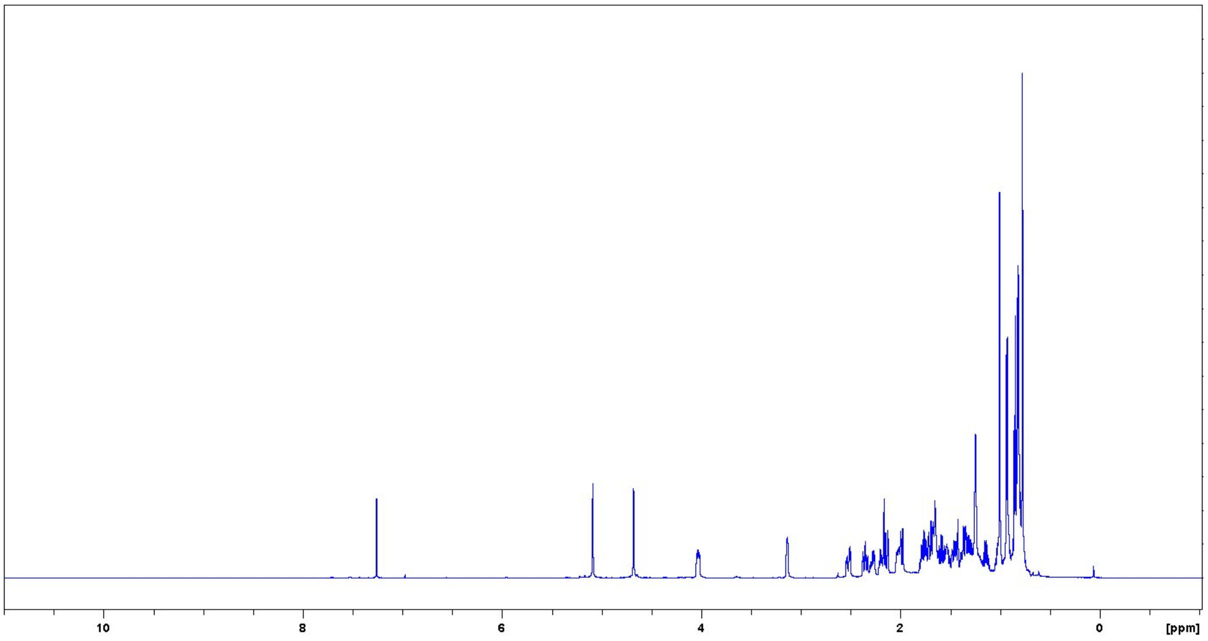

Figure SII-49. ^{1}H NMR spectrum of compound **8** ($CDCl_3$, 600 MHz).

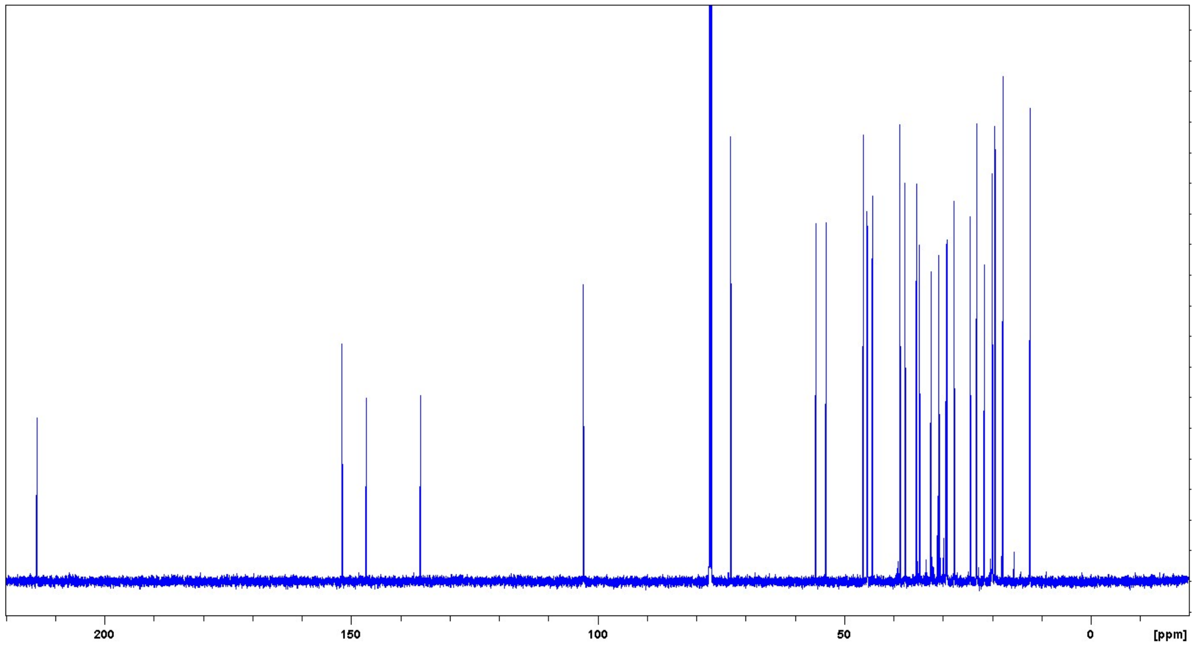

Figure SII-50. ^{13}C NMR spectrum of compound **8** ($CDCl_3$, 150 MHz).

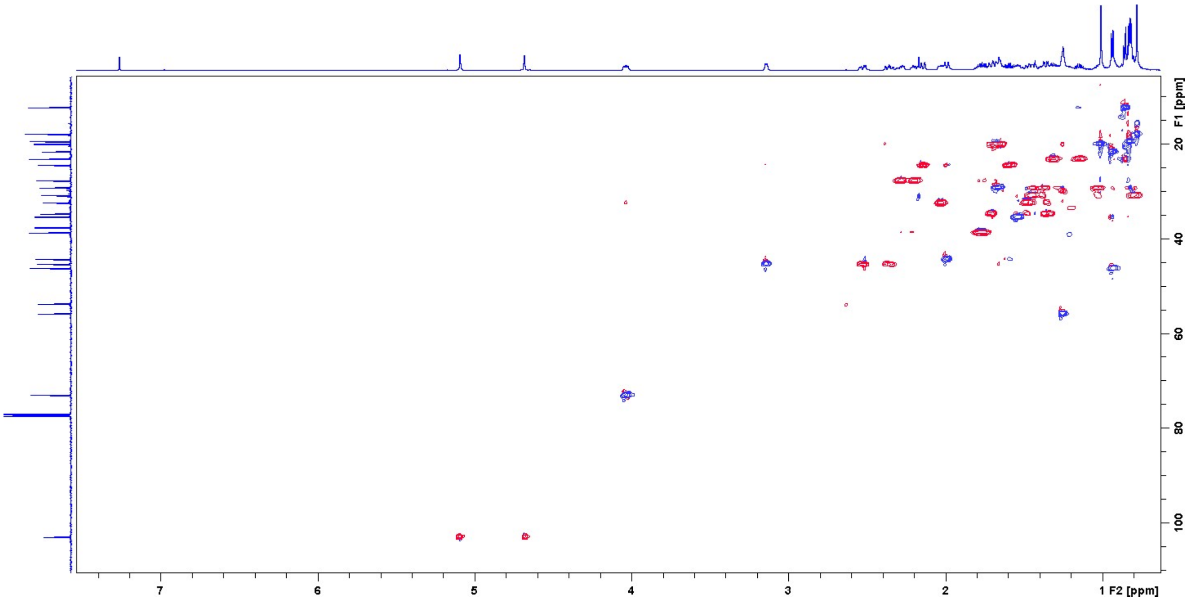

Figure SII-51. HSQC spectrum of compound **8** ($CDCl_3$, 600 MHz).

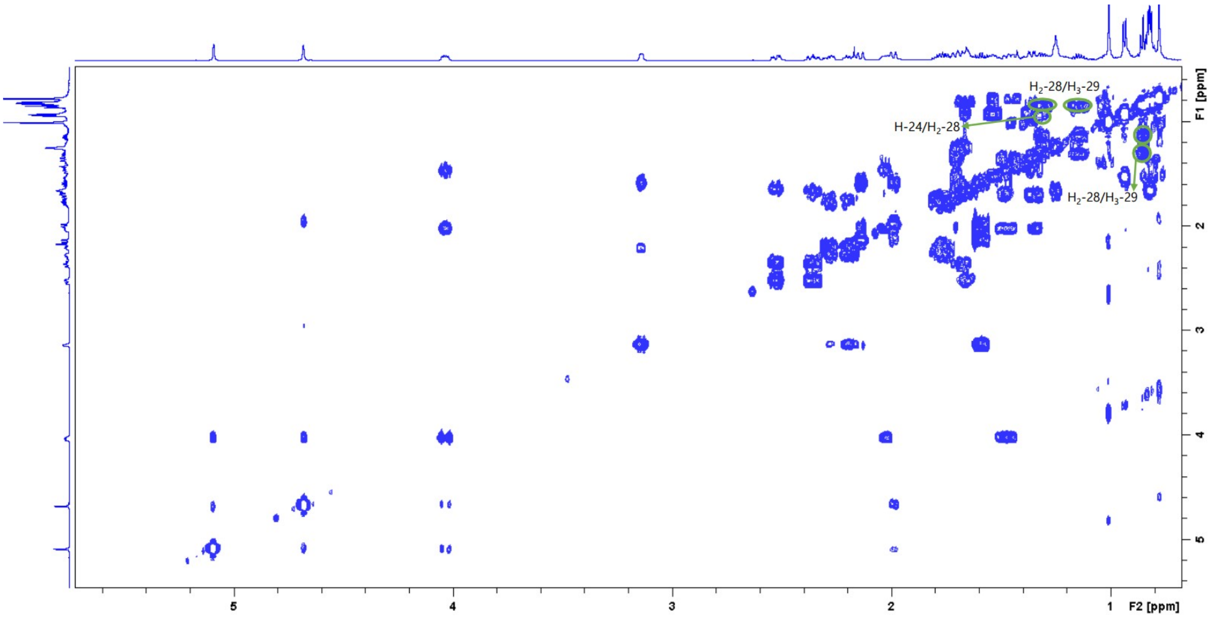

Figure SII-52. ^1H-^1H COSY spectrum of compound **8** ($CDCl_3$, 600 MHz).

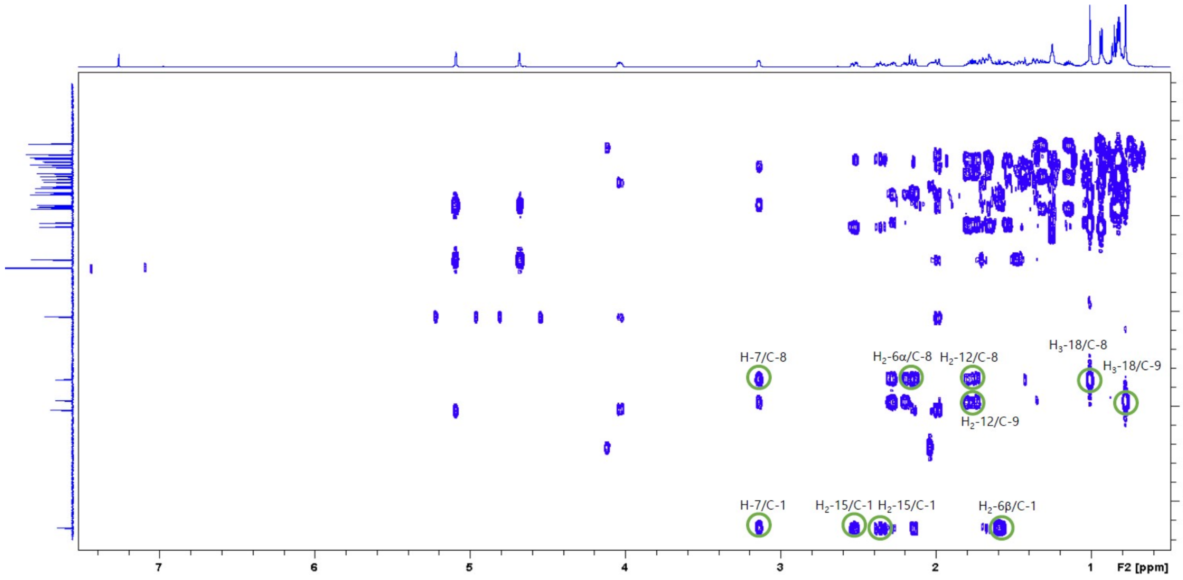

Figure SII-53. HMBC spectrum of compound **8** ($CDCl_3$, 600 MHz).

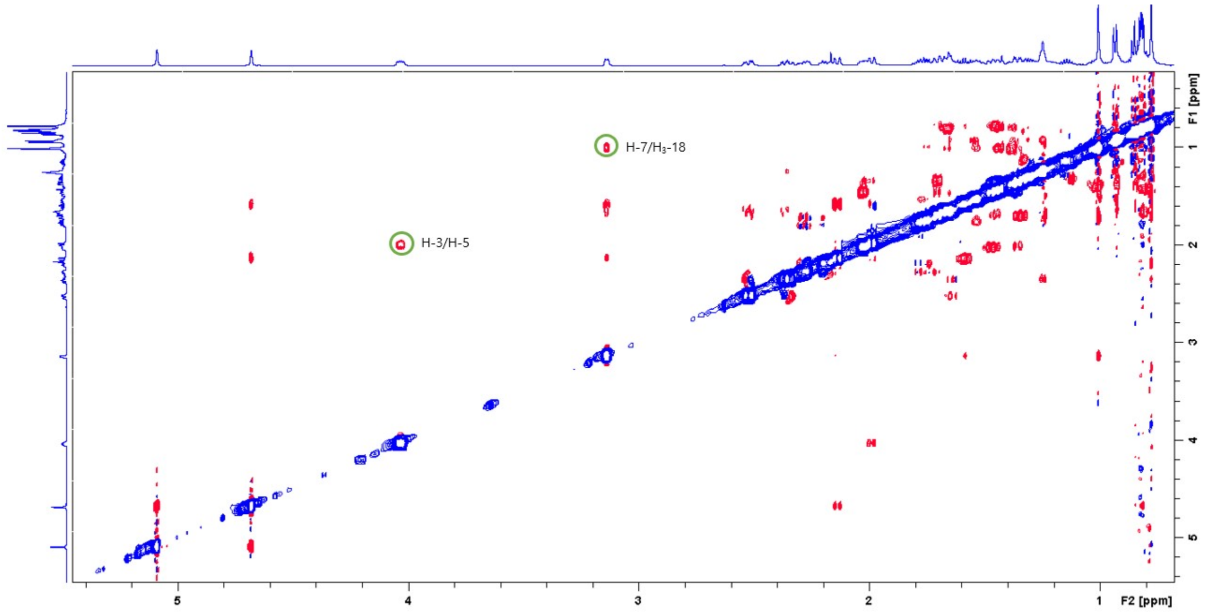

Figure SII-54. NOESY spectrum of compound **8** ($CDCl_3$, 600 MHz).

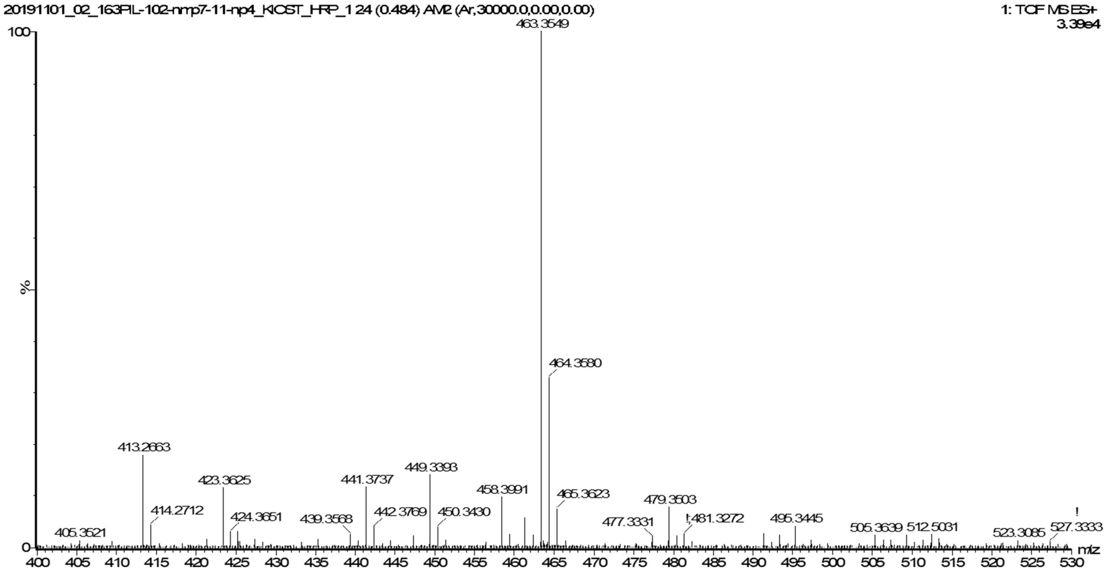

Figure SII-55. HRMS spectrum of compound **8**.

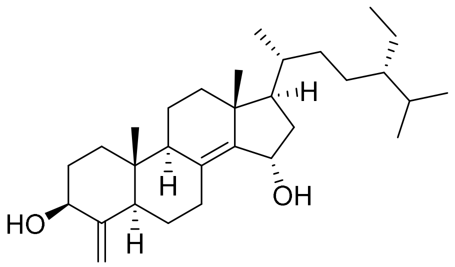

9

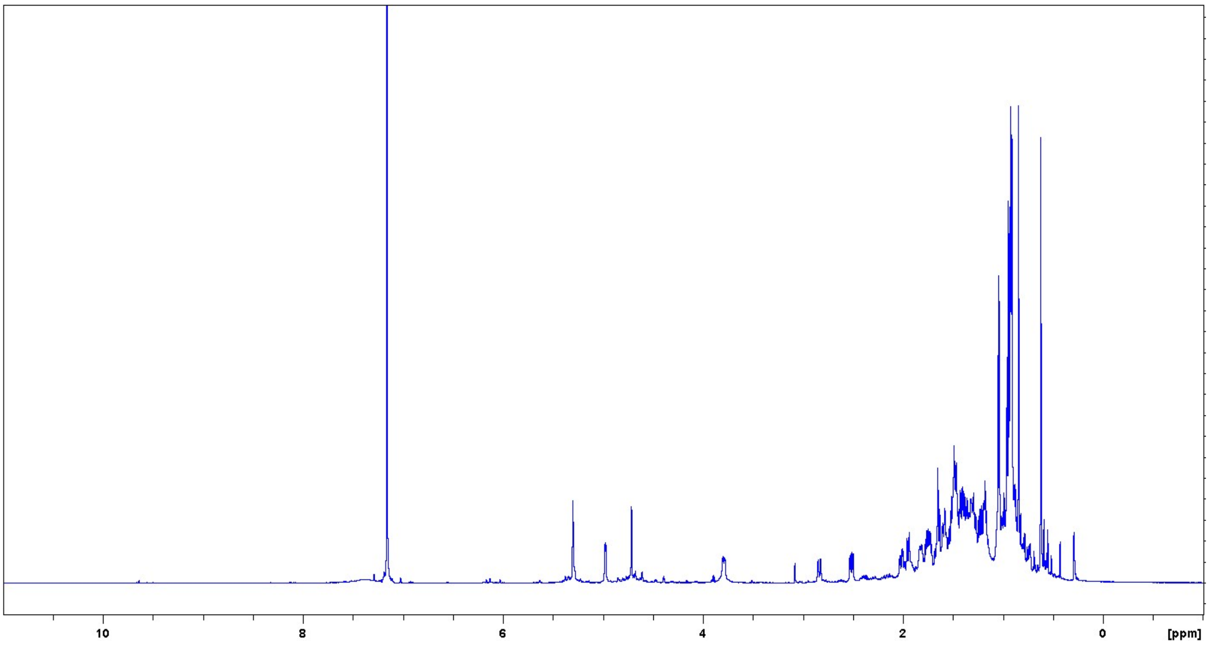

Figure SII-56. ^{1}H NMR spectrum of compound **9** (C_6D_6, 600 MHz).

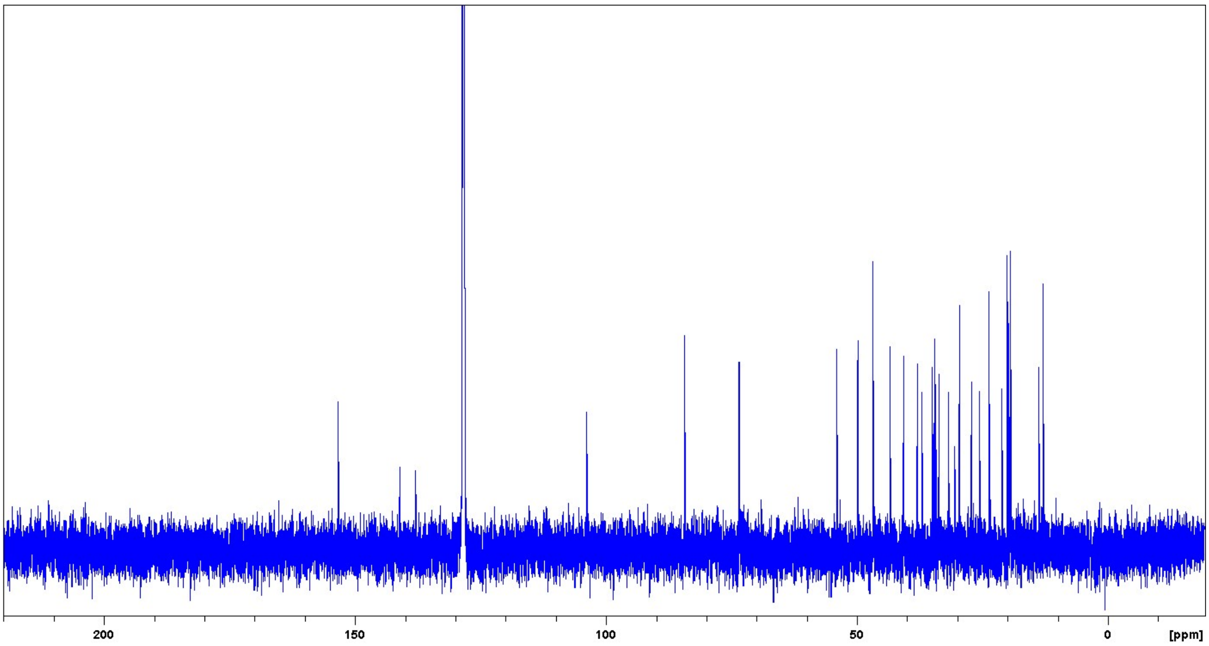

Figure SII-57. ^{13}C NMR spectrum of compound **9** (C_6D_6, 150 MHz).

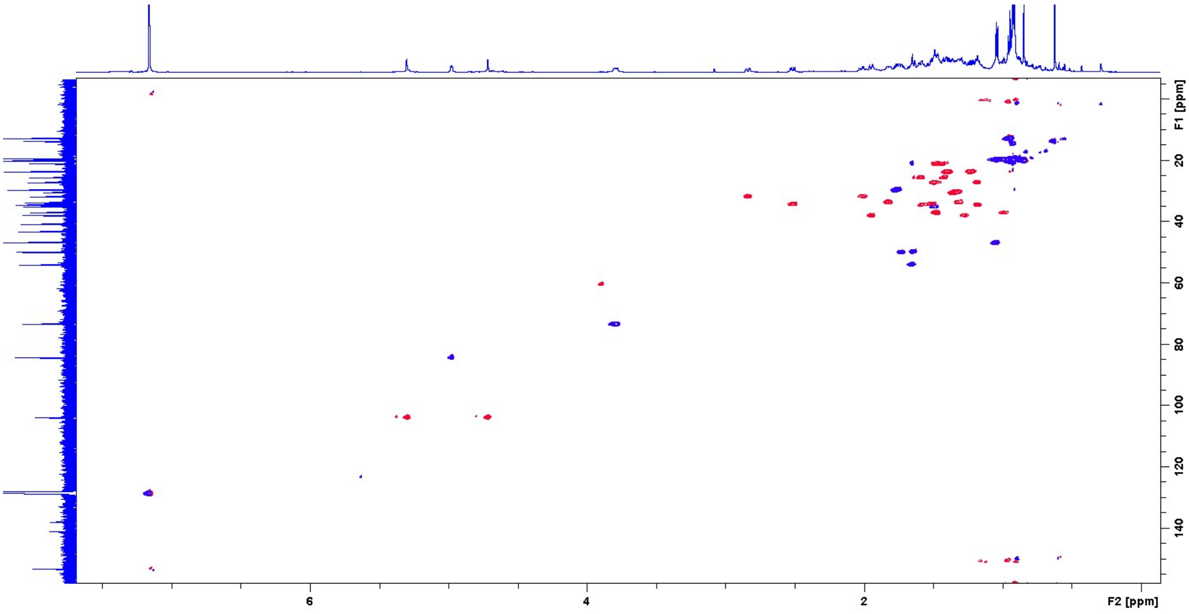

Figure SII-58. HSQC spectrum of compound **9** (C_6D_6, 600 MHz).

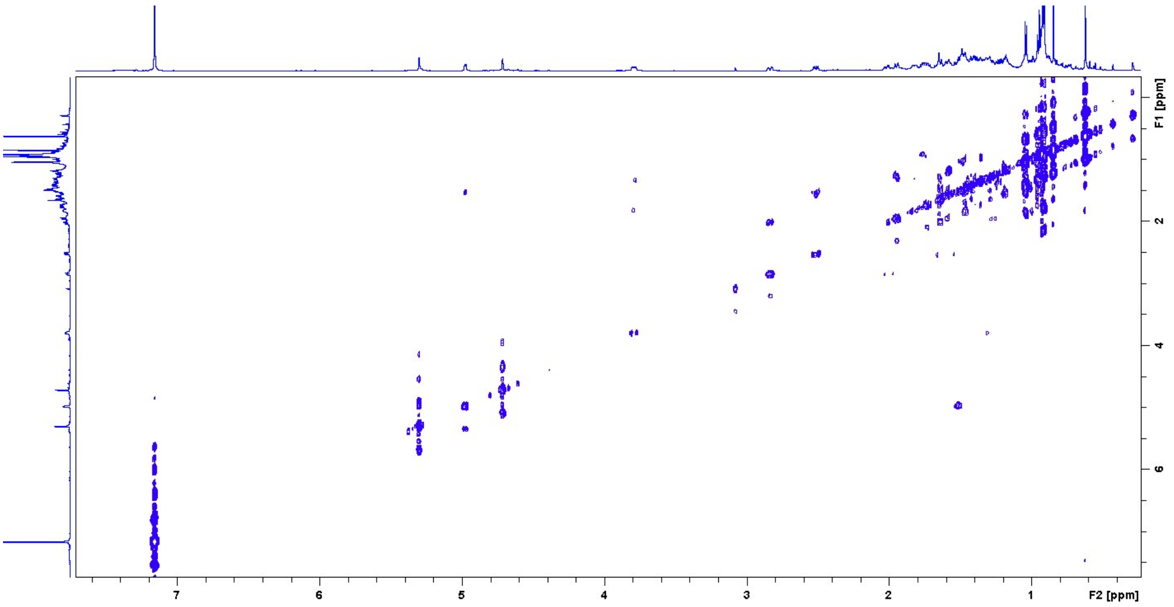

Figure SII-59. ^{1}H-^{1}H COSY spectrum of compound **9** (C_6D_6, 600 MHz).

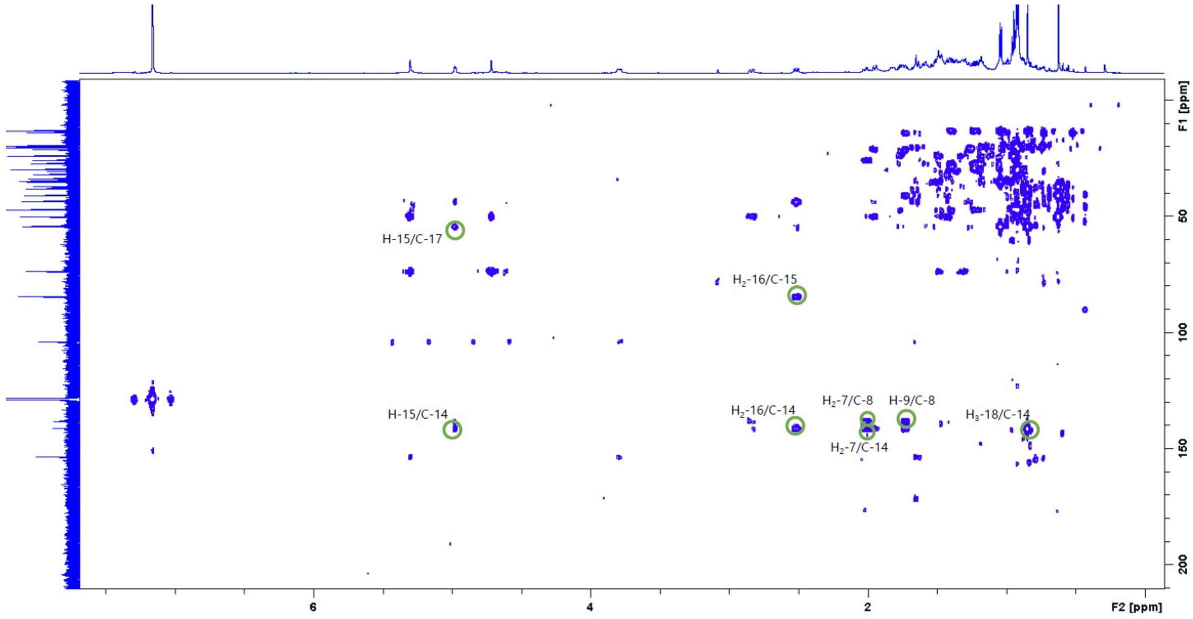

Figure SII-60. HMBC spectrum of compound **9** (C_6D_6, 600 MHz).

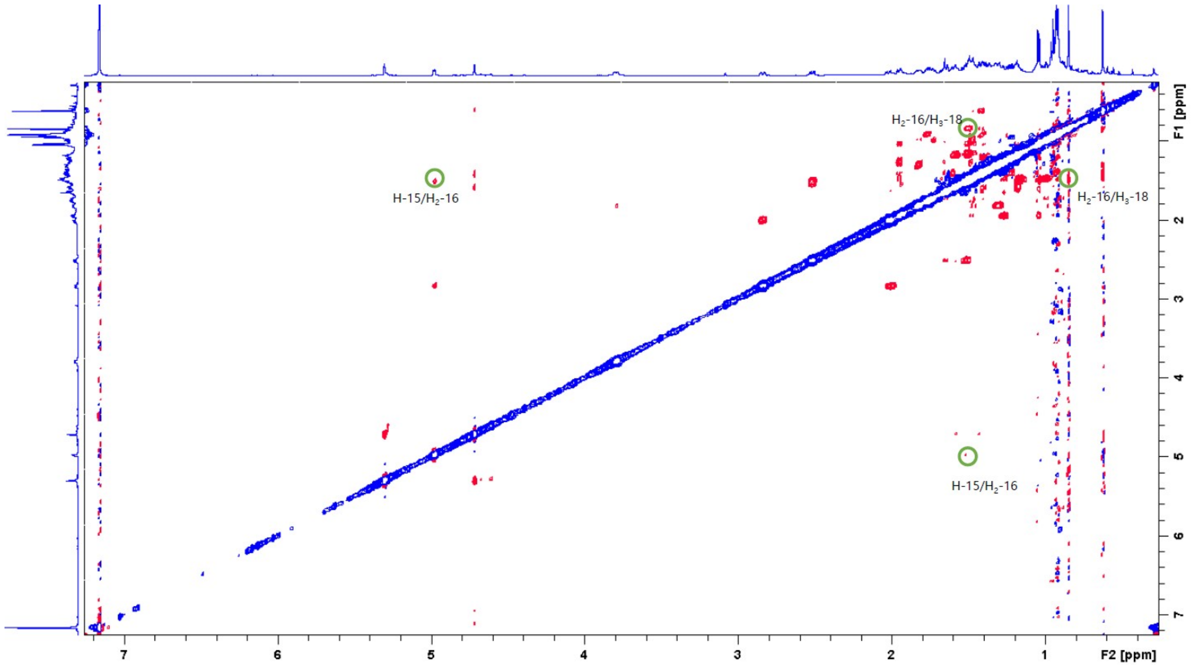

Figure SII-61. NOESY spectrum of compound **9** (C_6D_6, 600 MHz).

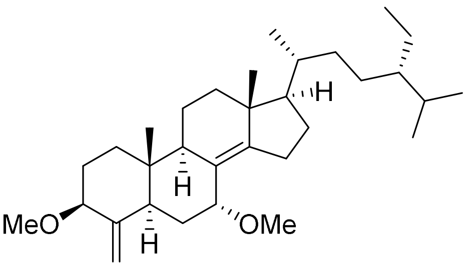

10

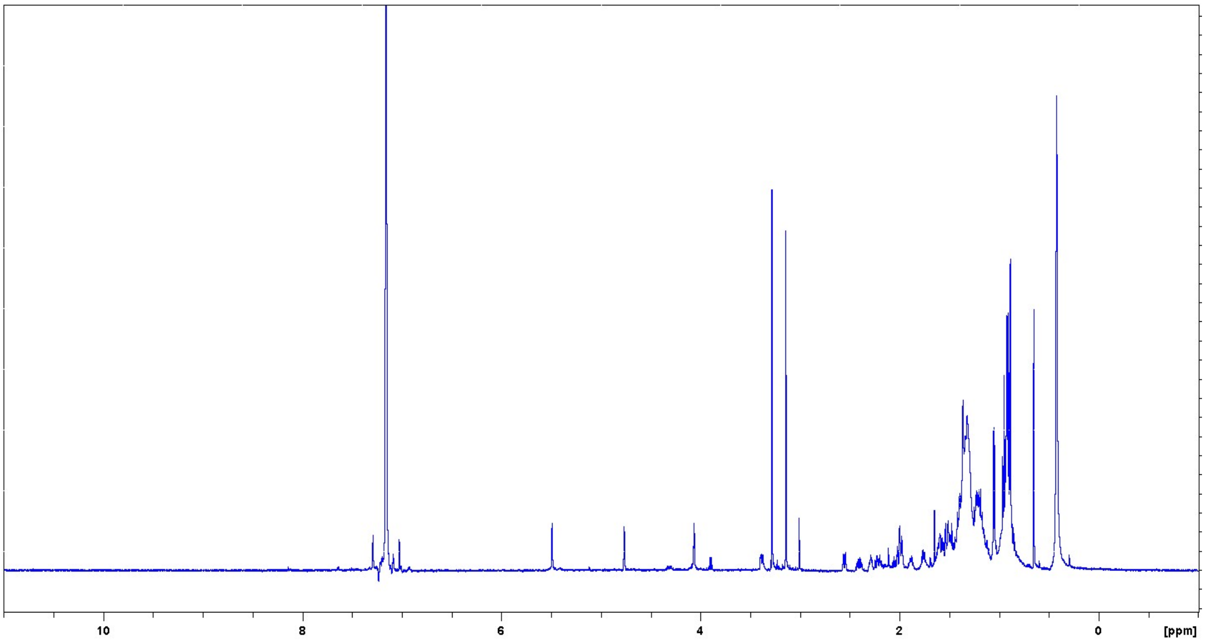

Figure SII-62. ^{1}H NMR spectrum of 3-OMe-swinhosterol C **10** (C_6D_6, 600 MHz).

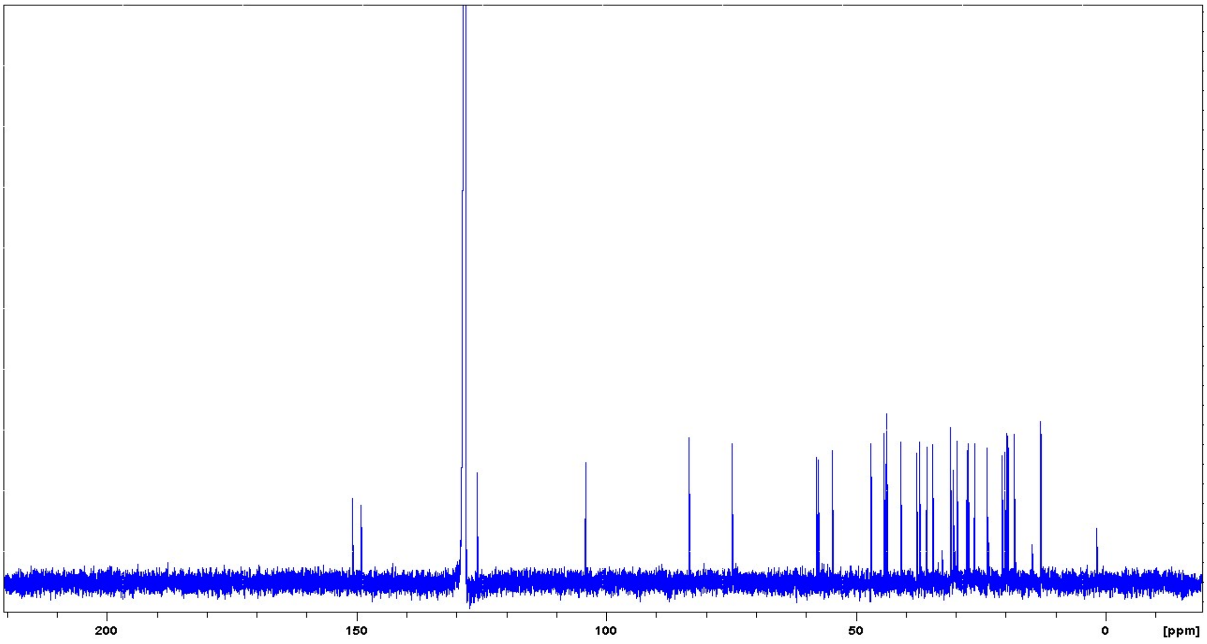

Figure SII-63. ^{13}C NMR spectrum of 3-OMe-swinhosterol C **10** (C_6D_6, 150 MHz).

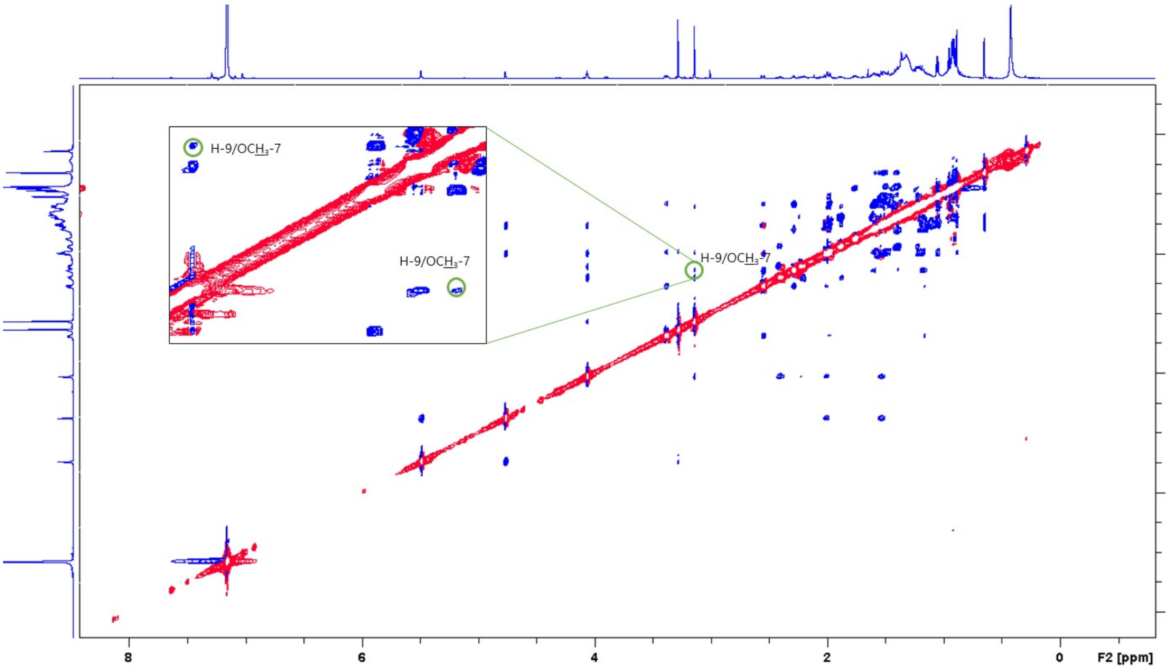

Figure SII-64. NOESY spectrum of 3-OMe-swinhosterol C **10** (C_6D_6, 600 MHz).

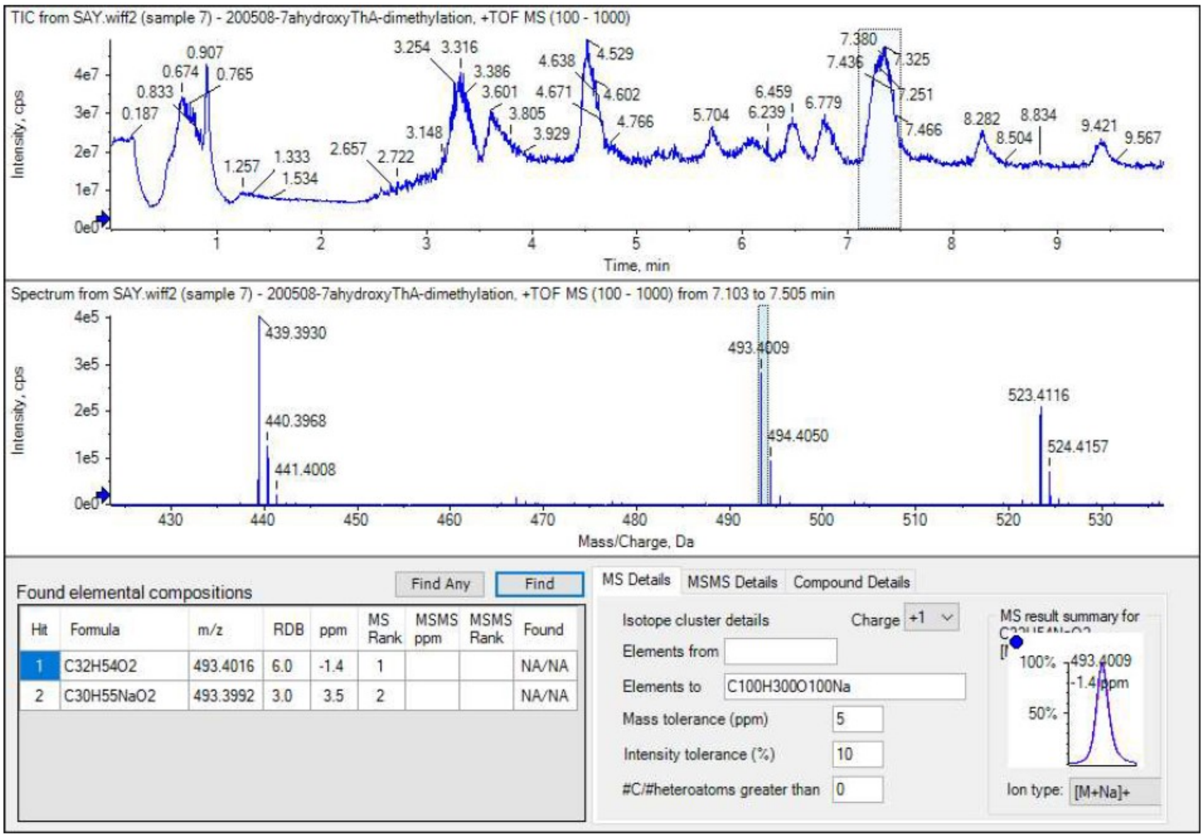

Figure SII-65. HRMS spectrum of compound **10.**
