# Supplementary material for: Oxygenated Theonellastrols: Interpretation of Unusual Chemical Behaviors Using Quantum Mechanical Calculations and Stereochemical Reassignment of 7α-Hydroxytheonellasterol
Source: Mar Drugs. 2020 Nov 30;18(12):607. doi: 10.3390/md18120607 (PMC7760259; doi:10.3390/md18120607)
Supplement: Supplementary file 1 [file marinedrugs-18-00607-s001.zip › SI_revision/SI_revision.pdf]

Supporting Information I

Experimental section

Interpretation of Unusual Chemical Behaviors of Oxygenated theonellastrols using Quantum Mechanical Calculations and Stereochemical reassignment of 7α-hydroxytheonellasterol

A-Young Shin,[1,2] *Hyi-Seung Lee,* [1,2] *Yeon-Ju Lee,* [1,2] *Jong Seok Lee,* [1,2] *Arang Son,*[3] *Changhoon Cho,*[3]

*and Jihoon Lee**[1,2]

[1]Korea Institute of Ocean Science and Technology (KIOST), Busan 49111, Republic of Korea

[2]Department of Marine Biotechnology, University of Science and Technology (UST), Daejeon 34113, Republic of Korea

[3]Department of Radiation Oncology, Samsung Medical Center, Seoul 06351, Republic of Korea

Table of contents

I. Experimental procedure 4

1. General information 4

2. Synthesis of compound **10** 4

Figure S1. Structures of compounds **1-9**. 5

Figure S2. Structures of known compounds (**11-16**) isolated from *T. swinhoei* 5

Table S1. ^{13}C NMR (150 MHz) data for **1-9.** 6

Table S2. ^{1}H NMR (600 MHz) data for **1-9.** 7

Figure S3. Key COSY, HMBC, and NOESY correlations of compounds **1-10**. 9

II. Computational methods 10

1. Geometry optimization 10

2. Transition state for transformation of compound **4** to **9** 11

Figure S4. (a) Free energy diagram for 1,3-hydroxyl migration in 8α-hydroxy-theonellasterol **4** to generate 15α-hydroxy-theonellasterol **9**. (b) Bond lengths for C-8–C-14 and C-14–C15 and atomic distance between C-15 and O-8 in the TS structure. 11

Table S3. Parameters of optimized structures for compounds **4**, **9**, and **TS** in the gas phase. 11

3. ^{13}C chemical shift calculations 12

Table S4. The optimized structures of isomers **I–IV** used for ^{13}C chemical shift calculations. 12

Table S5. Parameters of optimized isomers **I–IV** in the gas phase. 12

Table S6. Comparison between experimental and calculated ^{13}C chemical shifts in C_6D_6 for isomers **I–IV**. 13

Figure S5. Linear regression analyses of the experimental and calculated ^{13}C NMR data for isomers **I-IV**. 14

Figure S6. Comparison of $|\delta_{exptl}–\delta_{calcd}|$ values (parts per million) in C_6D_6 for isomers **I-IV**. 14

Figure S7. DP4+ probability analyses for isomers **I-IV**. 15

Table S7. Optimized Z-matrixes of isomer **5-I** in the gas phase (Å). 16

Table S8. Optimized Z-matrixes isomer **5-II** in the gas phase (Å). 17

Table S9. Optimized Z-matrixes of **5-III** in the gas phase (Å). 18

Table S10. Optimized Z-matrixes of **5-IV** in the gas phase (Å). 19

III. Determination of the C-24 configuration 20

1. The list of compounds selected for a database 20

2. Calculation of **|**δ_{H-26}-δ_{H-27}**|** values from the selected compounds 21

Table S11. $|\delta_{H-26}$-$\delta_{H-27}|$ values for the (21*R*, 24*S*) and (21*R*, 24*R*)-sets. 21

Table S12. $|\delta_{H-26}$-$\delta_{H-27}|$ values for compounds **1-9.** 21

Figure S8. Comparison of $|\delta_{H-26}$-$\delta_{H-27}|$ values for (21*R*,24*S*)-set, (21*R*,24*R*)-set, and compounds **1-9**. 22

IV. X-ray crystallography data 23

Table S12. Crystal data and structure refinement for 7α-theonellasterol. 23

Table S13. Atomic coordinates (x 10^4) and equivalent isotropic displacement parameters (Å^2 x 10^3) for 7α-theonellasterol. 24

Table S14. Bond lengths [Å] and angles [°] for 7α-theonellasterol. 25

Table S15. Anisotropic displacement parameters (Å^2 x 10^3) for 7α-theonellasterol.[a] 28

Table S16. Hydrogen coordinates (x 10^4) and isotropic displacement parameters (Å^2 x 10^3) for 7α-theonellasterol. 30
Table S17. Hydrogen bonds for 7α-theonellasterol [Å and °].[a] 31
Table S18. Torsion angles [°] for 7α-theonellasterol. 32

I. Experimental procedure

1. General information

Specific optical rotations were obtained on a Rudolph Research Analytical (Autopol III) polarimeter. IR spectra were recorded on a JASCO FT/IR-4100 spectrophotometer. The 1D (^{1}H and ^{13}C) and 2D (COSY, HSQC, HMBC, and NOESY) NMR spectra were taken in C_6D_6 or $CDCl_3$ using a Bruker 600 MHz spectrometer at 298.1 K. ^{1}H NMR spectra were collected after 64 scans, and ^{13}C NMR spectra were collected at a range of 10000~15000 scans depending on the sample concentrations. The mixing time for NOESY experiments was set as 0.3 seconds. Chemical shifts are reported in parts per million relative to C_6D_6 (δ_H 7.16, δ_C 128.4) and $CDCl_3$ (δ_H 7.26, δ_C 77.1). High resolution mass-spectra were obtained on a Waters Q-TOF spectrometer equipped with an ESI source and a JEOL JMS-700 spectrometer with an FAB Source at Korea Basic Science Institute (KBSI). MPLC was performed using the TELEDYNE ISCO CombiFlash Companion with the TELEDYNE ISCO RediSep Normal-phase Silica Flash Column. HPLC was performed on a PrimeLine Binary pump utilizing Silica columns (YMC-Pack Silica, 250 × 10 mm I.D., or 250 × 4.6 mm I.D., 5 μm), the Shodex RI-101, or the UV-M201.

2. Synthesis of compound **10**

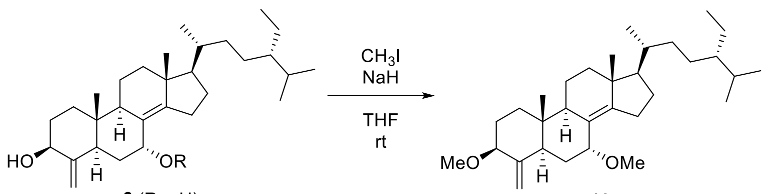

6 (R = H)
swinhosterol C **11** (R = Me)

10

Synthesis of 3-OMe-swinhosterol C **10**: To a solution of **6** (1.0 equiv) in anhydrous THF (0.02 M) was added sodium hydride (5.0 equiv, 60% dispersion in mineral oil) under N_2 atmosphere, and the resulting suspension was stirred for 10 min at 0 °C. Methyl iodide (5.0 equiv) was added and the reaction mixture was stirred for 5 h at room temperature. The reaction was monitored until the stating material disappeared on TLC. After completion of reaction, the reaction mixture was quenched by slow addition of water at 0 °C and diluted with EtOAc. The organic layer was separated, and the aqueous layer was extracted with EtOAc (x 2). The combined organic layers were dried over anhydrous $MgSO_4$, filtered, and concentrated under reduced pressure. The crude material was purified by column chromatography on SiO_2 (hexane/EtOAc 20:1) to afford **10** as a white powder (70% yield). $[\alpha]_D^{25}$ = +23.33 (*c*, 0.1, MeOH);

IR ν_{max} (ATR) 2957, 2953, 2872, 2353, 1593, 1102 cm^{-1};

^{1}H NMR (600 MHz, C_6D_6) : δ 5.49 (s, 1H), 4.77 (s, 1H), 4.06 (t, *J* = 3.0 Hz, 1H), 3.38 (dd, *J* = 11.3, 5.0 Hz, 1H), 3.28 (s, 3H), 3.14 (s, 3H), 2.56 (br d, *J* = 12.0 Hz, 1H), 2.45 - 2.38 (m, 1H), 2.29 (td, *J* =7.9, 3.4 Hz, 1H), 2.25 - 2.17 (m, 1H), 2.04 - 1.96 (m, 3H), 1.92 - 1.86 (m, 1H), 1.78 - 1.74 (m, 1H), 1.63 -1.35 (m, 7H), 1.26 - 1.09 (m, 8H), 1.05 (d, *J* = 6.6 Hz, 3H), 1.05 (m, 1H), 0.95 (t, *J* = 7.4 Hz, 3H), 0.92 (d, *J* = 6.9 Hz, 3H), 0.91 (d, *J* = 6.9 Hz, 3H), 0.89 (s, 3H), 0.65 (s, 3H);

^{13}C NMR (150 MHz, C_6D_6) : δ 150.7, 149.0, 125.8, 104.1, 83.4, 74.8, 57.9, 57.5, 54.7, 46.9, 44.3, 44.1, 43.8, 40.9, 37.9, 37.3, 35.8, 34.6, 31.2, 31.0, 29.8, 27.8, 27.5, 26.3, 23.8, 20.7, 20.2, 19.9, 19.6, 18.3, 13.1, 12.9;

HRESIMS *m/z* calcd for $C_{32}H_{54}O_2Na$ [M+Na]$^+$ 493.4016, found: 493.4009.

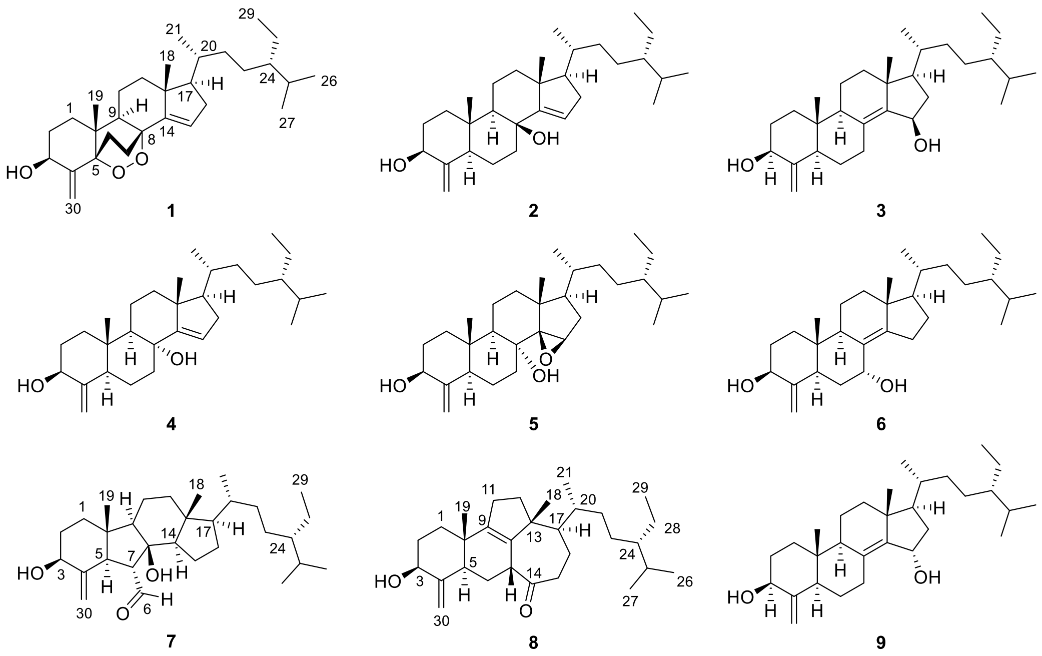

Figure S1. Structures of compounds **1-9**.

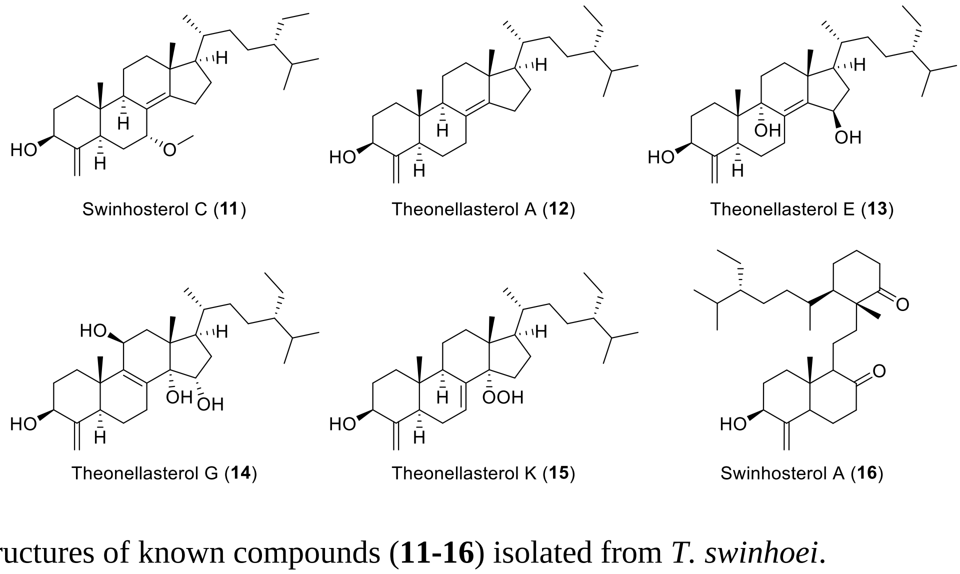

Figure S2. Structures of known compounds (**11-16**) isolated from *T. swinhoei*.

Table S1. ^{13}C NMR (150 MHz) data for **1-9**.

Position	δ_C								
	**1**[a]	**2**[a]	**3**[a]	**4**[a]	**5**[a]	**6**[b]	**7**[a]	**8**[b]	**9**[a]
1	37.8	38.4	37.2	40.8	41.5	36.7	37.7	34.7	37.1
2	32.1	33.4	33.8	33.2	33.3	33.2	33.3	32.4	33.7
3	69.8	73.5	73.6	73.6	73.8	73.4	73.2	73.0	73.5
4	150.6	153.3	153.4	153.8	154.2	152.8	148.9	151.8	153.4
5	91.9	51.0	49.89	41.1	41.2	42.5	52.6	44.3	49.8
6	30.6	21.0	25.5	19.7	20.3	31.5	204.2	24.5	25.6
7	31.5	31.2	31.2	31.9	28.3	66.7	60.6	45.3	31.8
8	86.8	83.5	134.1	86.6	71.3	127.7	20.3	135.8	138.0
9	58.3	56.9	49.93	46.5	55.3	43.9	87.2	146.9	50.0
10	44.2	39.8	40.7	37.7	38.2	39.9	63.6	37.6	40.8
11	21.6	19.0	21.2	22.7	23.3	20.0	44.8	27.7	21.2
12	44.1	43.6	38.5	39.0	34.7	37.0	20.3	38.6	38.0
13	49.4	47.7	43.8	48.0	42.5	43.1	37.1	53.7	43.5
14	152.6	151.2	148.4	155.8	75.9	148.5	45.4	213.7	141.1
15	116.8	126.2	70.3	123.2	58.8	25.3	58.6	45.4	84.4
16	36.5	35.8	39.6	35.2	32.3	27.1	22.9	20.1	34.4
17	60.6	60.2	54.2	60.0	49.8	56.4	30.4	55.8	54.1
18	19.0	18.1	19.93	17.4	15.0	18.10	57.3	19.9	20.1
19	20.1	15.1	13.8	16.9	17.2	12.5	22.5	17.8	13.9
20	34.8	34.7	35.0	34.9	34.4	35.0	15.3	35.3	35.1
21	19.6	19.6	19.6	19.5	19.2	19.2	36.3	21.6	19.9
22	34.6	34.5	34.7	34.5	34.1	33.8	19.6	30.8	34.6
23	26.9	27.0	27.2	27.3	27.1	26.3	35.2	29.3	27.3
24	46.9	46.9	47.0	46.9	46.8	46.2	46.9	46.2	46.9
25	29.7	29.7	29.7	29.8	29.6	29.1	29.8	29.2	29.6
26	19.6	19.5	19.92	19.6	19.4	19.3	19.5	19.36	19.5
27	20.0	20.1	20.2	20.2	20.1	19.7	20.3	19.4	20.2
28	23.8	23.8	23.8	23.8	23.7	23.2	23.8	23.2	23.8
29	12.9	12.9	13.0	13.0	12.9	12.5	13.0	12.3	13.0
30	111.2	103.4	103.9	103.7	103.3	102.9	104.7	102.9	103.9

[a] Measured in C_6D_6; [b] Measured in $CDCl_3$; ^{13}C assignments aided by HSQC, HMBC and DEPT135 experiments.

Table S2. ^{1}H NMR (600 MHz) data for **1-9**.

Position	δ_H (J, Hz)								
	**1**[a]	**2**[a]	**3**[a]	**4**[a]	**5**[a]	**6**[b]	**7**[a]	**8**[b]	**9**[a]
1	1.23, m	0.79, m	1.01, m	1.40, m	1.26, m	1.36, m	1.08, m	1.34, m	0.99, m
	1.33, m	1.54, m	1.51, m	1.65, m	1.56, m	1.74, m	1.40, m	1.69, m	1.48, m
2	1.33, m	1.37, m	1.31, m	1.40, m	1.41, m	1.39, m	1.33, m	1.45, m	1.32, m
	2.32, m	1.79, m	1.82, m	1.73, m	1.73, m	2.02, m	1.76, m	2.01, m	1.83, m
3	4.64, m	3.79, dd (11.6, 5.7)	3.80, dd (11.6, 5.3)	3.76, m	3.84, dd (11.9, 5.1)	4.05, dd (10.4, 5.3)	3.77, dd (11.0, 5.9)	4.03, dd (11.4, 5.7)	3.79, dd (11.5, 5.7)
5		1.48, m	1.62, m	3.03, dd (12.0, 5.1)	3.35, m	2.34, brd (13.6)	2.38, d (12.7)	1.95, brd (12.7)	1.64, m
6	1.33, m	1.44, m	1.38, m	1.54, m	1.73, m	1.59, m	9.75, d (3.3)	2.13, td (12.9, 6.4)	1.42, m
	1.77, m	1.89, m	1.54, m	1.67, m	1.91, m	1.77, m		1.58, brd (12.9)	1.58, m
7	1.43, m	1.20, m	1.82, m	1.68, m	1.60, m	4.64, t (2.82)	2.45, dd (12.7, 3.2)	3.13, m	2.01, td (14.0, 5.4)
	1.86, m	2.67, dt (13.4, 3.0)	2.78, ddd (14.2, 4.5, 2.3)	1.76, m	1.82, m				2.84, ddd (14.8, 4.5, 2.2)
9	1.59, m	1.01, m	1.69, m	2.27, dd (12.1, 7.0)	1.41, m	2.26, m	1.40, m		1.73, m
11	1.33, m	1.30, m	1.42, m	1.49, m	1.41, m	1.46, m	1.09, m	2.19, m	1.42-1.46, m
	1.33, m	1.82, m	1.49, m	1.49, m	1.48, m	1.68, m	1.30, m	2.27, m	1.42-1.46, m
12	1.23, m	1.24, m	1.22, m	1.15, m	1.56, m	1.16, m	1.50, m	1.76, m	1.27, m
	1.93, dt (13.0, 2.8)	1.99, m	1.97, m	1.79, m	1.68, m	1.96, dt (12.6, 3.4)	1.68, m	1.76, m	1.95, m
14							1.33 ,m		
15	5.80, dd (3.3, 1.5)	5.52, m	4.64, d (4.8)	5.61, m	3.33, s	2.02, m	1.78, m	2.35, ddd (16.2, 6.1, 2.4)	4.97, d (5.4)
						2.02, m	1.78, m	2.52, ddd (15.8, 11.7, 3.4)	
16	2.01, ddd (15.7, 10.2, 1.4)	1.96, m	1.60, m	1.89, dd (15.6, 10.6)	0.93, m	1.42, m	1.33, m	1.65, m	1.49, m
	2.36, m	2.31, ddd (16.0, 7.2, 3.5)	1.95, m	2.19, ddd (15.6, 7.3, 3.4)	1.96, ddd (13.5, 6.7, 1.1)	1.88, m	1.97, m	1.65, m	2.52, dd (13.9, 6.0)
17	1.68, m	1.60, m	1.60, m	1.51, m	1.41, m	1.20, m	1.69, m	1.24, m	1.65, m
18	0.92, s	1.11, s	0.82, s	0.83, s	0.71, s	0.84, s	0.81, s	1.00, s	0.84, s
19	0.68, s	0.97, s	0.61, s	0.68, s	0.72, s	0.56, s	0.39, s	0.77, s	0.62, s
20	1.59, m	1.60, m	1.48, m	1.57, m	1.24, m	1.46, m	1.41, m	1.53, m	1.49, m
21	0.98, d (6.4)	0.97 ,d (7.2)	1.06, d (6.6)	0.95, m	0.84, d (6.5)	0.95, d (6.6)	1.04, d (6.5)	0.93, d (6.9)	1.04, d (6.6)
22	1.08, m	1.10, m	1.18, m	1.06, m	0.93, m	1.05, m	1.09, m	1.42, m	1.18, m
	1.45, m	1.40, m	1.54, m	1.44, m	1.31, m	1.40, m	1.52, m	1.42, m	1.58, m
23	1.16, m	1.20, m	1.18, m	1.17, m	1.01, m	1.05, m	1.20, m	1.02, m	1.18, m

	1.43, m	1.45, m	1.48, m	1.44, m	1.34, m	1.31, m	1.48, m	1.38, m	1.48, m
24	1.01, m	1.06, m	1.05, m	1.05, m	0.97, m	0.93, m	1.05, m	0.91, m	1.05, m
25	1.73, m	1.77, m	1.75, m	1.76, m	1.70, m	1.69, m	1.76, m	1.65, m	1.76, m
26	0.88, d (6.9)	0.90, d (6.9)	0.91, d (6.8)	0.92, d (7.6)	0.86, d (6.8)	0.82, d (6.9)	0.89, d (6.8)	0.81, d (4.0)	0.91, d (6.8)
27	0.90, d (6.9)	0.92, d (6.9)	0.92, d (6.8)	0.91, d (7.6)	0.88, d (7.0)	0.83, d (6.9)	0.92, d (6.8)	0.82, d (4.0)	0.92, d (6.8)
28	1.21, m	1.23, m	1.23, m	1.22, m	1.15, m	1.16, m	1.23, m	1.13, m	1.23, m
	1.33, m	1.40, m	1.39, m	1.40, m	1.34, m	1.31, m	1.40, m	1.32, m	1.40, m
29	0.93, t (7.4)	0.95, t (7.5)	0.95, t (7.4)	0.95, t (7.3)	0.91, t (7.5)	0.85, t (7.4)	0.95, t (7.4)	0.84, t (7.4)	0.95, t (7.4)
30	5.13, s	4.81, s	4.68, s	4.08, s	4.90, s	4.61, s	4.53, s	4.67, s	4.71, s
	5.59, s	5.26, s	5.28, s	5.37, s	5.40, s	5.08, s	5.16, s	5.08, s	5.30, s
-OH					3.47, d (2.2)				

[a] Measured in C_6D_6; [b] Measured in $CDCl_3$; The assignments were aided by COSY, NOESY, HSQC, and HMBC NMR spectra.

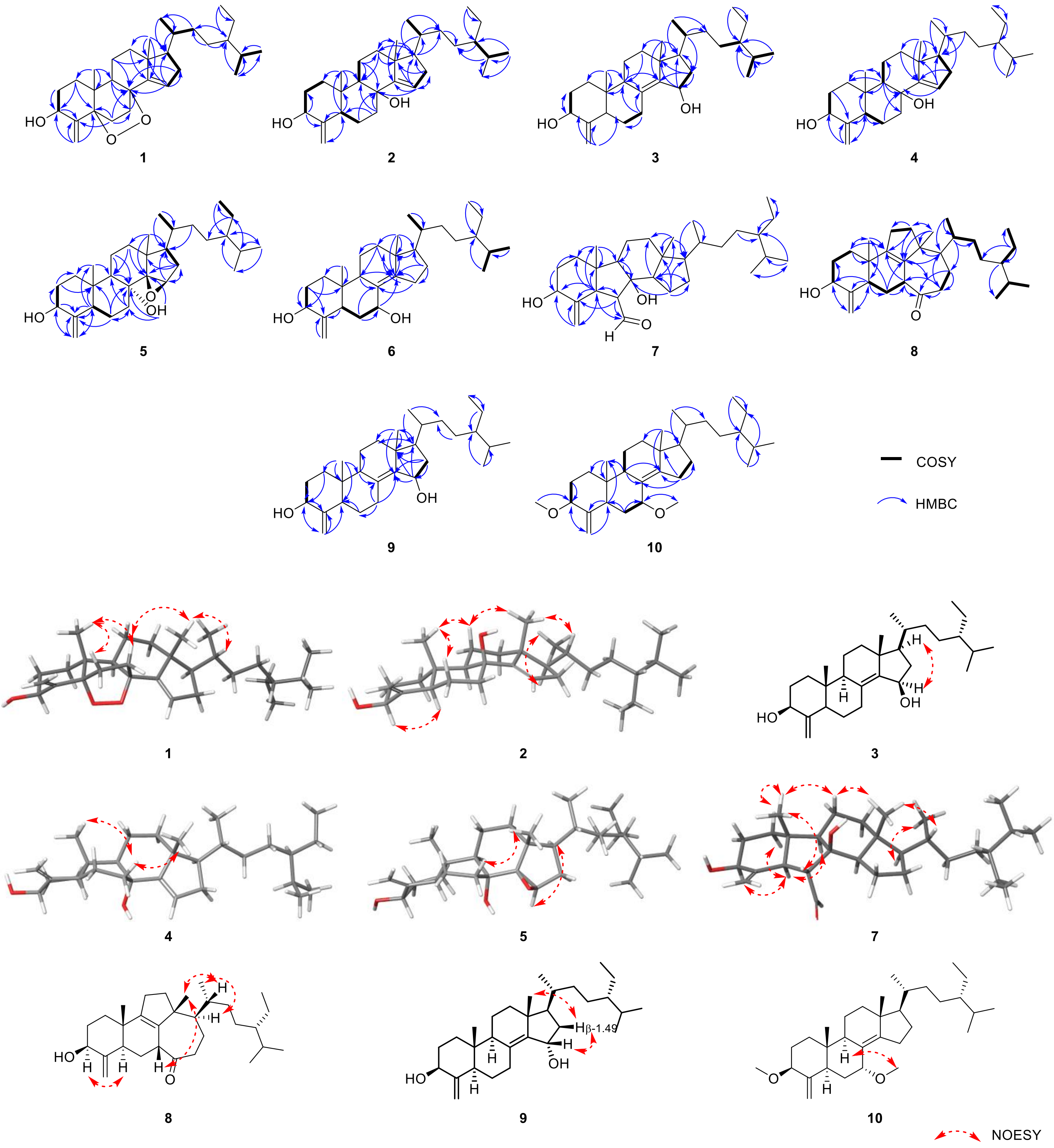

Figure S3. Key COSY, HMBC, and NOESY correlations of compounds **1-10**.

II. Computational methods

1. Geometry optimization

All molecular mechanics calculations were performed on Macromodel software (Maestro Materials Science 3.7.013 based on Maestro Core 12.3.013, MMshare Version 4.9.013, Release 2020-1, Platform Windows-x64). All conformational searches were conducted using the Polak-Ribier Conjugate Gradient (PRCG) method, the mixed torsional/low-mode sampling method, and the Optimized Potentials for Liquid Simulation (OPLS3e) force field. Also, the searches were carried out in the gas phase with a 21.0 kJ/mol upper energy limit. The maximum number of steps was set as 1000 to find all of the possible conformers.

Quantum mechanical calculations were performed using Jaguar (version 10.7). The conformers within an energy threshold of 5.0 kJ/mol were used as input structures for DFT calculations employing the mPW91PW1 and the 6-3lG* basis set to output optimized structures, gas phase energies, and Gibbs free energies. Vibrational frequencies computation was implemented at the same level. All optimization was performed “fine” grid density and “ultrafine” accuracy level.

2. Transition state for transformation of compound **4** to **9**

The reactant and product were optimized by DFT calculations at the MPW91PW1/6-31G* basis set. Linear Synchronous Transit (LST) method in Jaguar was used for finding transition state geometry at the same level in the optimization method. All optimization was performed fine grid density and ultrafine accuracy level.

(a)

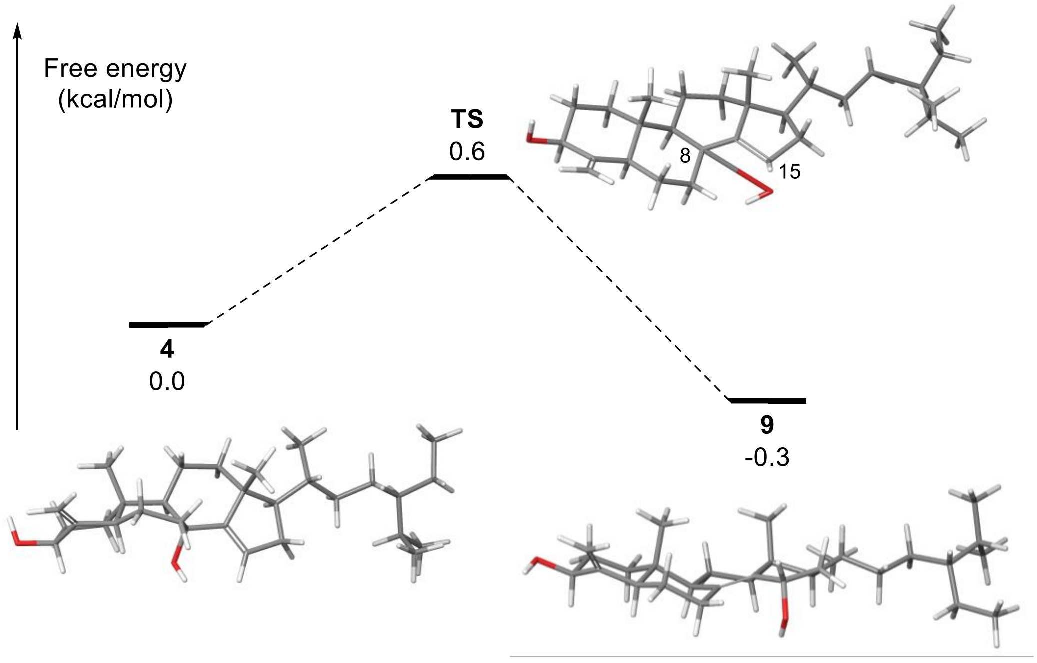

(b)

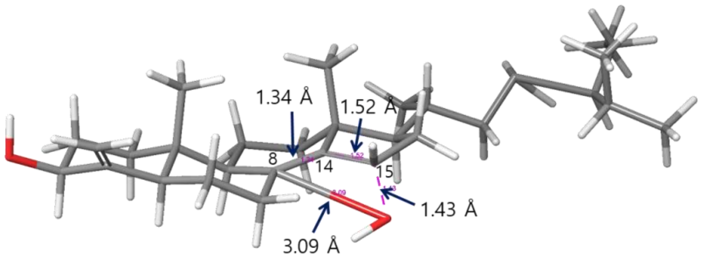

Figure S4. (a) Free energy diagram for 1,3-hydroxyl migration in 8α-hydroxy-theonellasterol **4** to generate 15α-hydroxy-theonellasterol **9**. (b) Bond lengths for C-8–C-14 and C-14–C15 and atomic distance between C-15 and O-8 in the TS structure.

Table S3. Parameters of optimized structures for compounds **4**, **9**, and **TS** in the gas phase.

	Gas phase energy (hartree)	[a] Gibbs free energy (kcal/mol)	Lowest frequency (cm^{-1})
8α-hydroxy-theonellasterol **4**	-1323.437806	-36.095072	27.968924
TS	-1323.277687	-36.674061	-445.948191
15α-hydroxy-theonellasterol **9**	-1323.456276	-36.394132	25.953253

[a] 298.15 K 1.0 atm

3. ^{13}C chemical shift calculations

Among the optimized structures, the structure that has the lowest gas phase energy was selected. For calculating NMR shielding constant with the GIAO method, single point energy calculations were conducted using the mPW91PW1/6-3lG** basis set with CPCM benzene solvent model. All optimization was performed “fine” grid density and “ultrafine” accuracy level. The calculated values of chemical shifts of compound **1-9** were calibrated to the TMS chemical shift computed with the same level of theory.

Table S4. The optimized structures of isomers **I–IV** used for ^{13}C chemical shift calculations.

Structure	Optimized geometry
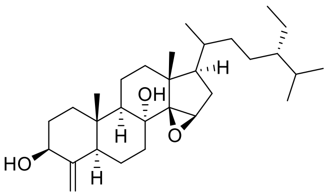 **5-I** (8-α-hydroxy-14,15-β-epoxide)	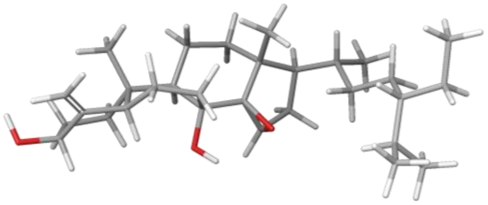
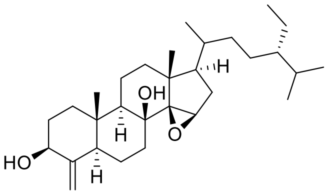 **5-II** (8-β-hydroxy-14,15-β-epoxide)	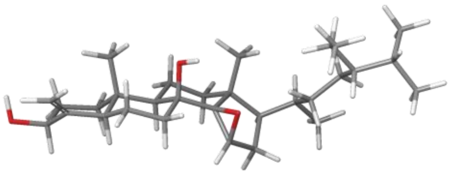
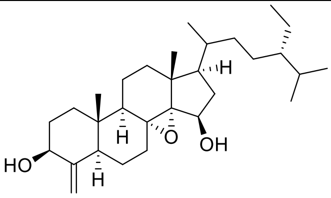 **5-III** (8,14-α-epoxy-15-β-alcohol)	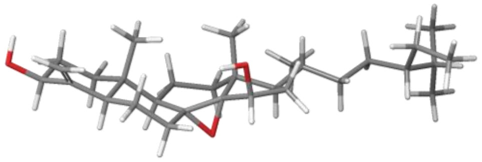
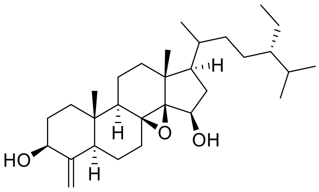 **5-IV** (8,14-β-epoxy-15-β-alcohol)	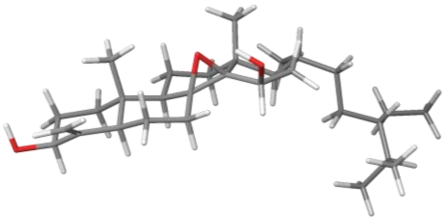

Table S5. Parameters of optimized isomers **I–IV** in the gas phase.

	Gas phase energy (hartree)	[a] Gibbs free energy (kcal/mol)
**5-I**	-1398.632101	-36.093924
**5-II**	-1398.721551	-36.192753
**5-III**	-1398.639529	-36.532159
**5-IV**	-1398.654758	-36.522056

[a] 298.15 K 1.0 atm

Table S6. Comparison between experimental and calculated ^{13}C chemical shifts in C_6D_6 for isomers **I–IV**.

(unit : ppm)

		**5-I**		**5-II**		**5-III**		**5-IV**	
no.	δ_{exptl}	δ_{calcd}	$\lvert\Delta\delta\rvert$	δ_{calcd}	$\lvert\Delta\delta\rvert$	δ_{calcd}	$\lvert\Delta\delta\rvert$	δ_{calcd}	$\lvert\Delta\delta\rvert$
1	41.5	42.25	0.75	39.01	2.49	37.7	3.8	38.2	3.3
2	33.3	35.63	2.33	35.10	1.8	34.8	1.5	35.1	1.8
3	73.8	72.19	1.61	71.80	2	72.1	1.7	71.8	2.0
4	154.3	153.10	1.2	151.97	2.33	152.2	2.1	152.0	2.3
5	41.2	42.08	0.88	51.21	10.01	49.2	8.0	50.3	9.1
6	20.3	21.26	0.96	22.73	2.43	25.4	5.1	24.6	4.3
7	28.3	31.72	3.42	35.50	7.2	31.9	3.5	30.9	2.6
8	71.3	72.36	1.06	74.21	2.91	63.5	7.8	65.0	6.3
9	55.3	51.58	3.72	55.13	0.17	47.2	8.1	50.1	5.2
10	38.2	39.24	1.04	41.51	3.31	42.1	3.9	41.7	3.5
11	23.3	26.10	2.8	21.60	1.7	21.7	1.6	20.0	3.3
12	34.7	41.40	6.7	42.38	7.68	33.6	1.1	38.5	3.8
13	42.5	45.78	3.28	47.34	4.84	43.4	0.9	42.5	0.0
14	75.9	76.40	0.5	75.16	0.74	80.0	4.1	72.1	3.9
15	58.8	60.35	1.55	62.52	3.72	74.0	15.2	65.9	7.1
16	32.3	25.79	6.51	27.61	4.69	38.8	6.5	38.8	6.5
17	49.8	55.36	5.56	54.48	4.68	54.6	4.8	48.5	1.3
18	15.0	18.87	3.87	20.08	5.08	16.2	1.2	16.8	1.8
19	17.2	21.26	4.06	16.48	0.72	17.2	0.0	15.6	1.7
20	34.4	36.65	2.25	35.22	0.82	38.5	4.1	34.5	0.1
21	19.2	21.54	2.34	23.04	3.84	19.2	0.0	20.8	1.6
22	34.1	31.51	2.59	31.93	2.17	35.7	1.6	36.0	1.8
23	27.1	31.06	3.96	29.34	2.24	28.7	1.6	22.6	4.5
24	46.8	47.34	0.54	45.79	1.01	45.3	1.5	45.5	1.3
25	29.6	28.88	0.72	26.12	3.48	28.6	1.0	28.0	1.6
26	19.4	15.36	4.04	14.53	4.87	16.6	2.8	15.8	3.6
27	20.1	22.67	2.57	28.60	8.5	22.8	2.7	22.5	2.4
28	23.7	22.77	0.93	22.79	0.91	25.8	2.1	25.8	2.1
29	12.9	14.70	1.8	16.24	3.34	14.2	1.3	15.2	2.3
30	103.4	102.36	1.04	103.13	0.27	104.8	1.4	104.1	0.7

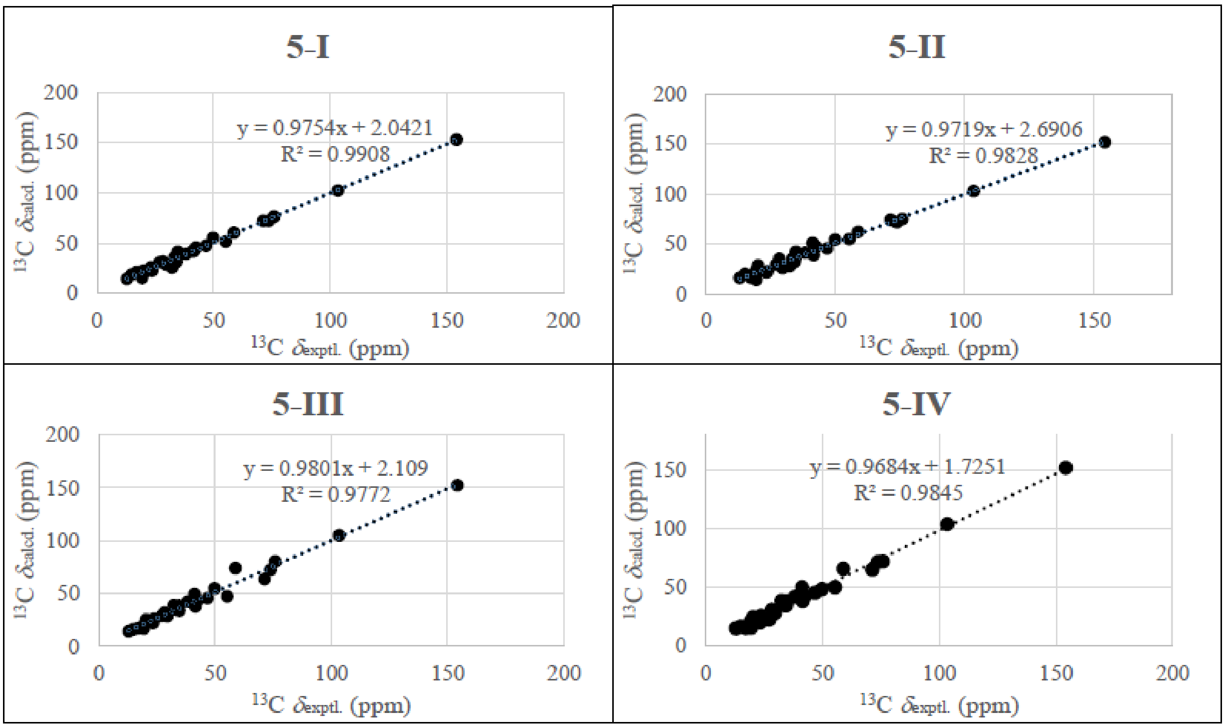

Figure S5. Linear regression analyses of the experimental and calculated ^{13}C NMR data for isomers **I-IV**.

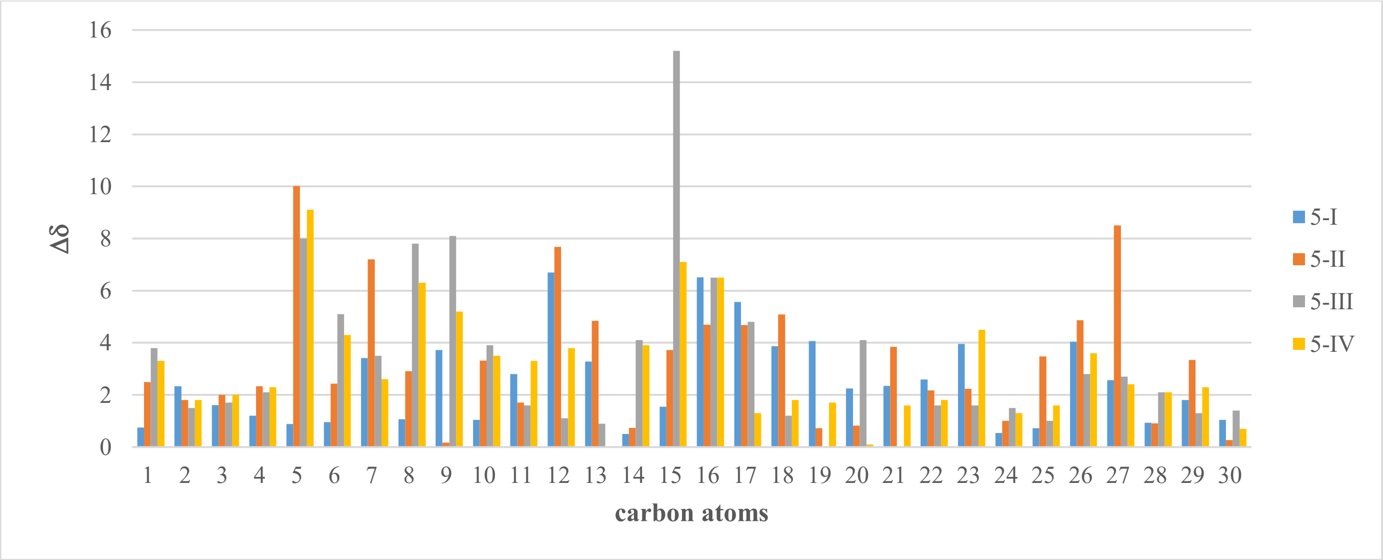

Figure S6. Comparison of $|\delta_{exptl} - \delta_{calcd}|$ values (parts per million) in C_6D_6 for isomers **I-IV**.

Functional	Solvent?	Basis Set	Type of Data
mPW1PW91	PCM	6-31G(d,p)	Shielding Tensors

		DP4+	100.00%	0.00%	0.00%	0.00%	-
**Nuclei**	**sp2?**	**Experimental**	**Isomer 1**	**Isomer 2**	**Isomer 3**	**Isomer 4**	**Isomer 5**
C	x	41.50	42.3	39.0	37.68	38.19	
C	x	33.30	35.6	35.1	34.80	35.10	
C	x	73.80	72.2	71.8	72.12	71.79	
C		154.30	153.1	152.0	152.20	151.99	
C	x	41.20	42.1	51.2	49.15	50.25	
C	x	20.30	21.3	22.7	25.42	24.57	
C	x	28.30	31.7	35.5	31.85	30.94	
C	x	71.30	72.4	74.2	63.49	65.03	
C	x	55.30	51.6	55.1	47.22	50.09	
C	x	38.20	39.2	41.5	42.13	41.73	
C	x	23.30	26.1	21.6	21.69	20.02	
C	x	34.70	41.40	42.38	33.60	38.47	
C	x	42.50	45.78	47.34	43.38	42.51	
C	x	75.90	76.40	75.16	80.04	72.05	
C	x	58.80	60.35	62.52	73.97	65.88	
C	x	32.30	25.79	27.61	38.75	38.81	
C	x	49.80	55.36	54.48	54.59	48.50	
C	x	15.00	18.87	20.08	16.17	16.82	
C	x	17.20	21.26	16.48	17.24	15.55	
C	x	34.40	36.65	35.22	38.49	34.49	
C	x	19.20	21.54	23.04	19.15	20.81	
C	x	34.10	31.51	31.93	35.66	35.95	
C	x	27.10	31.06	29.34	28.68	22.61	
C	x	46.80	47.34	45.79	45.30	45.54	
C	x	29.60	28.88	26.12	28.57	28.04	
C	x	19.40	15.36	14.53	16.58	15.80	
C	x	20.10	22.67	28.6	22.82	22.51	
C	x	23.70	22.77	22.79	25.82	25.80	
C	x	12.90	14.7	16.24	14.21	15.18	
C		103.40	102.36	103.13	104.80	104.08	

Functional	Solvent?	Basis Set	Type of Data
mPW1PW91	PCM	6-31G(d,p)	Shielding Tensors

	Isomer 1	Isomer 2	Isomer 3	Isomer 4	Isomer 5	Isomer 6
sDP4+ (H data)	-	-	-	-	-	-
sDP4+ (C data)	100.00%	0.00%	0.00%	0.00%	-	-
sDP4+ (all data)	100.00%	0.00%	0.00%	0.00%	-	-
uDP4+ (H data)	-	-	-	-	-	-
uDP4+ (C data)	32.86%	43.92%	17.39%	5.83%	-	-
uDP4+ (all data)	32.86%	43.92%	17.39%	5.83%	-	-
DP4+ (H data)	-	-	-	-	-	-
DP4+ (C data)	100.00%	0.00%	0.00%	0.00%	-	-
DP4+ (all data)	100.00%	0.00%	0.00%	0.00%	-	-

Figure S7. DP4+ probability analyses for isomers **I-IV**.

Table S7. Optimized Z-matrixes of isomer **5-I** in the gas phase (Å).

atom	x	y	z	atom	x	y	z	atom	x	y	z
C1	-1.06604	-6.97905	-0.10914	C29	-8.97037	0.611178	-0.8433	H57	-1.21454	-9.27662	-1.55034
C2	-0.46232	-8.16911	0.589947	C30	-5.28827	1.123875	1.535174	H58	-2.6741	-8.42799	-2.00483
C3	-0.93544	-7.11887	-1.65795	C31	-4.766	2.438005	-0.5437	H59	1.201783	-10.2256	0.531424
C4	0.575941	-7.17898	-1.95545	O32	-2.57955	-2.33428	-1.4535	H60	-0.58367	-9.79762	1.922413
C5	1.289381	-8.31125	-1.21359	O33	-0.71293	-4.23333	-0.85158	H61	-2.16814	-8.89337	1.619234
C6	1.018695	-8.31995	0.298464	H34	-0.42835	-6.12794	0.159015	H62	-5.87779	-2.04073	-3.80168
C7	-2.48278	-6.59331	0.292029	H35	0.738987	-7.29237	-3.03529	H63	-4.80868	-1.44962	-1.61705
C8	-2.84983	-5.2029	-0.26173	H36	1.017724	-6.21574	-1.67225	H64	-4.0324	-0.0901	-2.36662
C9	-1.93226	-4.72542	-1.40897	H37	0.973856	-9.28133	-1.62056	H65	-5.07527	-0.99364	-5.89908
C10	-1.55649	-5.85917	-2.38247	H38	2.371193	-8.25171	-1.37156	H66	-5.90787	0.170666	-4.86641
C11	-2.57367	-3.57241	-2.18125	H39	1.529351	-7.45483	0.742044	H67	-4.1469	0.190412	-4.96947
C12	-3.72748	-3.77627	-3.16621	H40	-3.19152	-7.34576	-0.07282	H68	-6.1406	1.056807	-2.72892
C13	-3.22178	-4.91742	-4.07892	H41	-2.57695	-6.59408	1.382484	H69	-7.05468	-0.41965	-2.51876
C14	-2.74825	-6.15276	-3.3111	H42	-3.89312	-5.19587	-0.57958	H70	-6.58494	-0.50171	-0.15178
C15	-3.78376	-2.44565	-4.00976	H43	-2.78061	-4.44588	0.527261	H71	-4.32357	0.380978	-0.19775
C16	-2.37749	-1.81152	-3.86503	H44	-0.76774	-5.4338	-3.01815	H72	-7.73127	1.640431	0.563562
C17	-1.77102	-2.43749	-2.64135	H45	-4.01976	-5.19737	-4.77807	H73	-7.57272	2.231866	-1.07775
C18	-5.08617	-4.16388	-2.57845	H46	-2.38963	-4.54456	-4.69133	H74	-9.02692	0.362765	-1.90713
C19	-1.58841	-8.42931	-2.12726	H47	-2.46536	-6.91974	-4.03729	H75	-9.83211	1.245238	-0.61262
O20	1.617662	-9.44077	0.913969	H48	-3.58361	-6.57633	-2.74163	H76	-9.0852	-0.32064	-0.27843
C21	-1.10909	-8.99913	1.409164	H49	-3.91458	-2.7625	-5.05242	H77	-4.33378	1.400557	1.995036
C22	-4.94997	-1.45388	-3.76843	H50	-1.7401	-2.04712	-4.72454	H78	-6.02783	1.863844	1.858927
C23	-4.92781	-0.72014	-2.42172	H51	-2.40959	-0.72023	-3.79133	H79	-5.58517	0.152365	1.945369
C24	-5.02314	-0.46762	-4.93959	H52	-0.70161	-2.38857	-2.46209	H80	-5.53076	3.191412	-0.32652
C25	-6.1754	0.124589	-2.1503	H53	-5.85239	-4.12988	-3.35958	H81	-3.83035	2.784808	-0.09287
C26	-6.40382	0.456605	-0.66537	H54	-5.09039	-5.18388	-2.18823	H82	-4.61892	2.414832	-1.62815
C27	-5.15371	1.066996	0.011992	H55	-5.39887	-3.49701	-1.772	H83	-0.96242	-3.42187	-0.38006
C28	-7.66498	1.315399	-0.48081	H56	-1.37326	-8.62675	-3.18246				

Table S8. Optimized Z-matrixes isomer **5-II** in the gas phase (Å).

atom	x	y	z	atom	x	y	z	atom	x	y	z
C1	-1.06604	-6.97905	-0.10914	C29	1.914596	-1.74323	2.319544	H57	-5.06173	-1.56256	-2.9246
C2	-0.46232	-8.16911	0.589947	C30	2.91998	2.089219	3.344305	H58	-5.93963	-0.25095	-3.7117
C3	-0.93544	-7.11887	-1.65795	C31	4.909861	1.466901	1.940267	H59	-7.49744	-3.47804	-7.06977
C4	0.575941	-7.17898	-1.95545	O32	0.041082	-0.86211	-2.51264	H60	-6.29371	-5.10213	-6.01333
C5	1.289381	-8.31125	-1.21359	O33	-2.95835	-1.21834	-2.26276	H61	-5.05675	-5.02142	-4.64235
C6	1.018695	-8.31995	0.298464	H34	-3.24662	-2.39678	-6.25471	H62	0.306649	2.895176	-1.11679
C7	-2.48278	-6.59331	0.292029	H35	-5.39095	0.511948	-5.98473	H63	1.032497	0.397814	-0.91646
C8	-2.84983	-5.2029	-0.26173	H36	-4.20813	-0.33778	-6.96763	H64	2.597951	0.928876	-1.46306
C9	-1.93226	-4.72542	-1.40897	H37	-6.99827	-1.33197	-6.19291	H65	2.778375	3.012868	-2.90855
C10	-1.55649	-5.85917	-2.38247	H38	-6.47969	-0.8112	-7.78831	H66	1.571766	4.287956	-2.6907
C11	-2.57367	-3.57241	-2.18125	H39	-4.9748	-2.7052	-8.0012	H67	2.615361	3.817916	-1.34685
C12	-3.72748	-3.77627	-3.16621	H40	-3.71833	-3.42785	-3.42846	H68	2.80966	2.499022	0.426278
C13	-3.22178	-4.91742	-4.07892	H41	-2.82957	-4.27667	-4.68992	H69	1.190806	2.117446	0.975707
C14	-2.74825	-6.15276	-3.3111	H42	-1.25491	-2.96945	-3.26162	H70	3.245672	-0.09118	0.656591
C15	-3.78376	-2.44565	-4.00976	H43	-1.26808	-2.3781	-4.91747	H71	3.739331	0.13593	3.106526
C16	-2.37749	-1.81152	-3.86503	H44	-2.53201	-0.23834	-5.40931	H72	0.933845	0.155984	2.622582
C17	-1.77102	-2.43749	-2.64135	H45	-2.40778	2.905822	-3.52264	H73	0.75587	-0.50576	1.014606
C18	-5.08617	-4.16388	-2.57845	H46	-1.67453	1.958465	-4.80847	H74	2.590701	-1.68319	3.177995
C19	-1.58841	-8.42931	-2.12726	H47	-4.10178	1.598347	-4.734	H75	1.049976	-2.33863	2.629507
O20	1.617662	-9.44077	0.913969	H48	-4.0225	1.030031	-3.07615	H76	2.431934	-2.29761	1.528854
C21	-1.10909	-8.99913	1.409164	H49	0.055352	2.830423	-3.59024	H77	2.766693	3.019066	2.786379
C22	-4.94997	-1.45388	-3.76843	H50	1.017093	1.254019	-4.96994	H78	1.951616	1.78101	3.750357
C23	-4.92781	-0.72014	-2.42172	H51	2.068618	0.828868	-3.62854	H79	3.571823	2.321706	4.192824
C24	-5.02314	-0.46762	-4.93959	H52	0.499932	-1.16918	-4.5656	H80	4.82006	2.343802	1.289651
C25	-6.1754	0.124589	-2.1503	H53	-1.10491	0.964728	-0.65059	H81	5.574675	1.742475	2.765896
C26	-6.40382	0.456605	-0.66537	H54	-1.83766	2.495402	-1.14787	H82	5.402078	0.675303	1.365237
C27	-5.15371	1.066996	0.011992	H55	-2.7396	0.982662	-1.30789	H83	-2.31692	-1.55764	-1.62192
C28	-7.66498	1.315399	-0.48081	H56	-6.2783	-1.92706	-4.15371				

Table S9. Optimized Z-matrixes of **5-III** in the gas phase (Å).

atom	x	y	z	atom	x	y	z	atom	x	y	z
C1	-10.0697	-1.95292	-1.46445	C29	-11.5994	9.728055	6.630673	H57	-11.535	-1.52001	0.632501
C2	-9.89427	-0.48297	-1.14437	C30	-10.1641	7.68819	7.230288	H58	-11.1131	-0.03643	1.481335
C3	-11.2354	0.180393	-0.69859	C31	-8.96561	8.387334	7.864986	H59	-11.6448	-4.02371	-2.02054
C4	-12.2846	-0.10029	-1.79158	O32	-12.8933	9.397181	7.376716	H60	-9.51662	-3.969	-1.22162
C5	-12.4466	-1.58243	-2.11677	O33	-11.8678	10.73835	5.513815	H61	-8.62983	-2.77024	-0.13267
C6	-11.1213	-2.23438	-2.51769	H34	-9.61902	0.007016	-2.09488	H62	-8.68314	1.185052	2.968071
C7	-8.7742	-0.18901	-0.15264	H35	-13.2536	0.314637	-1.4941	H63	-11.9836	4.858235	4.444511
C8	-8.55687	1.312837	-0.02924	H36	-11.9884	0.433517	-2.70593	H64	-10.0566	6.352826	4.724125
C9	-9.81674	2.112904	0.247916	H37	-12.8464	-2.11801	-1.24451	H65	-10.6273	7.397681	3.439645
C10	-11.0362	1.726234	-0.58962	H38	-13.1682	-1.72201	-2.92828	H66	-13.6319	6.583493	3.661605
C11	-9.982	2.933963	1.461089	H39	-10.7876	-1.7837	-3.46232	H67	-12.8155	6.64176	2.09377
C12	-11.3226	3.500109	1.931492	H40	-9.0107	-0.63285	0.821804	H68	-13.6319	5.166704	2.62497
C13	-12.1761	3.734407	0.676647	H41	-7.84472	-0.65994	-0.4884	H69	-12.5175	8.138138	4.750884
C14	-12.3338	2.459434	-0.16634	H42	-7.78924	1.54266	0.716235	H70	-12.3007	6.87083	5.941873
C15	-10.7924	4.781378	2.655872	H43	-8.1637	1.689552	-0.98293	H71	-10.0603	8.848601	5.439419
C16	-9.49608	4.295652	3.360259	H44	-10.7733	2.065697	-1.60322	H72	-10.9282	10.22291	7.344965
C17	-8.97973	3.069699	2.593267	H45	-13.1795	4.073078	0.949676	H73	-10.9005	7.440348	8.005678
C18	-9.53848	3.520571	0.22745	H46	-11.7204	4.52705	0.077829	H74	-9.82788	6.724201	6.829122
C19	-12.0257	2.564668	2.924197	H47	-12.905	2.712222	-1.06561	H75	-8.49822	7.75072	8.62274
O20	-11.6812	-0.43657	0.63739	H48	-12.9692	1.767775	0.394313	H76	-8.20345	8.621451	7.113462
C21	-11.2877	-3.60364	-2.81524	H49	-10.5014	5.48164	1.859402	H77	-9.24372	9.324769	8.356184
C22	-9.37599	-2.94278	-0.89999	H50	-8.73997	5.082036	3.405937	H78	-13.6409	8.95856	6.707199
C23	-8.97275	1.955024	3.474446	H51	-9.70239	3.985621	4.390409	H79	-12.7285	8.693549	8.198462
C24	-11.7223	5.540477	3.621675	H52	-7.97824	3.240895	2.180312	H80	-13.332	10.30415	7.80541
C25	-10.9665	6.728606	4.244005	H53	-12.9594	3.014746	3.276474	H81	-12.5407	10.33293	4.750135
C26	-13.0206	6.005916	2.963037	H54	-12.2795	1.607198	2.464792	H82	-12.3371	11.64461	5.911185
C27	-11.7546	7.547705	5.269322	H55	-11.3999	2.347243	3.792073	H83	-10.9391	11.03504	5.014829
C28	-10.8552	8.477319	6.105763	H56	-12.7405	-0.25324	0.839679				

Table S10. Optimized Z-matrixes of **5-IV** in the gas phase (Å).

atom	x	y	z	atom	x	y	z	atom	x	y	z
C1	-5.81275	2.70178	-4.70469	C29	-12.2709	3.203873	-14.9819	H57	-4.06958	1.158988	-5.98824
C2	-6.4018	2.530672	-6.08748	C30	-10.4452	3.156389	-16.7806	H58	-4.87745	0.365273	-7.34249
C3	-5.29169	2.511919	-7.18723	C31	-11.2921	2.890218	-18.0214	H59	-3.78766	3.402555	-3.13155
C4	-4.47011	3.806516	-7.03244	O32	-12.0297	4.582018	-14.3629	H60	-5.58667	2.075825	-2.70766
C5	-3.89624	4.001698	-5.63165	O33	-13.0465	2.309832	-14.0129	H61	-6.57994	0.949525	-3.78348
C6	-4.97556	3.95495	-4.54745	H34	-6.98313	3.450351	-6.28102	H62	-8.00257	-1.23578	-9.22986
C7	-7.37569	1.362639	-6.22343	H35	-6.60052	3.435678	-8.60341	H63	-7.07262	0.397729	-14.0416
C8	-8.04777	1.365189	-7.59492	H36	-8.374	2.279712	-12.0397	H64	-8.61033	1.252891	-15.6864
C9	-7.01702	1.392265	-8.69762	H37	-3.64961	3.818342	-7.7573	H65	-9.43479	0.361711	-14.4321
C10	-5.99753	2.51267	-8.57276	H38	-5.11273	4.664691	-7.27785	H66	-7.06214	3.456353	-13.9066
C11	-7.30929	0.802782	-10.0143	H39	-3.15755	3.217168	-5.41797	H67	-5.67286	2.370945	-13.9331
C12	-6.63323	1.236679	-11.3245	H40	-3.36689	4.957761	-5.56291	H68	-6.6812	2.535586	-15.3658
C13	-5.87206	2.552881	-11.0955	H41	-5.63511	4.823576	-4.68005	H69	-10.3125	2.381772	-13.4195
C14	-5.07228	2.557355	-9.79007	H42	-6.85128	0.411943	-6.07623	H70	-9.32219	3.418427	-14.4254
C15	-7.86167	1.336771	-12.288	H43	-8.1384	1.425062	-5.44065	H71	-11.2511	1.480268	-15.7264
C16	-8.76507	0.176601	-11.8352	H44	-8.68157	2.257134	-7.68991	H72	-12.9164	3.349177	-15.8582
C17	-8.68348	0.184658	-10.3103	H45	-8.69654	0.493052	-7.71217	H73	-10.335	4.238644	-16.6338
C18	-6.50407	0.088411	-9.06845	H46	-5.1942	2.74876	-11.9311	H74	-9.43128	2.78263	-16.9683
C19	-5.66266	0.133682	-11.7696	H47	-6.58658	3.387532	-11.0738	H75	-10.8405	3.345105	-18.9086
O20	-4.38128	1.285469	-7.02856	H48	-4.4616	3.464515	-9.75381	H76	-11.3845	1.815155	-18.211
C21	-4.41988	4.122931	-3.26125	H49	-4.37787	1.711416	-9.77114	H77	-12.3038	3.296764	-17.9284
C22	-5.9967	1.858353	-3.68802	H50	-9.79443	0.273056	-12.1889	H78	-11.4494	4.509457	-13.4368
C23	-8.83242	-1.08067	-9.7119	H51	-8.38354	-0.78159	-12.2056	H79	-11.4928	5.252421	-15.041
C24	-7.59671	1.333904	-13.8048	H52	-9.46457	0.850758	-9.91525	H80	-12.9814	5.062663	-14.1138
C25	-8.89989	1.298706	-14.6285	H53	-5.14772	0.417471	-12.6923	H81	-12.4795	2.113743	-13.0961
C26	-6.70478	2.488065	-14.2718	H54	-4.90917	-0.05144	-11.0015	H82	-13.9894	2.781076	-13.7157
C27	-9.86757	2.465938	-14.4182	H55	-6.17603	-0.8154	-11.9478	H83	-13.2861	1.343298	-14.4687
C28	-10.9773	2.519685	-15.4855	H56	-3.47695	1.39037	-7.63646				

III. Determination of the C-24 configuration

1. The list of compounds selected for a database

(1) (21*R*,24*S*)-set

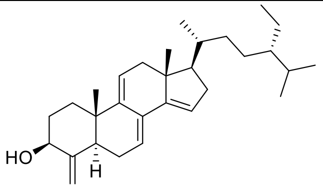
Theonellasterol B

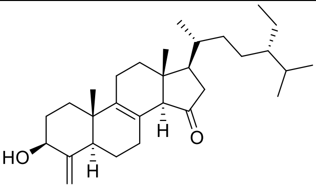
Theonellasterol C

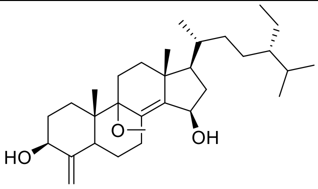
Theonellasterol D

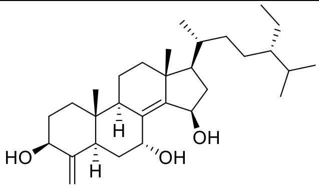
Theonellasterol F

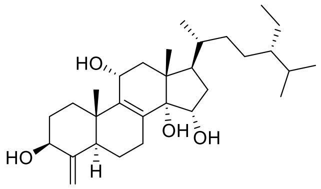
Theonellasterol H

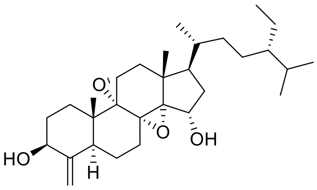
Theonellasterol I

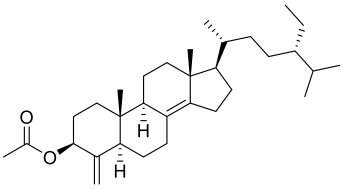
Acetyltheonellasterol

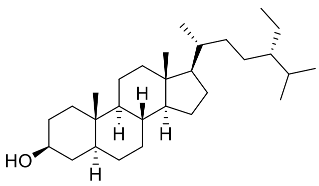
5*a*-Poriferast-9(11)-en-3*ß*-ol

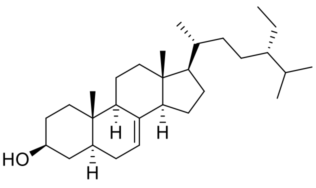
22-Dihydrochondrillasterol

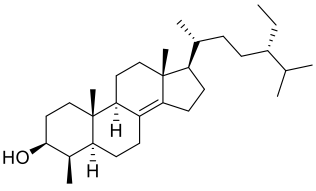
17

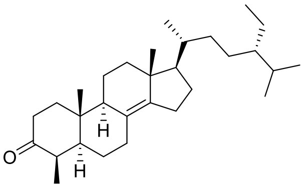
18

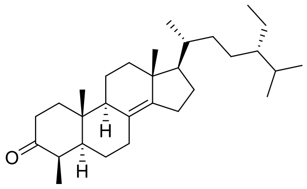
19

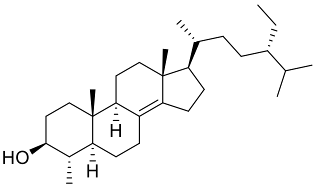
20

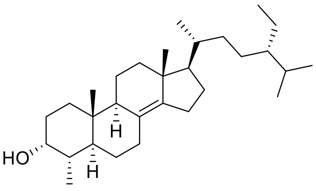
21

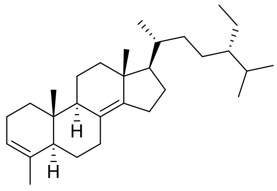
22

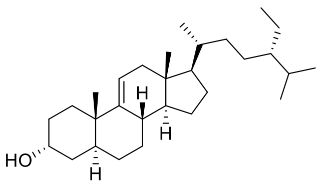
Graecumsterol

(2) (21*R*,24*R*)-set

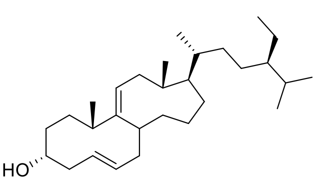
Cucumidisecosterol

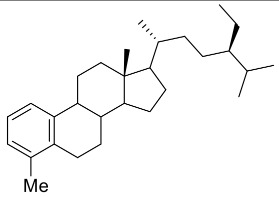
23

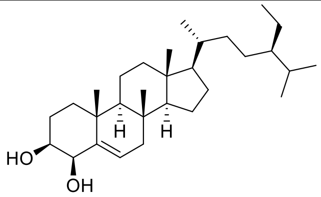
24

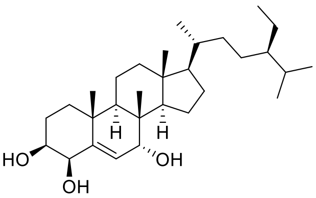
25

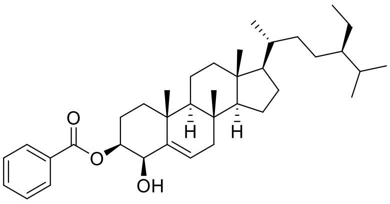
26

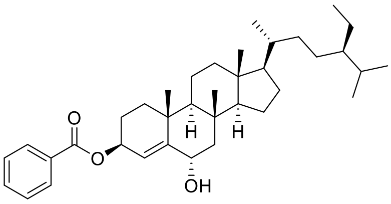
27

2. Calculation of $|\delta_{H-26}-\delta_{H-27}|$ values from the selected compounds

Table S11. $|\delta_{H-26}-\delta_{H-27}|$ values for the (21*R*, 24*S*) and (21*R*, 24*R*)-sets.

(21*R*,24*S*)-group							
Compound	δ_{H-26}	δ_{H-27}	$\|\delta_{H-26}-\delta_{H-27}\|$	Compound	δ_{H-26}	δ_{H-27}	$\|\delta_{H-26}-\delta_{H-27}\|$
Theonellasterol A[1]	0.90	0.92	0.02	Acetyltheonellasterol[7]	0.82	0.83	0.01
Theonellasterol B[2]	0.90	0.92	0.02	5α-poriferast-9(11)-en-3β-ol[3]	0.81	0.82	0.01
Theonellasterol C[2]	0.91	0.93	0.02	22-dihydrochondrillasterol[4]	0.81	0.83	0.02
Theonellasterol D[2]	0.90	0.92	0.02	**17**[5]	0.91	0.90	0.01
Theonellasterol E[2]	0.93	0.94	0.01	**18**[5]	0.91	0.89	0.02
Theonellasterol F[2]	0.93	0.94	0.01	**19**[5]	0.91	0.89	0.02
Theonellasterol G[2]	0.91	0.94	0.03	**20**[5]	0.91	0.90	0.01
Theonellasterol H[2]	0.91	0.93	0.02	**21**[5]	0.91	0.89	0.02
Theonellasterol I[6]	0.89	0.91	0.02	**22**[5]	0.91	0.90	0.01
Theonellasterol K[7]	0.81	0.83	0.02	Graecumsterol[8]	0.83	0.81	0.02
(21*R*,24*R*)-group							
Compound	δ_{H-26}	δ_{H-27}	$\|\delta_{H-26}-\delta_{H-27}\|$	Compound	δ_{H-26}	δ_{H-27}	$\|\delta_{H-26}-\delta_{H-27}\|$
Cucumidisecosterol[9]	0.716	0.666	0.05	**25**[11]	0.79	0.91	0.12
**23**[10]	0.82	0.86	0.04	**26**[11]	0.92	0.97	0.05
**24**[11]	0.81	0.86	0.05	**27**[11]	0.86	0.91	0.05

Table S12. $|\delta_{H-26}-\delta_{H-27}|$ values for compounds **1-9.**

Compound	δ_{H-26}	δ_{H-27}	$\|\delta_{H-26}-\delta_{H-27}\|$	Compound	δ_{H-26}	δ_{H-27}	$\|\delta_{H-26}-\delta_{H-27}\|$
**1**	0.88	0.90	0.02	**6**	0.82	0.83	0.01
**2**	0.90	0.92	0.02	**7**	0.89	0.92	0.03
**3**	0.91	0.92	0.01	**8**	0.81	0.82	0.01
**4**	0.92	0.91	0.01	**9**	0.91	0.92	0.01
**5**	0.86	0.88	0.02				

1 Kho, E.; Imagawa, D. K.; Rohmer, M.; Kashman, Y.; Djerassi, C. *J. Org. Chem.* **1981**, *46*, 1836-1839.
2 De Marino, S.; Ummarino, R.; D'Auria, M. V.; Chini, M. G.; Bifulco, G.; Renga, B.; D'Amore, C.; Fiorucci, S.; Debitus, C.; Zampella, A. *J. Med. Chem.* **2011**, *54*, 3065-3075.
3 Das, B.; Venkateswarlu, Y.; Srinivas, K.; Rama Rao, A. *Phytochemistry.* **1992**, *31*, 1054-1055.
4 Tasdemir, D.; Topaloglu, B.; Perozzo, R.; Brun, R.; O'Neill, R.; Carballeira, N. M.; Zhang, X.; Tonge, P. J.; Linden, A.; Rüedi, P. *Bioorg. Med. Chem.* **2007**, *15*, 6834-6845.
5 Sepe, V.; Ummarino, R.; D'Auria, M. V.; Taglialatela-Scafati, O.; Marino, S. D.; D'Amore, C.; Renga, B.; Chini, M. G.; Bifulco, G.; Nakao, Y. *Mar. Drugs.* **2012**, *10*, 2448-2466.
6 Chini, M. G.; Jones, C. R.; Zampella, A.; D'Auria, M. V.; Renga, B.; Fiorucci, S.; Butts, C. P.; Bifulco, G. *J. Org. Chem.* **2012**, *77*, 1489-1496.
7 Guo, J.-K.; Chiang, C.-Y.; Lu, M.-C.; Chang, W.-B.; Su, J.-H. *Mar. Drugs.* **2012**, *10*, 1536-1544.
8 Abdelkarim, A. S.; Ali, M.; Naquvi, K. J. *IOSR J. Pharm.* **2013**, *3*, 46-52.
9 Al-Rehaily, A. J.; Al-Yahya, M. A.; Mirza, H. H.; Ahmed, B. *Pharm. Biol.* **2002**, *40*, 154-159.
10 Stoilov, I.; Shetty, R.; St. Pyrek, J.; Smith, S. L.; Layton, W. J.; Watt, D. S.; Carlson, R. M.; Moldowan, J. M. *J. Org. Chem.* **1994**, *59*, 926-928.
11 Ghosh, P.; Das, J.; Sarkar, A.; Ng, S. W.; Tiekink, E. R. *Tetrahedron.* **2012**, *68*, 6485-6491.

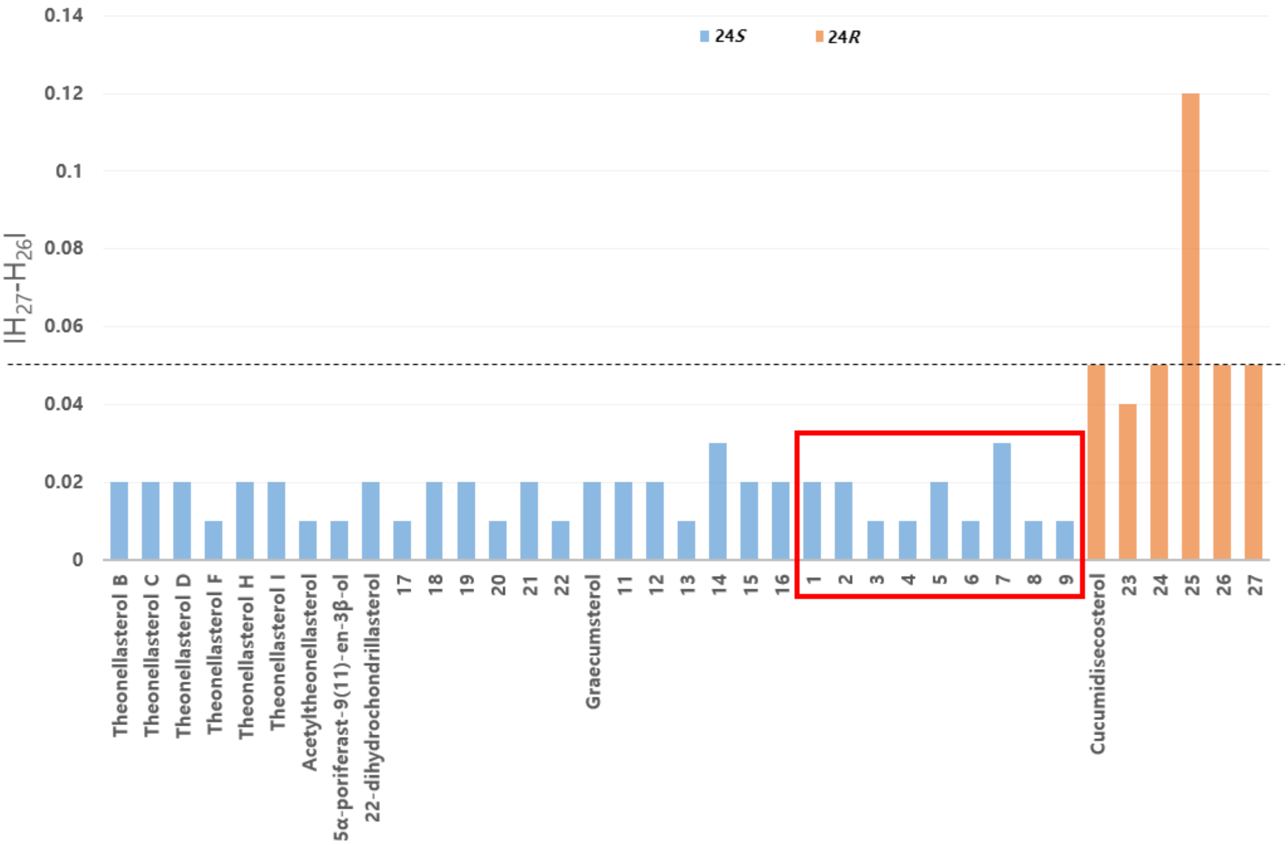

Figure S8. Comparison of $|\delta_{H\text{-}26}\text{-}\delta_{H\text{-}27}|$ values for (21*R*,24*S*)-set, (21*R*,24*R*)-set, and compounds **1-9**.

IV. X-ray crystallography data

Table S12. Crystal data and structure refinement for 7α-theonellasterol.

Identification code	7-alpha-theonellasterol
Empirical formula	C30 H50 O2
Formula weight	442.70
Temperature	223(2) K
Wavelength	1.54178 Å
Crystal system	Monoclinic
Space group	$P2_1$
Unit cell dimensions	a = 7.6741(10) Å
	b = 46.648(6) Å
	c = 8.1389(10) Å
Volume	2754.2(6) Å^3
Z	4
Density (calculated)	1.068 Mg/m^3
Absorption coefficient	0.485 mm^{-1}
F(000)	984
Crystal size	0.495 x 0.035 x 0.012 mm^3
Theta range for data collection	3.790 to 77.669°.
Index ranges	-9<=h<=9, -57<=k<=58, -9<=l<=10
Reflections collected	48446
Independent reflections	11015 [R(int) = 0.1461]
Completeness to theta = 67.679°	99.0 %
Absorption correction	Semi-empirical from equivalents
Max. and min. transmission	0.7541 and 0.6016
Refinement method	Full-matrix least-squares on F^2
Data / restraints / parameters	11015 / 1 / 593
Goodness-of-fit on F^2	1.053
Final R indices [I>2sigma(I)]	R1 = 0.0886, wR2 = 0.2369
R indices (all data)	R1 = 0.1567, wR2 = 0.2980
Absolute structure parameter	-0.1(3)
Extinction coefficient	n/a
Largest diff. peak and hole	0.221 and -0.222 e.Å^{-3}

Table S13. Atomic coordinates (x 10^4) and equivalent isotropic displacement parameters (Å ^{2} x 10^3) for 7α-theonellasterol.

	x	y	z	U (eq)		x	y	z	U (eq)
C(1)	6328(9)	4329(2)	12497(9)	94(3)	C(31)	-4381(9)	5700(2)	4684(8)	92(3)
C(2)	8312(10)	4436(3)	12840(10)	102(3)	C(32)	-4496(12)	5634(3)	2800(9)	122(4)
C(3)	9277(10)	4532(3)	14711(11)	110(3)	C(33)	-6398(12)	5536(3)	1658(9)	121(4)
C(4)	8192(10)	4751(3)	15292(9)	104(3)	C(34)	-6989(11)	5278(3)	2471(10)	108(3)
C(5)	6253(10)	4652(2)	15009(8)	92(2)	C(35)	-6962(10)	5347(2)	4302(9)	95(2)
C(6)	5253(9)	4562(2)	13117(8)	87(2)	C(36)	-5054(10)	5437(2)	5461(8)	90(2)
C(7)	3233(10)	4479(2)	12752(9)	99(3)	C(37)	-4908(10)	5482(2)	7372(8)	89(2)
C(8)	2270(10)	4424(3)	10800(10)	101(3)	C(38)	-2929(9)	5548(2)	8496(8)	90(3)
O(1)	9139(7)	4829(2)	17083(6)	121(2)	C(39)	-2109(10)	5792(2)	7756(8)	87(2)
C(9)	3315(9)	4205(2)	10087(10)	92(2)	C(40)	-2314(10)	5746(2)	5813(8)	95(3)
O(2)	2167(7)	4697(2)	9932(6)	106(2)	C(41)	-1347(13)	5983(3)	5117(11)	126(4)
C(10)	5353(10)	4285(2)	10506(9)	93(2)	C(42)	320(12)	6113(3)	6497(11)	119(4)
C(11)	6367(11)	4074(2)	9697(12)	111(3)	C(43)	-291(11)	6244(2)	7950(11)	106(3)
C(12)	5123(12)	3926(2)	8030(13)	107(3)	C(44)	-1265(10)	6016(2)	8648(10)	91(2)
C(13)	3508(11)	3769(2)	8336(13)	106(3)	C(45)	-950(11)	6073(2)	10593(9)	99(3)
C(14)	2550(11)	3976(2)	9194(11)	95(2)	C(46)	462(12)	6315(2)	11068(11)	106(3)
C(15)	502(11)	3898(3)	8660(12)	109(3)	C(47)	1378(11)	6319(2)	9665(11)	104(3)
C(16)	225(12)	3670(2)	7272(15)	116(3)	C(48)	-5519(12)	5968(3)	4746(11)	112(3)
C(17)	1912(12)	3687(2)	6655(13)	107(3)	O(3)	-8764(7)	5174(2)	1378(7)	118(2)
C(18)	6364(13)	4046(2)	13499(13)	115(3)	C(49)	-8443(11)	5325(3)	4775(12)	115(3)
C(19)	5470(11)	4648(3)	16236(9)	112(3)	O(4)	-1861(8)	5294(2)	8645(6)	105(2)
C(20)	4183(15)	3505(3)	9540(18)	143(4)	C(50)	-1491(14)	6513(3)	7371(15)	134(4)
C(21)	2120(14)	3415(3)	5604(17)	125(3)	C(51)	2496(14)	6591(3)	9662(15)	131(4)
C(22)	356(16)	3361(2)	4126(18)	135(4)	C(52)	3879(15)	6660(2)	11434(16)	130(4)
C(23)	354(15)	3070(2)	3133(19)	143(4)	C(53)	4924(18)	6944(3)	11590(20)	162(6)
C(24)	-1623(17)	2986(3)	2040(20)	147(5)	C(54)	6016(14)	7028(2)	13469(18)	134(4)
C(25)	-2647(18)	2858(3)	3250(20)	175(6)	C(55)	4630(30)	7138(5)	14290(30)	209(10)
C(26)	-4720(20)	2835(4)	2290(30)	219(9)	C(56)	5270(40)	7158(5)	16150(50)	292(17)
C(27)	3820(20)	3425(4)	5030(20)	183(7)	C(57)	3500(30)	6575(5)	8290(20)	228(10)
C(28)	-1615(16)	2783(3)	530(20)	146(5)	C(58)	7625(17)	7230(3)	13620(20)	165(6)

Table S14. Bond lengths [Å] and angles [°] for 7α-theonellasterol.

C(1)-C(2)	1.539(10)	C(13)-C(14)	1.516(13)	C(26)-H(26A)	0.9700	C(38)-O(4)	1.425(11)	C(51)-C(52)	1.520(16)
C(1)-C(18)	1.547(14)	C(13)-C(20)	1.553(16)	C(26)-H(26B)	0.9700	C(38)-C(39)	1.516(12)	C(51)-C(57)	1.55(2)
C(1)-C(6)	1.547(11)	C(13)-C(17)	1.557(13)	C(26)-H(26C)	0.9700	C(38)-H(38)	0.9900	C(51)-H(51)	0.9900
C(1)-C(10)	1.561(10)	C(14)-C(15)	1.532(11)	C(27)-H(27A)	0.9700	C(39)-C(44)	1.316(13)	C(52)-C(53)	1.532(16)
C(2)-C(3)	1.528(11)	C(15)-C(16)	1.517(15)	C(27)-H(27B)	0.9700	C(39)-C(40)	1.552(9)	C(52)-H(52A)	0.9800
C(2)-H(2A)	0.9800	C(15)-H(15A)	0.9800	C(27)-H(27C)	0.9700	C(40)-C(41)	1.539(14)	C(52)-H(52B)	0.9800
C(2)-H(2B)	0.9800	C(15)-H(15B)	0.9800	C(28)-C(29)	1.52(2)	C(40)-H(40)	0.9900	C(53)-C(54)	1.536(18)
C(3)-C(4)	1.490(12)	C(16)-C(17)	1.536(13)	C(28)-C(30)	1.555(18)	C(41)-C(42)	1.526(14)	C(53)-H(53A)	0.9800
C(3)-H(3A)	0.9800	C(16)-H(16A)	0.9800	C(28)-H(28)	0.9900	C(41)-H(41A)	0.9800	C(53)-H(53B)	0.9800
C(3)-H(3B)	0.9800	C(16)-H(16B)	0.9800	C(29)-H(29A)	0.9700	C(41)-H(41B)	0.9800	C(54)-C(55)	1.52(2)
C(4)-O(1)	1.447(9)	C(17)-C(21)	1.566(15)	C(29)-H(29B)	0.9700	C(42)-C(43)	1.535(13)	C(54)-C(58)	1.525(16)
C(4)-C(5)	1.502(11)	C(17)-H(17)	0.9900	C(29)-H(29C)	0.9700	C(42)-H(42A)	0.9800	C(54)-H(54)	0.9900
C(4)-H(4)	0.9900	C(18)-H(18A)	0.9700	C(30)-H(30A)	0.9700	C(42)-H(42B)	0.9800	C(55)-C(56)	1.43(3)
C(5)-C(19)	1.323(9)	C(18)-H(18B)	0.9700	C(30)-H(30B)	0.9700	C(43)-C(44)	1.514(13)	C(55)-H(55A)	0.9800
C(5)-C(6)	1.537(10)	C(18)-H(18C)	0.9700	C(30)-H(30C)	0.9700	C(43)-C(50)	1.536(15)	C(55)-H(55B)	0.9800
C(6)-C(7)	1.530(10)	C(19)-H(19A)	0.9400	C(31)-C(48)	1.536(14)	C(43)-C(47)	1.595(13)	C(56)-H(56A)	0.9700
C(6)-H(6)	0.9900	C(19)-H(19B)	0.9400	C(31)-C(32)	1.538(10)	C(44)-C(45)	1.545(11)	C(56)-H(56B)	0.9700
C(7)-C(8)	1.539(11)	C(20)-H(20A)	0.9700	C(31)-C(36)	1.544(13)	C(45)-C(46)	1.525(14)	C(56)-H(56C)	0.9700
C(7)-H(7A)	0.9800	C(20)-H(20B)	0.9700	C(31)-C(40)	1.567(10)	C(45)-H(45A)	0.9800	C(57)-H(57A)	0.9700
C(7)-H(7B)	0.9800	C(20)-H(20C)	0.9700	C(32)-C(33)	1.524(13)	C(45)-H(45B)	0.9800	C(57)-H(57B)	0.9700
C(8)-O(2)	1.447(12)	C(21)-C(22)	1.511(16)	C(32)-H(32A)	0.9800	C(46)-C(47)	1.524(13)	C(57)-H(57C)	0.9700
C(8)-C(9)	1.526(13)	C(21)-C(27)	1.523(16)	C(32)-H(32B)	0.9800	C(46)-H(46A)	0.9800	C(58)-C(59)	1.48(2)
C(8)-H(8)	0.9900	C(21)-H(21)	0.9900	C(33)-C(34)	1.512(16)	C(46)-H(46B)	0.9800	C(58)-C(60)	1.54(2)
O(1)-H(1)	0.8300	C(22)-C(23)	1.576(16)	C(33)-H(33A)	0.9800	C(47)-C(51)	1.531(15)	C(58)-H(58)	0.9900
C(9)-C(14)	1.314(13)	C(22)-H(22A)	0.9800	C(33)-H(33B)	0.9800	C(47)-H(47)	0.9900	C(59)-H(59A)	0.9700
C(9)-C(10)	1.535(10)	C(22)-H(22B)	0.9800	C(34)-O(3)	1.446(10)	C(48)-H(48A)	0.9700	C(59)-H(59B)	0.9700
O(2)-H(2)	0.8300	C(23)-C(24)	1.538(15)	C(34)-C(35)	1.518(10)	C(48)-H(48B)	0.9700	C(59)-H(59C)	0.9700
C(10)-C(11)	1.530(12)	C(23)-H(23A)	0.9800	C(34)-H(34)	0.9900	C(48)-H(48C)	0.9700	C(60)-H(60A)	0.9700
C(10)-H(10)	0.9900	C(23)-H(23B)	0.9800	C(35)-C(49)	1.317(11)	O(3)-H(3)	0.8300	C(60)-H(60B)	0.9700
C(11)-C(12)	1.544(12)	C(24)-C(28)	1.555(19)	C(35)-C(36)	1.520(11)	C(49)-H(49A)	0.9400	C(60)-H(60C)	0.9700
C(11)-H(11A)	0.9800	C(24)-C(25)	1.56(2)	C(36)-C(37)	1.537(9)	C(49)-H(49B)	0.9400	C(2)-C(1)-C(18)	109.5(7)
C(11)-H(11B)	0.9800	C(24)-H(24)	0.9900	C(36)-H(36)	0.9900	O(4)-H(4A)	0.8300	C(2)-C(1)-C(6)	108.3(8)
C(6)-C(1)-C(10)	107.8(6)	O(2)-C(8)-C(7)	106.8(8)	C(9)-C(14)-C(15)	125.5(8)	C(22)-C(21)-C(27)	113.4(12)	H(26B)-C(26)-H(26C)	109.5
C(3)-C(2)-C(1)	113.9(6)	C(9)-C(8)-C(7)	111.7(7)	C(13)-C(14)-C(15)	108.9(8)	C(22)-C(21)-C(17)	110.3(9)	C(21)-C(27)-H(27A)	109.5
C(3)-C(2)-H(2A)	108.8	O(2)-C(8)-H(8)	109.2	C(16)-C(15)-C(14)	105.1(7)	C(27)-C(21)-C(17)	113.1(9)	C(21)-C(27)-H(27B)	109.5
C(1)-C(2)-H(2A)	108.8	C(9)-C(8)-H(8)	109.2	C(16)-C(15)-H(15A)	110.7	C(22)-C(21)-H(21)	106.5	H(27A)-C(27)-H(27B)	109.5
C(3)-C(2)-H(2B)	108.8	C(7)-C(8)-H(8)	109.2	C(14)-C(15)-H(15A)	110.7	C(27)-C(21)-H(21)	106.5	C(21)-C(27)-H(27C)	109.5
C(1)-C(2)-H(2B)	108.8	C(4)-O(1)-H(1)	109.5	C(16)-C(15)-H(15B)	110.7	C(17)-C(21)-H(21)	106.5	H(27A)-C(27)-H(27C)	109.5
H(2A)-C(2)-H(2B)	107.7	C(14)-C(9)-C(8)	123.9(7)	C(14)-C(15)-H(15B)	110.7	C(21)-C(22)-C(23)	113.8(10)	H(27B)-C(27)-H(27C)	109.5
C(4)-C(3)-C(2)	112.3(7)	C(14)-C(9)-C(10)	124.0(7)	H(15A)-C(15)-H(15B)	108.8	C(21)-C(22)-H(22A)	108.8	C(29)-C(28)-C(30)	110.3(14)
C(4)-C(3)-H(3A)	109.1	C(8)-C(9)-C(10)	112.1(8)	C(15)-C(16)-C(17)	106.2(8)	C(23)-C(22)-H(22A)	108.8	C(29)-C(28)-C(24)	114.3(11)
C(2)-C(3)-H(3A)	109.1	C(8)-O(2)-H(2)	109.5	C(15)-C(16)-H(16A)	110.5	C(21)-C(22)-H(22B)	108.8	C(30)-C(28)-C(24)	114.6(13)
C(4)-C(3)-H(3B)	109.1	C(11)-C(10)-C(9)	111.8(7)	C(17)-C(16)-H(16A)	110.5	C(23)-C(22)-H(22B)	108.8	C(29)-C(28)-H(28)	105.6
C(2)-C(3)-H(3B)	109.1	C(11)-C(10)-C(1)	113.1(7)	C(15)-C(16)-H(16B)	110.5	H(22A)-C(22)-H(22B)	107.7	C(30)-C(28)-H(28)	105.6

H(3A)-C(3)-H(3B)	107.9	C(9)-C(10)-C(1)	111.8(5)	C(17)-C(16)-H(16B)	110.5	C(24)-C(23)-C(22)	110.6(9)	C(24)-C(28)-H(28)	105.6
O(1)-C(4)-C(3)	110.0(6)	C(11)-C(10)-H(10)	106.5	H(16A)-C(16)-H(16B)	108.7	C(24)-C(23)-H(23A)	109.5	C(28)-C(29)-H(29A)	109.5
O(1)-C(4)-C(5)	112.2(6)	C(9)-C(10)-H(10)	106.5	C(16)-C(17)-C(13)	103.3(8)	C(22)-C(23)-H(23A)	109.5	C(28)-C(29)-H(29B)	109.5
C(3)-C(4)-C(5)	111.6(9)	C(1)-C(10)-H(10)	106.5	C(16)-C(17)-C(21)	112.5(8)	C(24)-C(23)-H(23B)	109.5	H(29A)-C(29)-H(29B)	109.5
O(1)-C(4)-H(4)	107.6	C(10)-C(11)-C(12)	114.3(7)	C(13)-C(17)-C(21)	119.2(8)	C(22)-C(23)-H(23B)	109.5	C(28)-C(29)-H(29C)	109.5
C(3)-C(4)-H(4)	107.6	C(10)-C(11)-H(11A)	108.7	C(16)-C(17)-H(17)	107.0	H(23A)-C(23)-H(23B)	108.1	H(29A)-C(29)-H(29C)	109.5
C(5)-C(4)-H(4)	107.6	C(12)-C(11)-H(11A)	108.7	C(13)-C(17)-H(17)	107.0	C(23)-C(24)-C(28)	110.8(10)	H(29B)-C(29)-H(29C)	109.5
C(19)-C(5)-C(4)	124.1(7)	C(10)-C(11)-H(11B)	108.7	C(21)-C(17)-H(17)	107.0	C(23)-C(24)-C(25)	109.9(12)	C(28)-C(30)-H(30A)	109.5
C(19)-C(5)-C(6)	123.4(7)	C(12)-C(11)-H(11B)	108.7	C(1)-C(18)-H(18A)	109.5	C(28)-C(24)-C(25)	113.4(11)	C(28)-C(30)-H(30B)	109.5
C(4)-C(5)-C(6)	112.6(5)	H(11A)-C(11)-H(11B)	107.6	C(1)-C(18)-H(18B)	109.5	C(23)-C(24)-H(24)	107.5	H(30A)-C(30)-H(30B)	109.5
C(7)-C(6)-C(5)	114.0(5)	C(13)-C(12)-C(11)	111.9(7)	H(18A)-C(18)-H(18B)	109.5	C(28)-C(24)-H(24)	107.5	C(28)-C(30)-H(30C)	109.5
C(7)-C(6)-C(1)	111.8(7)	C(13)-C(12)-H(12A)	109.2	C(1)-C(18)-H(18C)	109.5	C(25)-C(24)-H(24)	107.5	H(30A)-C(30)-H(30C)	109.5
C(5)-C(6)-C(1)	111.9(6)	C(11)-C(12)-H(12A)	109.2	H(18A)-C(18)-H(18C)	109.5	C(26)-C(25)-C(24)	111.1(14)	H(30B)-C(30)-H(30C)	109.5
C(7)-C(6)-H(6)	106.2	C(13)-C(12)-H(12B)	109.2	H(18B)-C(18)-H(18C)	109.5	C(26)-C(25)-H(25A)	109.4	C(48)-C(31)-C(32)	110.3(7)
C(6)-C(1)-C(10)	107.8(6)	O(2)-C(8)-C(7)	106.8(8)	C(9)-C(14)-C(15)	125.5(8)	C(22)-C(21)-C(27)	113.4(12)	H(26B)-C(26)-H(26C)	109.5
C(3)-C(2)-C(1)	113.9(6)	C(9)-C(8)-C(7)	111.7(7)	C(13)-C(14)-C(15)	108.9(8)	C(22)-C(21)-C(17)	110.3(9)	C(21)-C(27)-H(27A)	109.5
C(3)-C(2)-H(2A)	108.8	O(2)-C(8)-H(8)	109.2	C(16)-C(15)-C(14)	105.1(7)	C(27)-C(21)-C(17)	113.1(9)	C(21)-C(27)-H(27B)	109.5
C(1)-C(2)-H(2A)	108.8	C(9)-C(8)-H(8)	109.2	C(16)-C(15)-H(15A)	110.7	C(22)-C(21)-H(21)	106.5	H(27A)-C(27)-H(27B)	109.5
C(3)-C(2)-H(2B)	108.8	C(7)-C(8)-H(8)	109.2	C(14)-C(15)-H(15A)	110.7	C(27)-C(21)-H(21)	106.5	C(21)-C(27)-H(27C)	109.5
C(1)-C(2)-H(2B)	108.8	C(4)-O(1)-H(1)	109.5	C(16)-C(15)-H(15B)	110.7	C(17)-C(21)-H(21)	106.5	H(27A)-C(27)-H(27C)	109.5
C(5)-C(6)-H(6)	106.2	C(11)-C(12)-H(12B)	109.2	C(5)-C(19)-H(19A)	120.0	C(24)-C(25)-H(25A)	109.4	C(48)-C(31)-C(36)	111.3(6)
C(1)-C(6)-H(6)	106.2	H(12A)-C(12)-H(12B)	107.9	C(5)-C(19)-H(19B)	120.0	C(26)-C(25)-H(25B)	109.4	C(32)-C(31)-C(36)	109.4(8)
C(6)-C(7)-C(8)	111.0(5)	C(14)-C(13)-C(12)	108.2(8)	H(19A)-C(19)-H(19B)	120.0	C(24)-C(25)-H(25B)	109.4	C(48)-C(31)-C(40)	110.3(8)
C(6)-C(7)-H(7A)	109.4	C(14)-C(13)-C(20)	109.0(8)	C(13)-C(20)-H(20A)	109.5	H(25A)-C(25)-H(25B)	108.0	C(32)-C(31)-C(40)	109.3(5)
C(8)-C(7)-H(7A)	109.4	C(12)-C(13)-C(20)	111.1(9)	C(13)-C(20)-H(20B)	109.5	C(25)-C(26)-H(26A)	109.5	C(36)-C(31)-C(40)	106.2(6)
C(6)-C(7)-H(7B)	109.4	C(14)-C(13)-C(17)	101.6(7)	H(20A)-C(20)-H(20B)	109.5	C(25)-C(26)-H(26B)	109.5	C(33)-C(32)-C(31)	113.6(6)
C(8)-C(7)-H(7B)	109.4	C(12)-C(13)-C(17)	115.0(8)	C(13)-C(20)-H(20C)	109.5	H(26A)-C(26)-H(26B)	109.5	C(33)-C(32)-H(32A)	108.8
H(7A)-C(7)-H(7B)	108.0	C(20)-C(13)-C(17)	111.4(9)	H(20A)-C(20)-H(20C)	109.5	C(25)-C(26)-H(26C)	109.5	C(31)-C(32)-H(32A)	108.8
O(2)-C(8)-C(9)	110.9(6)	C(9)-C(14)-C(13)	125.1(7)	H(20B)-C(20)-H(20C)	109.5	H(26A)-C(26)-H(26C)	109.5	C(33)-C(32)-H(32B)	108.8
C(31)-C(32)-H(32B)	108.8	C(42)-C(41)-H(41A)	108.8	C(46)-C(47)-H(47)	107.2	H(53A)-C(53)-H(53B)	107.7	H(26B)-C(26)-H(26C)	109.5
H(32A)-C(32)-H(32B)	107.7	C(40)-C(41)-H(41A)	108.8	C(51)-C(47)-H(47)	107.2	C(55)-C(54)-C(58)	114.9(13)	C(21)-C(27)-H(27A)	109.5
C(34)-C(33)-C(32)	109.7(7)	C(42)-C(41)-H(41B)	108.8	C(43)-C(47)-H(47)	107.2	C(55)-C(54)-C(53)	106.8(12)	C(21)-C(27)-H(27B)	109.5
C(34)-C(33)-H(33A)	109.7	C(40)-C(41)-H(41B)	108.8	C(31)-C(48)-H(48A)	109.5	C(58)-C(54)-C(53)	113.8(12)	H(27A)-C(27)-H(27B)	109.5
C(32)-C(33)-H(33A)	109.7	H(41A)-C(41)-H(41B)	107.7	C(31)-C(48)-H(48B)	109.5	C(55)-C(54)-H(54)	107.0	C(21)-C(27)-H(27C)	109.5
C(34)-C(33)-H(33B)	109.7	C(41)-C(42)-C(43)	109.4(7)	H(48A)-C(48)-H(48B)	109.5	C(58)-C(54)-H(54)	107.0	H(27A)-C(27)-H(27C)	109.5
C(32)-C(33)-H(33B)	109.7	C(41)-C(42)-H(42A)	109.8	C(31)-C(48)-H(48C)	109.5	C(53)-C(54)-H(54)	107.0	H(27B)-C(27)-H(27C)	109.5

H(33A)-C(33)-H(33B)	108.2	C(43)-C(42)-H(42A)	109.8	H(48A)-C(48)-H(48C)	109.5	C(56)-C(55)-C(54)	116.0(19)	C(29)-C(28)-C(30)	110.3(14)
O(3)-C(34)-C(33)	110.8(7)	C(41)-C(42)-H(42B)	109.8	H(48B)-C(48)-H(48C)	109.5	C(56)-C(55)-H(55A)	108.3	C(29)-C(28)-C(24)	114.3(11)
O(3)-C(34)-C(35)	112.6(7)	C(43)-C(42)-H(42B)	109.8	C(34)-O(3)-H(3)	109.5	C(54)-C(55)-H(55A)	108.3	C(30)-C(28)-C(24)	114.6(13)
C(35)-C(36)-H(36)	106.5	H(42A)-C(42)-H(42B)	108.2	C(35)-C(49)-H(49A)	120.0	C(56)-C(55)-H(55B)	108.3	C(29)-C(28)-H(28)	105.6
C(37)-C(36)-H(36)	106.5	C(44)-C(43)-C(42)	108.7(9)	C(35)-C(49)-H(49B)	120.0	C(54)-C(55)-H(55B)	108.3	H(59A)-C(59)-H(59C)	109.5
C(31)-C(36)-H(36)	106.5	C(44)-C(43)-C(50)	111.7(7)	H(49A)-C(49)-H(49B)	120.0	H(55A)-C(55)-H(55B)	107.4	H(59B)-C(59)-H(59C)	109.5
C(38)-C(37)-C(36)	111.3(5)	C(42)-C(43)-C(50)	113.1(8)	C(38)-O(4)-H(4A)	109.5	C(55)-C(56)-H(56A)	109.5	C(58)-C(60)-H(60A)	109.5
C(38)-C(37)-H(37A)	109.4	C(44)-C(43)-C(47)	100.3(7)	C(43)-C(50)-H(50A)	109.5	C(55)-C(56)-H(56B)	109.5	C(58)-C(60)-H(60B)	109.5
C(36)-C(37)-H(37A)	109.4	C(42)-C(43)-C(47)	113.7(7)	C(43)-C(50)-H(50B)	109.5	H(56A)-C(56)-H(56B)	109.5	H(60A)-C(60)-H(60B)	109.5
C(38)-C(37)-H(37B)	109.4	C(50)-C(43)-C(47)	108.6(9)	H(50A)-C(50)-H(50B)	109.5	C(55)-C(56)-H(56C)	109.5	C(58)-C(60)-H(60C)	109.5
C(36)-C(37)-H(37B)	109.4	C(39)-C(44)-C(43)	124.4(7)	C(43)-C(50)-H(50C)	109.5	H(56A)-C(56)-H(56C)	109.5	H(60A)-C(60)-H(60C)	109.5
C(3)-C(2)-C(1)	113.9(6)	C(9)-C(8)-C(7)	111.7(7)	C(13)-C(14)-C(15)	108.9(8)	C(22)-C(21)-C(17)	110.3(9)	C(21)-C(27)-H(27A)	109.5
C(3)-C(2)-H(2A)	108.8	O(2)-C(8)-H(8)	109.2	C(16)-C(15)-C(14)	105.1(7)	C(27)-C(21)-C(17)	113.1(9)	C(21)-C(27)-H(27B)	109.5
C(1)-C(2)-H(2A)	108.8	C(9)-C(8)-H(8)	109.2	C(16)-C(15)-H(15A)	110.7	C(22)-C(21)-H(21)	106.5	H(27A)-C(27)-H(27B)	109.5
C(3)-C(2)-H(2B)	108.8	C(7)-C(8)-H(8)	109.2	C(14)-C(15)-H(15A)	110.7	C(27)-C(21)-H(21)	106.5	C(21)-C(27)-H(27C)	109.5
C(1)-C(2)-H(2B)	108.8	C(4)-O(1)-H(1)	109.5	C(16)-C(15)-H(15B)	110.7	C(17)-C(21)-H(21)	106.5	H(27A)-C(27)-H(27C)	109.5
H(37A)-C(37)-H(37B)	108.0	C(39)-C(44)-C(45)	125.9(8)	H(50A)-C(50)-H(50C)	109.5	H(56B)-C(56)-H(56C)	109.5	H(60B)-C(60)-H(60C)	109.5
O(4)-C(38)-C(39)	110.9(6)	C(43)-C(44)-C(45)	109.4(8)	H(50B)-C(50)-H(50C)	109.5	C(51)-C(57)-H(57A)	109.5	C(58)-C(60)-H(60B)	109.5
O(4)-C(38)-C(37)	108.3(7)	C(46)-C(45)-C(44)	104.4(7)	C(52)-C(51)-C(47)	113.1(9)	C(51)-C(57)-H(57B)	109.5	H(60A)-C(60)-H(60B)	109.5
C(39)-C(38)-C(37)	112.0(6)	C(46)-C(45)-H(45A)	110.9	C(52)-C(51)-C(57)	109.7(11)	H(57A)-C(57)-H(57B)	109.5	C(58)-C(60)-H(60C)	109.5
O(4)-C(38)-H(38)	108.5	C(44)-C(45)-H(45A)	110.9	C(47)-C(51)-C(57)	111.8(12)	C(51)-C(57)-H(57C)	109.5	H(60A)-C(60)-H(60C)	109.5
C(39)-C(38)-H(38)	108.5	C(46)-C(45)-H(45B)	110.9	C(52)-C(51)-H(51)	107.3	H(57A)-C(57)-H(57C)	109.5	H(60B)-C(60)-H(60C)	109.5
C(37)-C(38)-H(38)	108.5	C(44)-C(45)-H(45B)	110.9	C(47)-C(51)-H(51)	107.3	H(57B)-C(57)-H(57C)	109.5	H(59A)-C(59)-H(59C)	109.5
C(44)-C(39)-C(38)	124.7(6)	H(45A)-C(45)-H(45B)	108.9	C(57)-C(51)-H(51)	107.3	C(59)-C(58)-C(54)	113.2(13)	C(48)-C(31)-C(40)	110.3(8)
C(44)-C(39)-C(40)	122.7(7)	C(47)-C(46)-C(45)	106.4(8)	C(51)-C(52)-C(53)	116.5(11)	C(59)-C(58)-C(60)	109.9(16)	C(32)-C(31)-C(40)	109.3(5)
C(38)-C(39)-C(40)	112.6(7)	C(47)-C(46)-H(46A)	110.4	C(51)-C(52)-H(52A)	108.2	C(54)-C(58)-C(60)	112.3(11)	C(36)-C(31)-C(40)	106.2(6)
C(41)-C(40)-C(39)	112.0(8)	C(45)-C(46)-H(46A)	110.4	C(53)-C(52)-H(52A)	108.2	C(59)-C(58)-H(58)	107.0	C(33)-C(32)-C(31)	113.6(6)
C(41)-C(40)-C(31)	113.6(7)	C(47)-C(46)-H(46B)	110.4	C(51)-C(52)-H(52B)	108.2	C(54)-C(58)-H(58)	107.0	C(33)-C(32)-H(32A)	108.8
C(39)-C(40)-C(31)	111.3(5)	C(45)-C(46)-H(46B)	110.4	C(53)-C(52)-H(52B)	108.2	C(60)-C(58)-H(58)	107.0	C(31)-C(32)-H(32A)	108.8
C(41)-C(40)-H(40)	106.5	H(46A)-C(46)-H(46B)	108.6	H(52A)-C(52)-H(52B)	107.3	C(58)-C(59)-H(59A)	109.5	C(33)-C(32)-H(32B)	108.8
C(39)-C(40)-H(40)	106.5	C(46)-C(47)-C(51)	114.0(9)	C(52)-C(53)-C(54)	113.7(11)	C(58)-C(59)-H(59B)	109.5		
C(31)-C(40)-H(40)	106.5	C(46)-C(47)-C(43)	102.6(6)	C(52)-C(53)-H(53A)	108.8	H(59A)-C(59)-H(59B)	109.5		
C(42)-C(41)-C(40)	113.8(7)	C(51)-C(47)-C(43)	118.0(8)	C(54)-C(53)-H(53A)	108.8	C(58)-C(59)-H(59C)	109.5		

Table S15. Anisotropic displacement parameters (Å ^{2} x 10^3) for 7α-theonellasterol.[a]

	U^{11}	U^{22}	U^{23}	U^{23}	U^{13}	U^{12}
C(1)	46(3)	168(8)	69(4)	-4(4)	21(3)	6(4)
C(2)	50(4)	169(8)	86(5)	-23(5)	24(4)	-7(5)
C(3)	45(4)	191(10)	91(5)	-15(5)	20(4)	17(5)
C(4)	51(4)	190(9)	66(4)	-12(5)	14(3)	12(5)
C(5)	52(3)	165(8)	59(3)	6(4)	16(3)	13(4)
C(6)	51(3)	150(7)	62(3)	5(4)	22(3)	17(4)
C(7)	54(4)	176(9)	70(4)	5(4)	26(3)	12(5)
C(8)	51(4)	177(9)	81(4)	-11(5)	27(3)	1(5)
O(1)	58(3)	225(8)	66(3)	-9(3)	0(2)	34(4)
C(9)	44(3)	152(8)	83(4)	4(5)	26(3)	3(4)
O(2)	60(3)	174(6)	65(3)	-5(3)	-4(2)	20(3)
C(10)	50(4)	155(8)	81(4)	-11(4)	29(3)	1(4)
C(11)	55(4)	166(9)	120(6)	-38(6)	41(4)	-14(5)
C(12)	71(5)	144(8)	119(6)	-36(6)	50(5)	-28(5)
C(13)	60(4)	134(8)	131(7)	-5(6)	42(5)	-19(5)
C(14)	56(4)	135(7)	102(5)	1(5)	36(4)	-6(5)
C(15)	53(4)	166(9)	109(6)	11(6)	29(4)	-9(5)
C(16)	65(5)	131(8)	154(8)	7(7)	40(5)	-13(5)
C(17)	73(5)	119(7)	133(7)	-6(6)	37(5)	-22(5)
C(18)	80(6)	159(9)	109(6)	15(6)	35(5)	18(6)
C(19)	66(4)	212(10)	61(4)	15(5)	27(3)	20(5)
C(20)	79(6)	170(11)	182(11)	44(9)	43(7)	22(7)
C(21)	83(6)	123(8)	173(10)	-30(7)	47(6)	-27(5)
C(22)	101(7)	104(7)	185(11)	-35(7)	26(7)	-14(6)
C(23)	87(7)	103(7)	216(12)	-50(8)	18(7)	-20(6)
C(24)	96(8)	106(8)	217(13)	-38(9)	18(8)	-19(6)
C(25)	96(8)	151(11)	254(18)	-3(11)	25(10)	-42(8)
C(26)	128(12)	178(15)	360(30)	17(15)	89(16)	-8(11)
C(27)	125(10)	213(15)	246(16)	-112(13)	111(11)	-70(10)
C(28)	85(7)	107(8)	217(14)	-11(9)	8(8)	-10(6)
C(29)	166(14)	131(10)	236(17)	-8(11)	82(13)	-4(10)
C(30)	124(10)	109(8)	280(20)	-43(10)	-3(12)	2(8)
C(31)	47(3)	176(8)	53(3)	12(4)	15(3)	5(4)
C(32)	74(5)	235(12)	57(4)	5(5)	24(4)	3(6)
C(33)	77(5)	227(12)	56(4)	1(5)	18(4)	12(6)
C(34)	60(4)	187(10)	68(4)	-8(5)	11(3)	22(5)
C(35)	57(4)	155(8)	70(4)	-2(4)	18(3)	18(5)
C(36)	53(4)	159(8)	55(3)	7(4)	13(3)	11(4)
C(37)	60(4)	149(7)	59(3)	3(4)	20(3)	1(4)
C(38)	49(3)	169(8)	48(3)	4(4)	10(3)	-5(4)
C(39)	52(4)	154(7)	57(3)	5(4)	20(3)	2(4)
C(40)	61(4)	166(8)	63(4)	-1(4)	30(3)	-4(5)
C(41)	85(6)	224(12)	82(5)	21(6)	43(5)	-14(7)
C(42)	65(5)	209(11)	92(5)	11(6)	35(4)	-5(6)
C(43)	63(4)	169(9)	88(5)	23(5)	27(4)	7(5)
C(44)	53(4)	147(7)	74(4)	25(5)	24(3)	16(5)
C(45)	58(4)	167(8)	72(4)	-18(5)	19(3)	1(5)
C(46)	65(5)	154(8)	94(5)	1(5)	19(4)	15(5)
C(47)	59(4)	149(8)	97(5)	8(5)	17(4)	-1(5)

C(48)	73(5)	181(10)	78(5)	30(5)	22(4)	28(6)
O(3)	56(3)	201(7)	78(3)	-12(4)	-5(2)	30(4)
C(49)	61(5)	190(10)	93(5)	-11(6)	24(4)	-2(5)
O(4)	67(3)	165(5)	69(3)	7(3)	1(2)	17(4)
C(50)	84(6)	182(11)	124(8)	50(8)	19(5)	21(7)
C(51)	76(6)	164(10)	138(8)	25(7)	16(6)	-2(7)
C(52)	83(6)	136(9)	143(9)	20(7)	-1(6)	0(6)
C(53)	105(8)	117(9)	228(15)	46(9)	7(9)	-13(7)
C(54)	69(5)	111(8)	189(11)	15(7)	-3(7)	-2(6)
C(55)	111(11)	193(16)	310(30)	-76(19)	43(15)	-12(11)
C(56)	280(30)	164(18)	510(60)	10(30)	230(40)	49(19)
C(57)	174(16)	340(30)	189(15)	-17(16)	85(13)	-126(17)
C(58)	84(7)	129(10)	262(17)	39(10)	28(9)	4(7)
C(59)	104(10)	198(15)	330(30)	63(16)	78(13)	28(10)
C(60)	145(13)	132(11)	350(30)	59(14)	15(16)	-8(10)

[a]The anisotropic displacement factor exponent takes the form: $-2\pi^2[h^2 a^{*2}U^{11} + ... + 2 h k a^* b^* U^{12}]$

Table S16. Hydrogen coordinates (x 10^4) and isotropic displacement parameters (Å ^{2} x 10^3) for 7α-theonellasterol.

	x	y	z	U(eq)		x	y	z	U(eq)
H(2A)	9035	4282	12558	122	H(19B)	4249	4583	15976	134
H(2B)	8287	4597	12059	122	H(20A)	4697	3364	8954	215
H(3A)	9474	4365	15482	132	H(20B)	3153	3422	9815	215
H(3B)	10487	4612	14805	132	H(20C)	5122	3565	10605	215
H(4)	8123	4925	14575	124	H(21)	2289	3250	6406	150
H(6)	5241	4734	12397	104	H(22A)	151	3519	3296	162
H(7A)	2592	4633	13141	119	H(22B)	-675	3358	4587	162
H(7B)	3169	4305	13409	119	H(23A)	1104	3092	2370	172
H(8)	1006	4352	10618	122	H(23B)	901	2918	3974	172
H(1)	9982	4713	17520	182	H(24)	-2278	3163	1520	177
H(2)	1167	4709	9134	159	H(25A)	-2152	2667	3643	210
H(10)	5378	4473	9949	112	H(25B)	-2431	2980	4273	210
H(11A)	6947	3926	10559	133	H(26A)	-5151	3009	1623	329
H(11B)	7351	4177	9423	133	H(26B)	-5366	2811	3128	329
H(12A)	4648	4070	7117	128	H(26C)	-4967	2672	1513	329
H(12B)	5860	3788	7626	128	H(27A)	3873	3253	4381	274
H(15A)	190	3824	9655	131	H(27B)	4919	3437	6048	274
H(15B)	-268	4066	8195	131	H(27C)	3757	3592	4303	274
H(16A)	-909	3706	6303	139	H(28)	-2902	2715	17	176
H(16B)	141	3480	7753	139	H(29A)	168	2964	-583	260
H(17)	1717	3853	5854	129	H(29B)	-1802	3110	-1203	260
H(18A)	6592	4088	14719	173	H(29C)	-1516	2807	-1956	260
H(18B)	7337	3923	13381	173	H(30A)	837	2548	1208	281
H(18C)	5187	3949	13027	173	H(30B)	-908	2357	257	281
H(19A)	6134	4709	17367	134	H(30C)	-488	2444	2229	281
H(32A)	-4157	5807	2288	146	H(4A)	-785	5325	9271	158
H(32B)	-3595	5485	2810	146	H(50A)	-2617	6463	6450	200
H(33A)	-7290	5692	1530	145	H(50B)	-820	6655	6947	200
H(33B)	-6363	5486	500	145	H(50C)	-1793	6591	8350	200
H(34)	-6077	5124	2557	129	H(51)	1614	6753	9335	157
H(36)	-4210	5278	5443	108	H(52A)	4785	6505	11757	156
H(37A)	-5714	5640	7455	107	H(52B)	3224	6662	12286	156
H(37B)	-5328	5308	7808	107	H(53A)	4039	7097	11068	194
H(38)	-2929	5604	9670	108	H(53B)	5778	6930	10922	194
H(40)	-1665	5565	5751	114	H(54)	6545	6849	14086	161
H(41A)	-948	5903	4184	151	H(55A)	4211	7328	13811	251
H(41B)	-2238	6136	4613	151	H(55B)	3549	7011	13939	251
H(42A)	874	6261	5972	143	H(56A)	5782	6976	16646	438
H(42B)	1248	5964	6979	143	H(56B)	4242	7207	16549	438
H(45A)	-2100	6131	10777	119	H(56C)	6205	7306	16512	438
H(45B)	-472	5902	11291	119	H(57A)	4318	6411	8525	341
H(46A)	-146	6499	11101	128	H(57B)	4206	6749	8334	341
H(46B)	1379	6280	12212	128	H(57C)	2593	6556	7136	341
H(47)	2247	6156	9895	125	H(58)	8111	7292	14853	198
H(48A)	-5314	6113	3975	167	H(59A)	9471	6911	13844	316
H(48B)	-5145	6042	5924	167	H(59B)	10231	7215	13547	316
H(48C)	-6817	5919	4377	167	H(59C)	8798	7052	11973	316
H(3)	-9450	5312	965	178	H(60A)	7986	7646	12878	339

H(49A)	-9566	5266	3967	138	H(60B)	5900	7578	12658	339
H(49B)	-8376	5370	5921	138	H(60C)	6791	7456	11296	339

Table S17. Hydrogen bonds for 7α-theonellasterol [Å and °].[a]

D-H...A	d(D-H)	d(H...A)	d(D...A)	<(DHA)
O(4)-H(4A)...O(3)#1	0.83	2.03	2.732(7)	142.2
O(1)-H(1)...O(2)#1	0.83	2.13	2.764(7)	133.4
O(2)-H(2)...O(1)#2	0.83	1.96	2.764(7)	164.4
O(3)-H(3)...O(4)#2	0.83	2.17	2.732(7)	124.8

[a]Symmetry transformations used to generate equivalent atoms: #1 x+1, y, z+1   #2 x-1, y, z-1

Table S18. Torsion angles [°] for 7α-theonellasterol.

C(18)-C(1)-C(2)-C(3)	-67.5(11)	C(6)-C(1)-C(10)-C(9)	-56.9(10)
C(6)-C(1)-C(2)-C(3)	53.0(11)	C(9)-C(10)-C(11)-C(12)	27.6(12)
C(10)-C(1)-C(2)-C(3)	170.5(9)	C(10)-C(11)-C(12)-C(13)	-56.3(13)
C(1)-C(2)-C(3)-C(4)	-54.1(13)	C(11)-C(12)-C(13)-C(14)	51.1(12)
C(2)-C(3)-C(4)-O(1)	178.4(9)	C(11)-C(12)-C(13)-C(20)	-68.5(12)
C(2)-C(3)-C(4)-C(5)	53.1(11)	C(11)-C(12)-C(13)-C(17)	163.9(9)
O(1)-C(4)-C(5)-C(19)	2.8(15)	C(8)-C(9)-C(14)-C(13)	174.3(8)
C(3)-C(4)-C(5)-C(19)	126.8(10)	C(10)-C(9)-C(14)-C(13)	-4.5(14)
O(1)-C(4)-C(5)-C(6)	-178.6(8)	C(8)-C(9)-C(14)-C(15)	2.6(14)
C(3)-C(4)-C(5)-C(6)	-54.6(10)	C(10)-C(9)-C(14)-C(15)	-176.2(8)
C(19)-C(5)-C(6)-C(7)	2.4(14)	C(12)-C(13)-C(14)-C(9)	-22.9(13)
C(4)-C(5)-C(6)-C(7)	-176.2(9)	C(20)-C(13)-C(14)-C(9)	98.0(11)
C(19)-C(5)-C(6)-C(1)	-125.7(10)	C(17)-C(13)-C(14)-C(9)	-144.3(9)
C(4)-C(5)-C(6)-C(1)	55.7(11)	C(12)-C(13)-C(14)-C(15)	150.0(8)
C(2)-C(1)-C(6)-C(7)	177.6(7)	C(20)-C(13)-C(14)-C(15)	-89.1(10)
C(18)-C(1)-C(6)-C(7)	-62.5(8)	C(17)-C(13)-C(14)-C(15)	28.6(10)
C(10)-C(1)-C(6)-C(7)	58.6(8)	C(9)-C(14)-C(15)-C(16)	164.9(9)
C(2)-C(1)-C(6)-C(5)	-53.1(9)	C(13)-C(14)-C(15)-C(16)	-7.9(10)
C(18)-C(1)-C(6)-C(5)	66.8(9)	C(14)-C(15)-C(16)-C(17)	-16.7(11)
C(10)-C(1)-C(6)-C(5)	-172.1(7)	C(15)-C(16)-C(17)-C(13)	34.4(11)
C(5)-C(6)-C(7)-C(8)	173.8(8)	C(15)-C(16)-C(17)-C(21)	164.4(9)
C(1)-C(6)-C(7)-C(8)	-58.0(10)	C(14)-C(13)-C(17)-C(16)	-38.0(10)
C(6)-C(7)-C(8)-O(2)	-67.5(9)	C(12)-C(13)-C(17)-C(16)	-154.5(9)
C(6)-C(7)-C(8)-C(9)	53.9(11)	C(20)-C(13)-C(17)-C(16)	78.0(10)
O(2)-C(8)-C(9)-C(14)	-112.9(10)	C(14)-C(13)-C(17)-C(21)	-163.7(8)
C(7)-C(8)-C(9)-C(14)	128.2(9)	C(12)-C(13)-C(17)-C(21)	79.7(12)
O(2)-C(8)-C(9)-C(10)	66.1(8)	C(20)-C(13)-C(17)-C(21)	-47.7(11)
C(7)-C(8)-C(9)-C(10)	-52.8(10)	C(16)-C(17)-C(21)-C(22)	53.7(13)
C(14)-C(9)-C(10)-C(11)	2.3(12)	C(13)-C(17)-C(21)-C(22)	174.9(10)
C(8)-C(9)-C(10)-C(11)	-176.7(8)	C(16)-C(17)-C(21)-C(27)	-178.2(13)
C(14)-C(9)-C(10)-C(1)	-125.7(9)	C(13)-C(17)-C(21)-C(27)	-57.0(16)
C(8)-C(9)-C(10)-C(1)	55.3(10)	C(27)-C(21)-C(22)-C(23)	58.9(17)
C(2)-C(1)-C(10)-C(11)	57.9(11)	C(17)-C(21)-C(22)-C(23)	-173.1(11)
C(18)-C(1)-C(10)-C(11)	-63.3(9)	C(21)-C(22)-C(23)-C(24)	163.1(12)
C(6)-C(1)-C(10)-C(11)	175.8(7)	C(22)-C(23)-C(24)-C(28)	156.9(12)
C(2)-C(1)-C(10)-C(9)	-174.8(9)	C(22)-C(23)-C(24)-C(25)	-77.0(15)
C(18)-C(1)-C(10)-C(9)	63.9(9)	C(23)-C(24)-C(25)-C(26)	167.0(13)
C(28)-C(24)-C(25)-C(26)	-68.4(16)	C(48)-C(31)-C(40)-C(41)	-64.9(9)
C(23)-C(24)-C(28)-C(29)	-73.0(15)	C(32)-C(31)-C(40)-C(41)	56.5(12)
C(25)-C(24)-C(28)-C(29)	162.9(12)	C(36)-C(31)-C(40)-C(41)	174.3(8)
C(23)-C(24)-C(28)-C(30)	55.7(16)	C(48)-C(31)-C(40)-C(39)	62.6(9)
C(25)-C(24)-C(28)-C(30)	-68.4(16)	C(32)-C(31)-C(40)-C(39)	-176.0(9)
C(48)-C(31)-C(32)-C(33)	-68.5(12)	C(36)-C(31)-C(40)-C(39)	-58.1(10)
C(36)-C(31)-C(32)-C(33)	54.3(12)	C(39)-C(40)-C(41)-C(42)	29.5(12)
C(40)-C(31)-C(32)-C(33)	170.1(10)	C(31)-C(40)-C(41)-C(42)	156.6(9)
C(31)-C(32)-C(33)-C(34)	-55.8(13)	C(40)-C(41)-C(42)-C(43)	-59.6(13)
C(32)-C(33)-C(34)-O(3)	-177.9(7)	C(41)-C(42)-C(43)-C(44)	55.4(11)
C(32)-C(33)-C(34)-C(35)	56.6(10)	C(41)-C(42)-C(43)-C(50)	-69.3(12)
O(3)-C(34)-C(35)-C(49)	-3.2(15)	C(41)-C(42)-C(43)-C(47)	166.2(9)
C(33)-C(34)-C(35)-C(49)	121.3(11)	C(38)-C(39)-C(44)-C(43)	173.9(7)
O(3)-C(34)-C(35)-C(36)	176.2(8)	C(40)-C(39)-C(44)-C(43)	-4.2(13)

C(33)-C(34)-C(35)-C(36)	-59.2(10)	C(38)-C(39)-C(44)-C(45)	0.1(13)
C(49)-C(35)-C(36)-C(37)	5.0(15)	C(40)-C(39)-C(44)-C(45)	-178.0(7)
C(34)-C(35)-C(36)-C(37)	-174.5(9)	C(42)-C(43)-C(44)-C(39)	-25.6(11)
C(49)-C(35)-C(36)-C(31)	-122.8(11)	C(50)-C(43)-C(44)-C(39)	99.9(11)
C(34)-C(35)-C(36)-C(31)	57.8(10)	C(47)-C(43)-C(44)-C(39)	-145.1(8)
C(48)-C(31)-C(36)-C(35)	68.2(8)	C(42)-C(43)-C(44)-C(45)	149.2(7)
C(32)-C(31)-C(36)-C(35)	-53.9(8)	C(50)-C(43)-C(44)-C(45)	-85.3(9)
C(40)-C(31)-C(36)-C(35)	-171.7(7)	C(47)-C(43)-C(44)-C(45)	29.6(9)
C(48)-C(31)-C(36)-C(37)	-60.2(8)	C(39)-C(44)-C(45)-C(46)	166.7(8)
C(32)-C(31)-C(36)-C(37)	177.7(7)	C(43)-C(44)-C(45)-C(46)	-8.0(9)
C(40)-C(31)-C(36)-C(37)	59.9(8)	C(44)-C(45)-C(46)-C(47)	-18.8(10)
C(35)-C(36)-C(37)-C(38)	175.4(8)	C(45)-C(46)-C(47)-C(51)	165.9(8)
C(31)-C(36)-C(37)-C(38)	-58.0(9)	C(45)-C(46)-C(47)-C(43)	37.1(10)
C(36)-C(37)-C(38)-O(4)	-70.9(8)	C(44)-C(43)-C(47)-C(46)	-40.2(10)
C(36)-C(37)-C(38)-C(39)	51.7(10)	C(42)-C(43)-C(47)-C(46)	-156.0(9)
O(4)-C(38)-C(39)-C(44)	-108.7(9)	C(50)-C(43)-C(47)-C(46)	77.1(10)
C(37)-C(38)-C(39)-C(44)	130.3(8)	C(44)-C(43)-C(47)-C(51)	-166.4(9)
O(4)-C(38)-C(39)-C(40)	69.6(7)	C(42)-C(43)-C(47)-C(51)	77.8(12)
C(37)-C(38)-C(39)-C(40)	-51.5(9)	C(50)-C(43)-C(47)-C(51)	-49.1(11)
C(44)-C(39)-C(40)-C(41)	2.7(12)	C(46)-C(47)-C(51)-C(52)	52.0(13)
C(38)-C(39)-C(40)-C(41)	-175.6(8)	C(43)-C(47)-C(51)-C(52)	172.5(9)
C(44)-C(39)-C(40)-C(31)	-125.7(9)	C(46)-C(47)-C(51)-C(57)	176.4(12)
C(38)-C(39)-C(40)-C(31)	56.0(10)	C(43)-C(47)-C(51)-C(57)	-63.1(14)
C(52)-C(53)-C(54)-C(55)	-76.0(16)	C(55)-C(54)-C(58)-C(59)	165.8(17)
C(52)-C(53)-C(54)-C(58)	156.2(11)	C(53)-C(54)-C(58)-C(59)	-70.6(16)
C(58)-C(54)-C(55)-C(56)	-68(3)	C(55)-C(54)-C(58)-C(60)	-69(2)
C(53)-C(54)-C(55)-C(56)	165(2)	C(53)-C(54)-C(58)-C(60)	55(2)
